# Supplementary material for: Assessing the potential of seaweed extracts to improve vegetative, physiological and berry quality parameters in Vitis vinifera cv. Chardonnay under cool climatic conditions
Source: PLoS One. 2025 Sep 2;20(9):e0331039. doi: 10.1371/journal.pone.0331039 (PMC12404493; doi:10.1371/journal.pone.0331039)
Supplement: S1 Fig — Source: National Geographic Institute see S1 Fig. Reference 1. Licensed under CC BY 4.0 (https://creativecommons.org/licenses/by/4.0/), and (B) the pruning and canopy management schedule. (DOCX) [file pone.0331039.s001.docx]

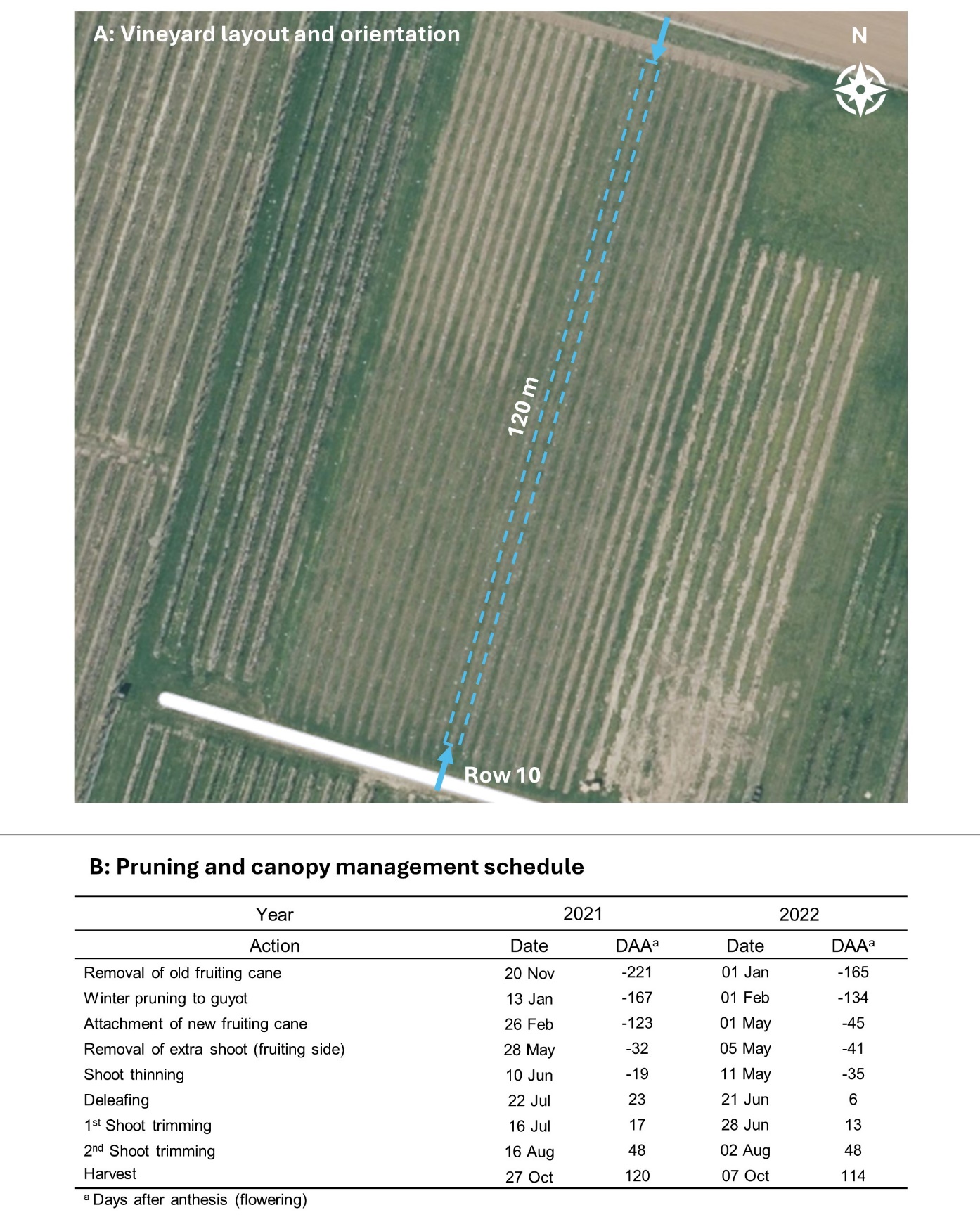


**S****1 Fig. (A) Overview of the vineyard layout and trial row location from orthophoto-based spatial map of Research Station for Fruit Growing. Source: National Geographic Institute [1]. Licensed under CC BY 4.0 (https://creativecommons.org/licenses/by/4.0/), and (B) the pruning and canopy management schedule.**

**References**

1. National Geographic Institute. Orthophoto-based spatial map of Research Station for Fruit Growing [online map]. Brussels: National Geographic Institute; 2022. Available: https://www.geo.be/map?l=en&x=573829.12&y=6581454.45&zoom=19&topic=fed&baseLayer=ngi.cartoweb.topo_bw.be&catalogNodes=1536&layers=orthophoto
