# Supplementary material for: Assessing the potential of seaweed extracts to improve vegetative, physiological and berry quality parameters in Vitis vinifera cv. Chardonnay under cool climatic conditions
Source: PLoS One. 2025 Sep 2;20(9):e0331039. doi: 10.1371/journal.pone.0331039 (PMC12404493; doi:10.1371/journal.pone.0331039)
Supplement: S2 Fig — Odd‑numbered timepoints represent experimental treatments and even‑numbered timepoints the application of a minimal intervention potassium bicarbonate‑based pesticide Karma® (A). Note that samples were only taken from the three central vines of each five‑vine treatment block to reduce the impact of carryover between treatments (indicated with a check mark). Grey panels and blocks were treated only with Karma® (C). (DOCX) [file pone.0331039.s002.docx]

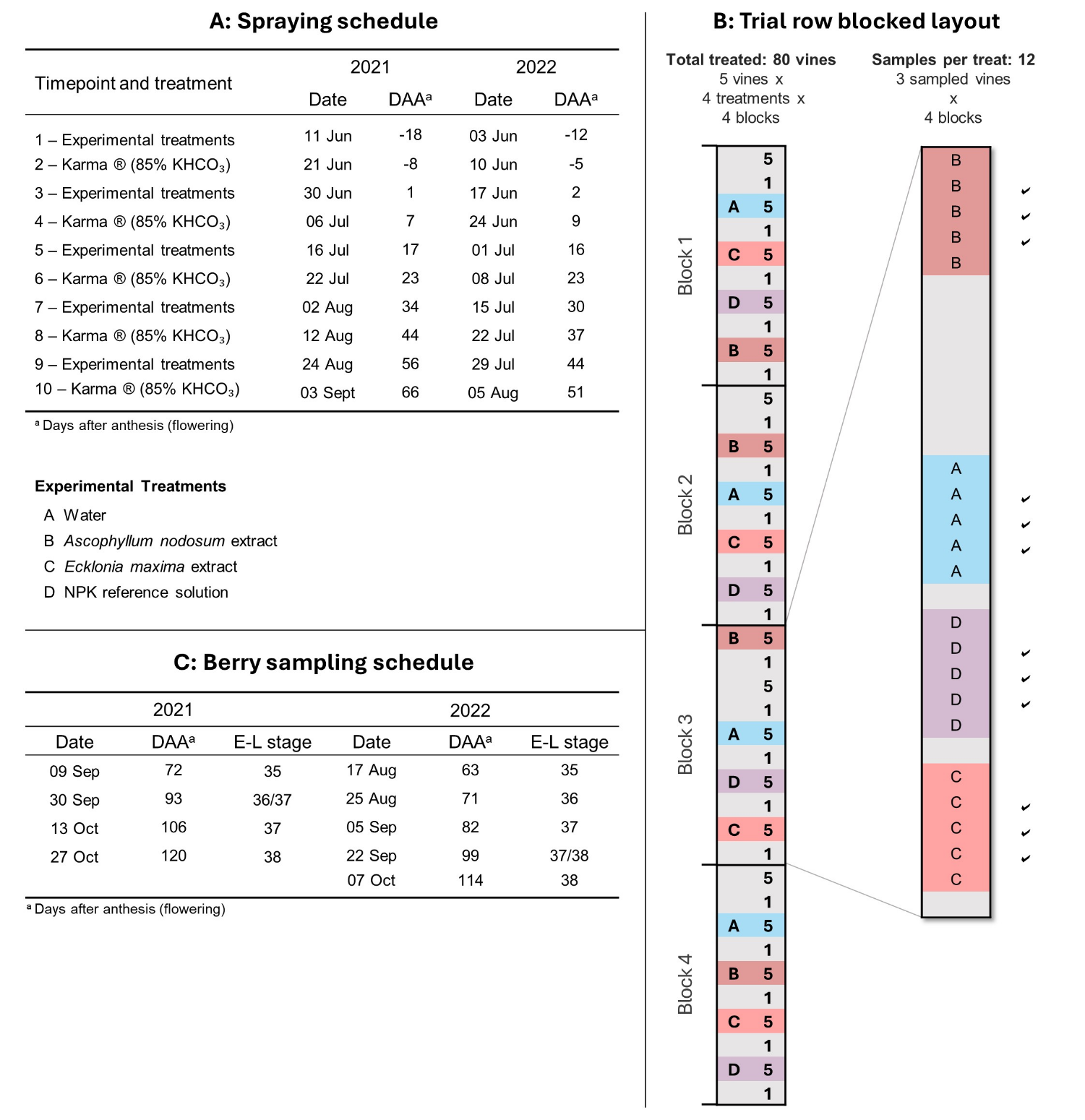


S2 Fig. Overview of the spraying schedule (A), the experimental randomized block design (B) and the berry sampling schedule (C). Odd-numbered timepoints represent experimental treatments and even-numbered timepoints the application of a minimal intervention potassium bicarbonate-based pesticide Karma® (A). Note that samples were only taken from the three central vines of each five-vine treatment block to reduce the impact of carryover between treatments (indicated with a check mark). Grey panels and blocks were treated only with Karma® (C).
