# Supplementary material for: Assessing the potential of seaweed extracts to improve vegetative, physiological and berry quality parameters in Vitis vinifera cv. Chardonnay under cool climatic conditions
Source: PLoS One. 2025 Sep 2;20(9):e0331039. doi: 10.1371/journal.pone.0331039 (PMC12404493; doi:10.1371/journal.pone.0331039)
Supplement: S3 Fig — VPD is shown as a dashed line and temperature as a solid line for both growing seasons (B and C). (DOCX) [file pone.0331039.s003.docx]

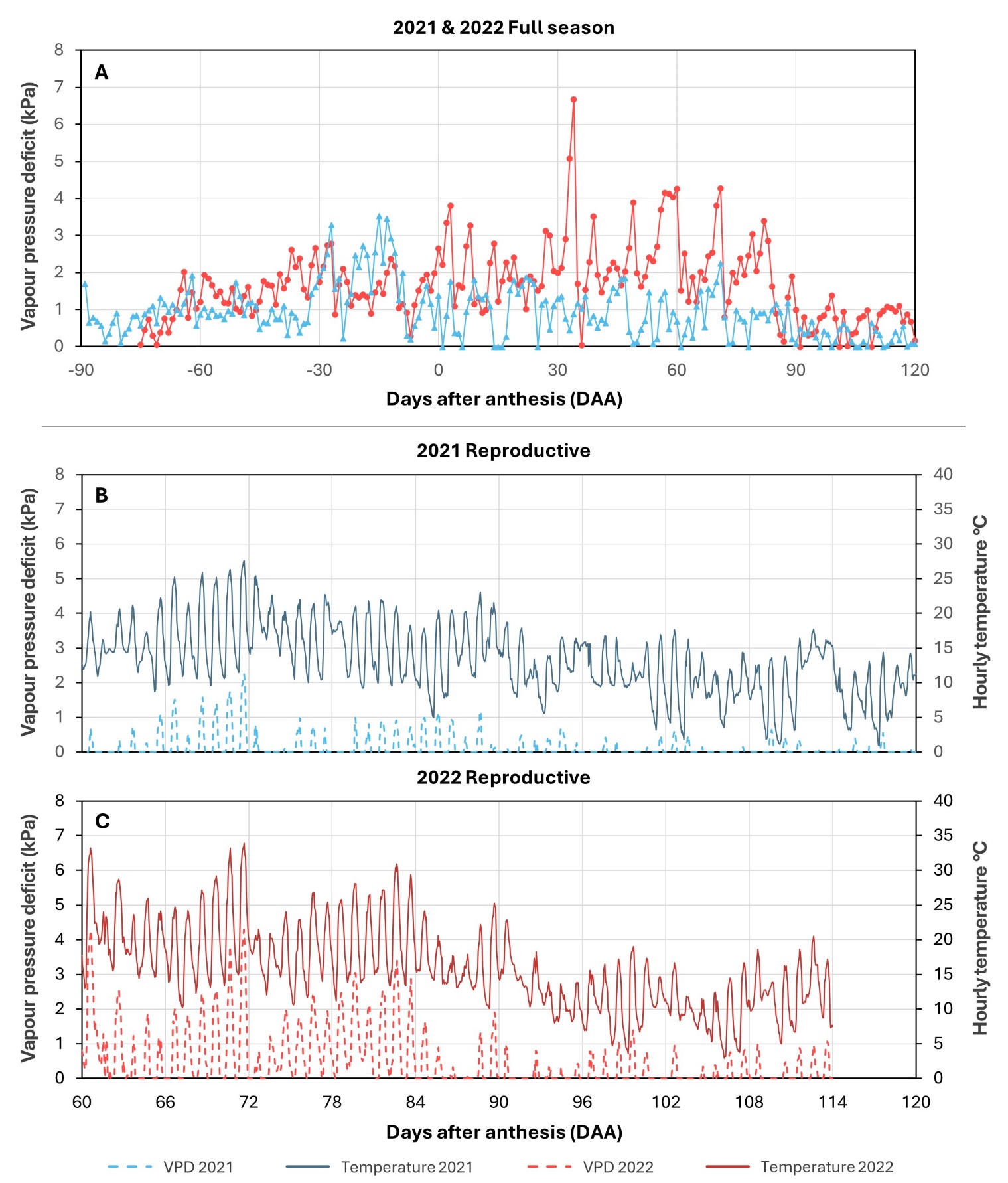


S3 Fig. Comparison of the maximum daily vapour pressure deficit (VPD) (A), and hourly VPD and temperature profiles for the reproductive growth stages of 2021 (B) and 2022 (C). VPD is shown as a dashed line and temperature as a solid line for both growing seasons (B and C).
