# Supplementary material for: Assessing the potential of seaweed extracts to improve vegetative, physiological and berry quality parameters in Vitis vinifera cv. Chardonnay under cool climatic conditions
Source: PLoS One. 2025 Sep 2;20(9):e0331039. doi: 10.1371/journal.pone.0331039 (PMC12404493; doi:10.1371/journal.pone.0331039)
Supplement: S4 Fig — Chardonnay treated with a water control, an A. nodosum extract, an E. maxima extract, and an NPK‑Ref treatment. (DOCX) [file pone.0331039.s004.docx]

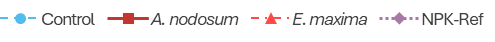


**S4 Fig. Average shoot length in 2021 (A) and 2022 (B) of *V. vinifera* cv. Chardonnay treated with a water control, an *A. nodosum* extract, an *E. maxima* extract and an NPK-reference treatment.**

Average shoot length was determined by measuring three representative shoots per replicate vine (*n* = 12).
