# Supplementary material for: Assessing the potential of seaweed extracts to improve vegetative, physiological and berry quality parameters in Vitis vinifera cv. Chardonnay under cool climatic conditions
Source: PLoS One. 2025 Sep 2;20(9):e0331039. doi: 10.1371/journal.pone.0331039 (PMC12404493; doi:10.1371/journal.pone.0331039)
Supplement: S1 Table — (DOCX) [file pone.0331039.s005.docx]

**S1 Table. Overview of the nutrient composition of the applied seaweed extracts in relation to the NPK-reference treatment.**

| **Parameter** | **Units** | ***Ascophyllum nodosum (*AN)** | ***Ecklonia maxima* (EN)** | **NPK-Ref ^a^** | **AN/EN ^b^** |
| --- | --- | --- | --- | --- | --- |
| Dry matter | % | 28.8 | 2.5 |  | 12 |
| Organic matter / Ash | % | 13.5 | 0.684 |  | 20 |
| Volume density | kg/L | 1 |  |  |  |
| Conductivity | uS/cm | 23000 | 17800 |  |  |
| pH | - | 7.3 | 4.46 |  |  |
| Total Nitrogen (N) | % | 0.198 | 0.278 | 0.2 | 0.7 |
| **Nitrogen dosage (N)^c^** | **kg/ha** | **0.005** | **0.007** | **0.005** | **0.7** |
| Nitrate | % | 0.04 | 0.0019 |  | 21 |
| Ammonium | % | 0.01 | 0.32 |  | 0.03 |
| Neutralising value | % | 3.65 |  |  |  |
| Chlorides | % | 0.81 | 0.067 |  | 12 |
| Phosphorus (P) | % | 0.05 | 0.59 | 1 | 0.09 |
| **Phosphorous dosage (P)^c^** | **kg/ha** | **0.0013** | **0.0148** | **0.025** | **0.09** |
| Potassium (K) | % | 5.6 | 0.039 | 6 | 144 |
| **Potassium dosage (K)^c^** | **kg/ha** | **0.14** | **0.00098** | **0.15** | **144** |
| Magnesium | % | 0.146 | 0.00199 |  | 73 |
| Calcium | % | 0.113 | 0.00206 |  | 55 |
| Sodium | % | 1.12 | 0.028 |  | 40 |
| Sulphur | % | 0.78 | 0.0133 |  | 59 |

Chemical analyses for seaweed extracts conducted by the Belgian Soil Service (Bodemkundige Dienst van België v.z.w.) following the Belgian Accreditation Institution (BELAC) reference methods.

^a^ Note that the NPK reference values were calculated.

^b^ Ratio of nutrients in *Ascophyllum nodosum* extract to *Ecklonia maxima* extract.

^c^ Based on supplier recommendation of 2.5 kg/ha extract per application (5 application timepoints), at a vineyard planting density of 5000 vines/ha – units of kg/ha are equivalent to g/vine.
