# Supplementary material for: Assessing the potential of seaweed extracts to improve vegetative, physiological and berry quality parameters in Vitis vinifera cv. Chardonnay under cool climatic conditions
Source: PLoS One. 2025 Sep 2;20(9):e0331039. doi: 10.1371/journal.pone.0331039 (PMC12404493; doi:10.1371/journal.pone.0331039)
Supplement: S2 Table — Chardonnay over two growing seasons. (DOCX) [file pone.0331039.s006.docx]

S2 Table. Effects of treatment, development stage (time), season (2021 vs 2022), their interactions and covariates block and vapour pressure deficit (VPD) on the leaf anatomical and photosynthetic parameters of *V. vinifera* cv. Chardonnay over two growing seasons.

| Response variable | | Season | | Treatment | | Time | | Season × Treatment | | Season × Time | | Treatment × Time | | Block | | VPD | | |  |
| --- | --- | --- | --- | --- | --- | --- | --- | --- | --- | --- | --- | --- | --- | --- | --- | --- | --- | --- | --- |
|  | **Leaf anatomy** | | | | | | | | | | | | | | | | |  | |
|  | | **F-val** | ***P*-val** | **F-val** | ***P*-val** | **F-val** | ***P*-val** | **F-val** | ***P*-val** | **F-val** | ***P*-val** | **F-val** | ***P*-val** | **F-val** | ***P*-val** | **F-val** | ***P*-val** | | |
| Leaf area (cm^2^) | | 45.70 | *<.001 ^***^* | 5.524 | *0.002 ^**^* | 7.760 | *<.001 ^***^* | 1.771 | *0.153* | 0.304 | *0.582* | 0.369 | *0.949* | 1.628 | *0.198* | 0.019 | *0.891* | | |
| Leaf dry mass (mg) | | 31.13 | *<.001 ^***^* | 2.525 | *0.068* | 3.618 | *0.014 ^*^* | 1.918 | *0.127* | 0.361 | *0.697* | 0.549 | *0.838* | 0.449 | *0.719* | X |  | | |
| Specific leaf area (cm^2^/g) | | 0.931 | *0.431* | 0.421 | *0.738* | 0.170 | *0.917* | 2.100 | *0.149* | 0.584 | *0.810* | 2.466 | *0.118* | X |  | 0.931 | *0.431* | | |
| Leaf dry matter (DM%) | | 0.229 | *0.633* | 0.895 | *0.451* | 9.646 | *<.001 ^***^* | X |  | X |  | X |  | X |  | 1.631 | *0.203* | | |
| Leaf starch content (%)^1^ | | 33.27 | *<.001 ^***^* | 0.389 | *0.764* | 118.5 | *<.001 ^***^* | X |  | X |  | X |  | X |  | 43.73 | *<.001 ^***^* | | |
|  | **Fluorescence indices and gas exchange** | | | | | | | | | | | | | | | | |  | |
| Chlorophyll content index (CCI) | | 48.42 | *<.001 ^***^* | 3.556 | *0.022 ^*^* | 24.97 | *<.001 ^***^* | 10.54 | *<.001 ^***^* | 3.454 | *0.032 ^*^* | X |  | 9.240 | *<.001 ^***^* | 0.201 | *0.654* | |  |
| Reaction centra / cross section (RC/CS) | | 13.29 | *<.001 ^***^* | 1.522 | *0.220* | 102.1 | *<.001 ^***^* | 6.213 | *<.001 ^***^* | X |  | 1.903 | *0.004 ^**^* | 12.93 | *<.001 ^***^* | 18.61 | *<.001 ^***^* | |  |
| Maximum quantum yield PSII (F_v_/F_m_) | | 13.77 | *<.001 ^***^* | 1.317 | *0.281* | 21.71 | *<.001 ^***^* | X |  | 13.93 | *<.001 ^***^* | 1.404 | *0.139* | X |  | X |  | |  |
| Electron transport efficiency PSII (Φ_E0_) | | 70.76 | *<.001 ^***^* | 0.786 | *0.508* | 31.15 | *<.001 ^***^* | X |  | 19.43 | *<.001 ^***^* | 1.852 | *0.017 ^*^* | X |  | X |  | |  |
| Stomatal conductance (σ) | | 67.40 | *<.001 ^***^* | 0.233 | *0.873* | 11.01 | *<.001 ^***^* | 0.210 | *0.889* | 54.361 | *<.001 ^***^* | 0.401 | *0.979* | 4.893 | *0.005 ^**^* | X |  | |  |

F-statistic values from ANOVA on 12 vines per treatment measured over the course of 12 weeks with biweekly measurements (Linear Mixed Model). Asterisks indicate significant differences between the treatments (0.01 < *P* ≤ 0.05: * ; 0.001 < *P* ≤ 0.01: ** ; *P* ≤ 0.001: ***).

Parameters marked with X were excluded from the final model based on the lowest AICc.

^1^ Model based on log(x+1)-transform
