# Supplementary material for: Assessing the potential of seaweed extracts to improve vegetative, physiological and berry quality parameters in Vitis vinifera cv. Chardonnay under cool climatic conditions
Source: PLoS One. 2025 Sep 2;20(9):e0331039. doi: 10.1371/journal.pone.0331039 (PMC12404493; doi:10.1371/journal.pone.0331039)
Supplement: S3 Table — Chardonnay in response to treatment with water as control, an A. nodosum extract, an E. maxima extract, and an NPK‑Ref treatment. Each value represents the mean ± standard error of the raw data (n = 12). Within each development stage for each season, treatments that showed significantly different responses are indicated with different letters based on their estimated marginal means (P < 0.05). (DOCX) [file pone.0331039.s007.docx]

S3 Table. Average leaf area and leaf dry mass of *V. vinifera* cv. Chardonnay in response to treatment with water as control, an *A. nodosum* extract, an *E. maxima* extract, and an NPK-reference treatment. Each value represents the mean ± standard error of the raw data (*n* = 12). Within each development stage for each season, treatments that showed significantly different responses are indicated with different letters based on their estimated marginal means (*P* < 0.05).

| Year | DAA | E-L stage | Control | | *A. nodosum* | | *E. maxima* | | NPK-Ref | |
| --- | --- | --- | --- | --- | --- | --- | --- | --- | --- | --- |
| **Leaf area (cm²)** | | | | | | | | | | |
| 2021 | 5 | 25 | 169 ± 5 | ^a^ | 187 ± 10 | ^a^ | 177 ± 6 | ^a^ | 172 ± 10 | ^a^ |
|  | 20 | 31 | 182 ± 8 | ^a^ | 210 ± 12 | ^a^ | 190 ± 8 | ^a^ | 197 ± 9 | ^a^ |
|  | 37 | 33 | 179 ± 5 | ^a^ | 201 ± 10 | ^a^ | 194 ± 9 | ^a^ | 187 ± 8 | ^a^ |
|  | **Average** | | **177 ± 4** | **^b^** | **199 ± 6** | **^a^** | **187 ± 5** | **^ab^** | **185 ± 5** | **^ab^** |
| 2022 | 2 | 25 | 126 ± 7 | ^a^ | 145 ± 8 | ^a^ | 154 ± 8 | ^a^ | 156 ± 8 | ^a^ |
|  | 21 | 31 | 143 ± 6 | ^a^ | 160 ± 10 | ^a^ | 151 ± 7 | ^a^ | 168 ± 10 | ^a^ |
|  | 43 | 33 | 138 ± 8 | ^ab^ | 166 ± 8 | ^a^ | 134 ± 8 | ^b^ | 160 ± 15 | ^ab^ |
|  | 63 | 35 | 153 ± 7 | ^a^ | 173 ± 9 | ^a^ | 164 ± 9 | ^a^ | 176 ± 9 | ^a^ |
|  | **Average** | | **140 ± 4** | **^b^** | **161 ± 4** | **^a^** | **151 ± 4** | **^ab^** | **165 ± 5** | **^a^** |
| **Leaf dry mass (mg)** | | | | | | | | | | |
| 2021 | 5 | 25 | 1099 ± 54 | ^a^ | 1242 ± 75 | ^a^ | 1183 ± 58 | ^a^ | 1069 ± 88 | ^a^ |
|  | 20 | 31 | 1198 ± 88 | ^a^ | 1296 ± 78 | ^a^ | 1394 ± 97 | ^a^ | 1194 ± 61 | ^a^ |
|  | 37 | 33 | 1114 ± 55 | ^a^ | 1323 ± 77 | ^a^ | 1253 ± 79 | ^a^ | 1247 ± 74 | ^a^ |
|  | **Average** | | **1137 ± 39** | **^a^** | **1287 ± 43** | **^a^** | **1277 ± 47** | **^a^** | **1170 ± 44** | **^a^** |
| 2022 | 2 | 25 | 781 ± 46 | ^a^ | 886 ± 77 | ^a^ | 990 ± 64 | ^a^ | 1028 ± 84 | ^a^ |
|  | 21 | 31 | 943 ± 49 | ^a^ | 1044 ± 86 | ^a^ | 934 ± 57 | ^a^ | 1110 ± 66 | ^a^ |
|  | 43 | 33 | 826 ± 58 | ^a^ | 1011 ± 85 | ^a^ | 982 ± 92 | ^a^ | 964 ± 105 | ^a^ |
|  | 63 | 35 | 1036 ± 87 | ^a^ | 1148 ± 101 | ^a^ | 1006 ± 62 | ^a^ | 1030 ± 63 | ^a^ |
|  | **Average** | | **897 ± 33** | **^a^** | **1022 ± 45** | **^a^** | **978 ± 34** | **^a^** | **1033 ± 40** | **^a^** |
