# Supplementary material for: Assessing the potential of seaweed extracts to improve vegetative, physiological and berry quality parameters in Vitis vinifera cv. Chardonnay under cool climatic conditions
Source: PLoS One. 2025 Sep 2;20(9):e0331039. doi: 10.1371/journal.pone.0331039 (PMC12404493; doi:10.1371/journal.pone.0331039)
Supplement: S4 Table — Chardonnay following treatment with water as control, an A. nodosum extract, an E. maxima extract, and an NPK-reference treatment. Each value represents the mean ± standard error of the raw data (n = 12). Within each development stage for each season, treatments that showed significantly different responses are indicated with different letters based on their estimated marginal means (P < 0.05). (DOCX) [file pone.0331039.s008.docx]

S4 Table. Average leaf chlorophyll content index (CCI), reaction centra density per cross section (RC/CS), photosystem II (PSII) maximum quantum yield (F_v_/F_m_), PSII electron transport efficiency (Φ_E0_), and leaf stomatal conductance (σ) of *V. vinifera* cv. Chardonnay following treatment with water as control, an *A. nodosum* extract, an *E. maxima* extract, and an NPK-reference treatment. Each value represents the mean ± standard error of the raw data (*n* = 12). Within each development stage for each season, treatments that showed significantly different responses are indicated with different letters based on their estimated marginal means (*P* < 0.05).

| Year | DAA | E-L stage | Control | | *A. nodosum* | | *E. maxima* | | NPK-Ref | |
| --- | --- | --- | --- | --- | --- | --- | --- | --- | --- | --- |
| **Chlorophyll content index (CCI)** | | | | | | | | | | |
| 2021 | 0 | 23 | 16.6 ± 0.5 | ^a^ | 18.2 ± 0.5 | ^a^ | 16.8 ± 0.4 | ^a^ | 16.7 ± 0.6 | ^a^ |
|  | 8 | 27 | 17.5 ± 0.4 | ^a^ | 18.8 ± 0.6 | ^a^ | 17.7 ± 0.5 | ^a^ | 18.1 ± 0.3 | ^a^ |
|  | 43 | 33 | 15.2 ± 0.4 | ^b^ | 17.8 ± 0.6 | ^a^ | 15.5 ± 0.5 | ^b^ | 16.3 ± 0.5 | ^ab^ |
|  | 57 | 34 | 14.8 ± 0.4 | ^b^ | 16.8 ± 0.6 | ^a^ | 14.5 ± 0.7 | ^b^ | 15.3 ± 0.6 | ^ab^ |
|  | **Average** | | **16.0 ± 0.3** | **^b^** | **17.9 ± 0.3** | **^a^** | **16.1 ± 0.3** | **^b^** | **16.6 ± 0.3** | **^ab^** |
| 2022 | 0 | 23 | 12.0 ± 0.2 | ^a^ | 11.9 ± 0.5 | ^a^ | 12.4 ± 0.5 | ^a^ | 12.0 ± 0.7 | ^a^ |
|  | 6 | 27 | 12.2 ± 0.3 | ^a^ | 13.1 ± 0.5 | ^a^ | 13.0 ± 0.5 | ^a^ | 13.4 ± 0.6 | ^a^ |
|  | 21 | 31 | 12.4 ± 0.3 | ^a^ | 13.4 ± 0.6 | ^a^ | 13.4 ± 0.4 | ^a^ | 13.3 ± 0.6 | ^a^ |
|  | 29 | 32 | 11.1 ± 0.4 | ^a^ | 12.0 ± 0.6 | ^a^ | 12.0 ± 0.4 | ^a^ | 11.8 ± 0.6 | ^a^ |
|  | 35 | 32 | 11.9 ± 0.2 | ^a^ | 11.9 ± 0.2 | ^a^ | 12.0 ± 0.2 | ^a^ | 12.2 ± 0.3 | ^a^ |
|  | 43 | 33 | 11.7 ± 0.4 | ^a^ | 12.4 ± 0.5 | ^a^ | 12.4 ± 0.5 | ^a^ | 12.2 ± 0.7 | ^a^ |
|  | 49 | 34 | 11.0 ± 0.5 | ^a^ | 11.8 ± 0.5 | ^a^ | 11.6 ± 0.5 | ^a^ | 11.6 ± 0.7 | ^a^ |
|  | 63 | 35 | 10.4 ± 0.6 | ^a^ | 11.4 ± 0.5 | ^a^ | 11.3 ± 0.3 | ^a^ | 11.4 ± 0.4 | ^a^ |
|  | **Average** | | **11.6 ± 0.1** | **^a^** | **12.2 ± 0.2** | **^a^** | **12.3 ± 0.2** | **^a^** | **12.2 ± 0.2** | **^a^** |
| **Reaction centra per cross section (RC/CS)** | | | | | | | | | | |
| 2021 | 0 | 23 | 4394 ± 70 | ^b^ | 4669 ± 74 | ^ab^ | 4522 ± 97 | ^b^ | 4810 ± 125 | ^a^ |
|  | 8 | 27 | 4876 ± 91 | ^a^ | 4966 ± 102 | ^b^ | 4840 ± 58 | ^b^ | 4989 ± 163 | ^b^ |
|  | 20 | 31 | 4210 ± 88 | ^a^ | 4532 ± 73 | ^b^ | 4224 ± 66 | ^b^ | 4186 ± 95 | ^b^ |
|  | 29 | 32 | 4198 ± 104 | ^b^ | 4626 ± 110 | ^a^ | 4181 ± 79 | ^b^ | 4300 ± 142 | ^ab^ |
|  | 43 | 33 | 4069 ± 111 | ^a^ | 4274 ± 143 | ^b^ | 4155 ± 126 | ^b^ | 4140 ± 89 | ^b^ |
|  | 57 | 34 | 3900 ± 91 | ^ab^ | 4152 ± 132 | ^a^ | 3556 ± 121 | ^b^ | 4008 ± 106 | ^a^ |
|  | **Average** | | **4275 ± 52** | **^b^** | **4536 ± 53** | **^a^** | **4246 ± 60** | **^b^** | **4406 ± 65** | **^ab^** |
| 2022 | 0 | 23 | 4047 ± 73 | ^a^ | 4144 ± 109 | ^a^ | 4078 ± 146 | ^a^ | 4129 ± 121 | ^a^ |
|  | 6 | 27 | 4933 ± 120 | ^a^ | 4477 ± 88 | ^a^ | 4725 ± 155 | ^a^ | 4794 ± 65 | ^a^ |
|  | 14 | 29 | 4014 ± 69 | ^a^ | 4259 ± 80 | ^a^ | 4255 ± 127 | ^a^ | 4058 ± 84 | ^a^ |
|  | 21 | 31 | 4023 ± 80 | ^a^ | 4253 ± 84 | ^a^ | 4275 ± 119 | ^a^ | 4176 ± 134 | ^a^ |
|  | 29 | 32 | 3901 ± 100 | ^a^ | 4107 ± 90 | ^a^ | 4173 ± 111 | ^a^ | 4023 ± 114 | ^a^ |
|  | 35 | 32 | 4085 ± 123 | ^a^ | 4255 ± 77 | ^a^ | 4429 ± 114 | ^a^ | 4248 ± 149 | ^a^ |
|  | 43 | 33 | 3830 ± 115 | ^a^ | 3901 ± 100 | ^a^ | 4006 ± 116 | ^a^ | 3920 ± 137 | ^a^ |
|  | 49 | 34 | 3315 ± 107 | ^a^ | 3481 ± 71 | ^a^ | 3550 ± 101 | ^a^ | 3508 ± 115 | ^a^ |
|  | 58 | 34 | 3578 ± 157 | ^a^ | 3828 ± 134 | ^a^ | 3692 ± 100 | ^a^ | 3795 ± 170 | ^a^ |
|  | 63 | 35 | 3380 ± 322 | ^a^ | 2795 ± 134 | ^b^ | 3124 ± 195 | ^ab^ | 3009 ± 150 | ^ab^ |
|  | **Average** | | **3911 ± 59** |  | **3950 ± 52** |  | **4031 ± 57** |  | **3966 ± 57** |  |
| **PSII maximum quantum yield (F_v_/F_m_)** | | | | | | | | | | |
| 2021 | 0 | 23 | 0.792 ± 0.004 | ^a^ | 0.788 ± 0.008 | ^a^ | 0.783 ± 0.007 | ^a^ | 0.786 ± 0.007 | ^a^ |
|  | 8 | 27 | 0.783 ± 0.006 | ^a^ | 0.793 ± 0.006 | ^a^ | 0.777 ± 0.005 | ^a^ | 0.777 ± 0.005 | ^a^ |
|  | 20 | 31 | 0.763 ± 0.005 | ^a^ | 0.777 ± 0.007 | ^a^ | 0.764 ± 0.007 | ^a^ | 0.755 ± 0.004 | ^a^ |
|  | 29 | 32 | 0.768 ± 0.010 | ^ab^ | 0.785 ± 0.006 | ^a^ | 0.772 ± 0.007 | ^ab^ | 0.760 ± 0.007 | ^b^ |
|  | 43 | 33 | 0.774 ± 0.004 | ^a^ | 0.772 ± 0.005 | ^a^ | 0.771 ± 0.005 | ^a^ | 0.771 ± 0.004 | ^a^ |
|  | 57 | 34 | 0.752 ± 0.009 | ^a^ | 0.748 ± 0.010 | ^a^ | 0.749 ± 0.010 | ^a^ | 0.746 ± 0.009 | ^a^ |
|  | **Average** | | **0.772 ± 0.003** | **^ab^** | **0.777 ± 0.003** | **^a^** | **0.770 ± 0.003** | **^ab^** | **0.766 ± 0.003** | **^b^** |
| 2022 | 0 | 23 | 0.776 ± 0.004 | ^a^ | 0.786 ± 0.004 | ^a^ | 0.774 ± 0.004 | ^a^ | 0.785 ± 0.004 | ^a^ |
|  | 6 | 27 | 0.786 ± 0.005 | ^a^ | 0.777 ± 0.003 | ^a^ | 0.781 ± 0.005 | ^a^ | 0.781 ± 0.005 | ^a^ |
|  | 14 | 29 | 0.781 ± 0.004 | ^a^ | 0.794 ± 0.007 | ^a^ | 0.786 ± 0.007 | ^a^ | 0.781 ± 0.007 | ^a^ |
|  | 21 | 31 | 0.773 ± 0.004 | ^a^ | 0.784 ± 0.004 | ^a^ | 0.777 ± 0.005 | ^a^ | 0.779 ± 0.004 | ^a^ |
|  | 29 | 32 | 0.791 ± 0.005 | ^a^ | 0.800 ± 0.004 | ^a^ | 0.799 ± 0.005 | ^a^ | 0.799 ± 0.003 | ^a^ |
|  | 35 | 32 | 0.821 ± 0.003 | ^a^ | 0.822 ± 0.002 | ^a^ | 0.822 ± 0.001 | ^a^ | 0.821 ± 0.002 | ^a^ |
|  | 43 | 33 | 0.773 ± 0.007 | ^a^ | 0.778 ± 0.004 | ^a^ | 0.781 ± 0.006 | ^a^ | 0.783 ± 0.003 | ^a^ |
|  | 49 | 34 | 0.762 ± 0.008 | ^b^ | 0.773 ± 0.006 | ^ab^ | 0.785 ± 0.005 | ^a^ | 0.782 ± 0.005 | ^a^ |
|  | 58 | 34 | 0.795 ± 0.005 | ^a^ | 0.804 ± 0.005 | ^a^ | 0.802 ± 0.004 | ^a^ | 0.809 ± 0.004 | ^a^ |
|  | 63 | 35 | 0.818 ± 0.002 | ^a^ | 0.816 ± 0.002 | ^a^ | 0.816 ± 0.002 | ^a^ | 0.816 ± 0.003 | ^a^ |
|  | **Average** | | **0.788 ± 0.002** | **^a^** | **0.793 ± 0.002** | **^a^** | **0.792 ± 0.002** | **^a^** | **0.794 ± 0.002** | **^a^** |
| **PSII electron transport efficiency (Φ_E0_)** | | | | | | | | | | |
|  | 0 | 23 | 0.380 ± 0.019 | ^a^ | 0.368 ± 0.018 | ^a^ | 0.348 ± 0.017 | ^a^ | 0.345 ± 0.013 | ^a^ |
|  | 8 | 27 | 0.307 ± 0.022 | ^a^ | 0.321 ± 0.012 | ^a^ | 0.284 ± 0.009 | ^a^ | 0.303 ± 0.011 | ^a^ |
|  | 20 | 31 | 0.272 ± 0.012 | ^a^ | 0.305 ± 0.012 | ^a^ | 0.312 ± 0.025 | ^a^ | 0.282 ± 0.022 | ^a^ |
|  | 29 | 32 | 0.317 ± 0.019 | ^a^ | 0.357 ± 0.014 | ^a^ | 0.330 ± 0.018 | ^a^ | 0.304 ± 0.017 | ^a^ |
|  | 43 | 33 | 0.309 ± 0.012 | ^a^ | 0.306 ± 0.017 | ^a^ | 0.303 ± 0.014 | ^a^ | 0.311 ± 0.011 | ^a^ |
|  | 57 | 34 | 0.275 ± 0.015 | ^a^ | 0.299 ± 0.020 | ^a^ | 0.270 ± 0.018 | ^a^ | 0.282 ± 0.019 | ^a^ |
|  | **Average** | | **0.310 ± 0.008** | **^a^** | **0.326 ± 0.007** | **^a^** | **0.308 ± 0.008** | **^a^** | **0.305 ± 0.007** | **^a^** |
|  | 0 | 23 | 0.316 ± 0.010 | ^a^ | 0.329 ± 0.010 | ^a^ | 0.323 ± 0.011 | ^a^ | 0.328 ± 0.010 | ^a^ |
|  | 6 | 27 | 0.358 ± 0.014 | ^a^ | 0.320 ± 0.012 | ^a^ | 0.342 ± 0.011 | ^a^ | 0.342 ± 0.010 | ^a^ |
|  | 14 | 29 | 0.291 ± 0.015 | ^a^ | 0.318 ± 0.013 | ^a^ | 0.310 ± 0.016 | ^a^ | 0.315 ± 0.009 | ^a^ |
|  | 21 | 31 | 0.302 ± 0.011 | ^a^ | 0.331 ± 0.009 | ^a^ | 0.321 ± 0.010 | ^a^ | 0.316 ± 0.010 | ^a^ |
|  | 29 | 32 | 0.366 ± 0.012 | ^a^ | 0.381 ± 0.012 | ^a^ | 0.388 ± 0.011 | ^a^ | 0.379 ± 0.009 | ^a^ |
|  | 35 | 32 | 0.400 ± 0.007 | ^a^ | 0.409 ± 0.010 | ^a^ | 0.407 ± 0.011 | ^a^ | 0.400 ± 0.008 | ^a^ |
|  | 43 | 33 | 0.313 ± 0.011 | ^a^ | 0.340 ± 0.010 | ^a^ | 0.334 ± 0.016 | ^a^ | 0.343 ± 0.011 | ^a^ |
|  | 49 | 34 | 0.294 ± 0.014 | ^a^ | 0.290 ± 0.011 | ^a^ | 0.325 ± 0.016 | ^a^ | 0.319 ± 0.011 | ^a^ |
|  | 58 | 34 | 0.352 ± 0.014 | ^b^ | 0.398 ± 0.012 | ^a^ | 0.375 ± 0.016 | ^ab^ | 0.380 ± 0.008 | ^ab^ |
|  | 63 | 35 | 0.414 ± 0.015 | ^ab^ | 0.390 ± 0.015 | ^b^ | 0.399 ± 0.005 | ^b^ | 0.444 ± 0.016 | ^a^ |
|  | **Average** | | **0.341 ± 0.005** | **^a^** | **0.351 ± 0.005** | **^a^** | **0.352 ± 0.005** | **^a^** | **0.356 ± 0.005** | **^a^** |
| **Stomatal conductance (mmol m^−2^ s^−1^)** | | | | | | | | | | |
| 2021 | 5 | 25 | 367 ± 28 | ^a^ | 383 ± 24 | ^a^ | 374 ± 43 | ^a^ | 329 ± 34 | ^a^ |
|  | 20 | 31 | 455 ± 24 | ^a^ | 486 ± 12 | ^a^ | 489 ± 19 | ^a^ | 462 ± 18 | ^a^ |
|  | 29 | 32 | 325 ± 22 | ^a^ | 283 ± 14 | ^a^ | 289 ± 19 | ^a^ | 294 ± 17 | ^a^ |
|  | 43 | 33 | 385 ± 14 | ^a^ | 382 ± 15 | ^a^ | 375 ± 9 | ^a^ | 409 ± 17 | ^a^ |
|  | **Average** | | **383 ± 13** | **^a^** | **383 ± 13** | **^a^** | **382 ± 16** | **^a^** | **374 ± 15** | **^a^** |
| 2022 | 2 | 25 | 251 ± 14 | ^a^ | 254 ± 20 | ^a^ | 237 ± 16 | ^a^ | 238 ± 15 | ^a^ |
|  | 14 | 29 | 289 ± 22 | ^a^ | 295 ± 14 | ^a^ | 274 ± 26 | ^a^ | 280 ± 23 | ^a^ |
|  | 21 | 31 | 241 ± 20 | ^a^ | 212 ± 12 | ^a^ | 195 ± 14 | ^a^ | 222 ± 11 | ^a^ |
|  | 29 | 32 | 284 ± 23 | ^a^ | 300 ± 24 | ^a^ | 279 ± 19 | ^a^ | 262 ± 15 | ^a^ |
|  | 43 | 33 | 271 ± 18 | ^a^ | 287 ± 26 | ^a^ | 280 ± 19 | ^a^ | 274 ± 20 | ^a^ |
|  | 49 | 34 | 242 ± 17 | ^a^ | 248 ± 17 | ^a^ | 260 ± 22 | ^a^ | 244 ± 23 | ^a^ |
|  | **Average** | | **263 ± 8** | **^a^** | **266 ± 9** | **^a^** | **254 ± 9** | **^a^** | **253 ± 8** | **^a^** |
