# Supplementary material for: Assessing the potential of seaweed extracts to improve vegetative, physiological and berry quality parameters in Vitis vinifera cv. Chardonnay under cool climatic conditions
Source: PLoS One. 2025 Sep 2;20(9):e0331039. doi: 10.1371/journal.pone.0331039 (PMC12404493; doi:10.1371/journal.pone.0331039)
Supplement: S5 Table — (DOCX) [file pone.0331039.s009.docx]

S5 Table. Effects of treatment, development stage (time), season (2021 vs 2022), their interactions and covariates VPD and Rain on berry morphology and classical parameters of *V. vinifera* cv. Chardonnay.

| Response variable | | Season | | Treatment | | Time | | Season x Treatment | | Season x Time | | Treatment x Time | | VPD | | Rain | | |
| --- | --- | --- | --- | --- | --- | --- | --- | --- | --- | --- | --- | --- | --- | --- | --- | --- | --- | --- |
|  | **Berry anatomical measurements** | | | | | | | | | | | | | | | | |  |
|  | | **F-val** | ***P*-val** | **F-val** | ***P*-val** | **F-val** | ***P*-val** | **F-val** | ***P*-val** | **F-val** | ***P*-val** | **F-val** | ***P*-val** | **F-val** | ***P*-val** | **F-val** | ***P*-val** | |
| Berry volume (mL) | | 10.28 | *0.002^**^* | 1.867 | *0.189* | 96.65 | *<.001^***^* | X |  | 2.571 | *0.081* | X |  | X |  | X |  | |
| Berry mass (g) | | 12.83 | *<.001^***^* | 2.022 | *0.165* | 129.2 | *<.001^***^* | X |  | 1.174 | *0.312* | X |  | X |  | X |  | |
| Berry density (g/L) | | 7.289 | *0.008^**^* | 0.101 | *0.954* | 4.836 | *0.010^**^* | X |  | 19.13 | *<.001^***^* | X |  | 12.78 | *<.001^***^* | X |  | |
| Berry dry matter (%) | | 103.6 | *<.001^***^* | 0.200 | *0.895* | 1285 | *<.001^***^* | X |  | 35.62 | *<.001^***^* | X |  | 46.22 | *<.001^***^* | X |  | |
|  | **Berry classical parameters** | | | | | | | | | | | | | | | | |  |
| Titratable acidity TA (g/mL) | | 234.4 | *<.001^***^* | 1.416 | *0.285* | 349.4 | *<.001^***^* | 0.258 | *0.856* | 8.437 | *<.001^***^* | X |  | X |  | 30.54 | *<.001^***^* | |
| pH | | 62.39 | *<.001^***^* | 1.387 | *0.294* | 131.9 | *<.001^***^* | X |  | X |  | X |  | 66.58 | *<.001^***^* | 18.72 | *<.001^***^* | |
| Total soluble solids TSS (°Brix) | | 429.8 | *<.001^***^* | 0.676 | *0.583* | 386.2 | *<.001^***^* | X |  | 28.57 | *<.001^***^* | X |  | X |  | 21.72 | *<.001^***^* | |
| Berry sugar content (g/berry) | | 130.2 | *<.001^***^* | 2.065 | *0.158* | 518.6 | *<.001^***^* | 0.090 | *0.965* | 16.93 | *<.001^***^* | X |  | 5.256 | *0.024^*^* | X |  | |

F-statistic values from ANOVA on four panels per treatment measured over the course of 7 weeks with measurements done every 10 to 14 (Linear Mixed Model). Asterisks indicate significant differences between the treatments (0.01 < *P* ≤ 0.05: * ; 0.001 < *P* ≤ 0.01: ** ; *P* ≤ 0.001: ***).

Parameters marked with X were excluded from the final model based on the lowest AIC.
