# Supplementary material for: Assessing the potential of seaweed extracts to improve vegetative, physiological and berry quality parameters in Vitis vinifera cv. Chardonnay under cool climatic conditions
Source: PLoS One. 2025 Sep 2;20(9):e0331039. doi: 10.1371/journal.pone.0331039 (PMC12404493; doi:10.1371/journal.pone.0331039)
Supplement: S6 Table — Chardonnay in response to treatment with water as control, an A. nodosum extract, an E. maxima extract, and an NPK-reference treatment. Each value represents the mean ± standard error of the raw data (n = 4). Treatments that showed significantly different responses, averaged over the ripening period and at harvest, are indicated with different letters based on their estimated marginal means (P < 0.05). (DOCX) [file pone.0331039.s010.docx]

S6 Table. Average berry volume and berry mass of *V. vinifera* cv. Chardonnay in response to treatment with water as control, an *A. nodosum* extract, an *E. maxima* extract, and an NPK-reference treatment. Each value represents the mean ± standard error of the raw data (*n* = 4). Treatments that showed significantly different responses, averaged over the ripening period and at harvest, are indicated with different letters based on their estimated marginal means (*P* < 0.05).

| Year | DAA | E-L stage | Control | | *A. nodosum* | | *E. maxima* | | NPK-Ref | |
| --- | --- | --- | --- | --- | --- | --- | --- | --- | --- | --- |
| **Berry volume (mL)** | | | | | | | | | | |
| 2021 | 72 | 35 | 1.25 ± 0.04 |  | 1.44 ± 0.02 |  | 1.38 ± 0.03 |  | 1.35 ± 0.05 |  |
|  | 93 | 36 | 1.62 ± 0.07 |  | 1.70 ± 0.04 |  | 1.63 ± 0.06 |  | 1.71 ± 0.10 |  |
|  | 106 | 37 | 1.64 ± 0.12 |  | 1.65 ± 0.07 |  | 1.61 ± 0.06 |  | 1.71 ± 0.05 |  |
|  | **120** | **38** | **1.45 ± 0.03** | **^a^** | **1.55 ± 0.03** | **^a^** | **1.51 ± 0.08** | **^a^** | **1.57 ± 0.04** | **^a^** |
|  | **Average** | | **1.49 ± 0.05** | **^a^** | **1.58 ± 0.03** | **^a^** | **1.53 ± 0.04** | **^a^** | **1.58 ± 0.05** | **^a^** |
| 2022 | 63 | 35 | 1.18 ± 0.03 |  | 1.27 ± 0.04 |  | 1.35 ± 0.05 |  | 1.31 ± 0.06 |  |
|  | 71 | 36 | 1.38 ± 0.08 |  | 1.47 ± 0.02 |  | 1.42 ± 0.10 |  | 1.38 ± 0.13 |  |
|  | 82 | 37 | 1.49 ± 0.03 |  | 1.59 ± 0.07 |  | 1.53 ± 0.02 |  | 1.65 ± 0.09 |  |
|  | 99 | 37 | 1.59 ± 0.06 |  | 1.74 ± 0.12 |  | 1.69 ± 0.07 |  | 1.74 ± 0.06 |  |
|  | **114** | **38** | **1.59 ± 0.08** | **^a^** | **1.73 ± 0.06** | **^a^** | **1.68 ± 0.05** | **^a^** | **1.69 ± 0.07** | **^a^** |
|  | **Average** | | **1.44 ± 0.04** | **^b^** | **1.56 ± 0.05** | **^a^** | **1.54 ± 0.04** | **^ab^** | **1.55 ± 0.05** | **^a^** |
| **Berry mass (g)** | | | | | | | | | | |
| 2021 | 72 | 35 | 1.40 ± 0.02 |  | 1.54 ± 0.01 |  | 1.51 ± 0.02 |  | 1.50 ± 0.05 |  |
|  | 93 | 36 | 1.80 ± 0.07 |  | 1.86 ± 0.05 |  | 1.83 ± 0.04 |  | 1.88 ± 0.11 |  |
|  | 106 | 37 | 1.82 ± 0.11 |  | 1.88 ± 0.04 |  | 1.81 ± 0.04 |  | 1.90 ± 0.06 |  |
|  | **120** | **38** | **1.68 ± 0.06** | **^a^** | **1.77 ± 0.02** | **^a^** | **1.75 ± 0.09** | **^a^** | **1.80 ± 0.03** | **^a^** |
|  | **Average** | | **1.68 ± 0.05** | **^a^** | **1.76 ± 0.04** | **^a^** | **1.72 ± 0.04** | **^a^** | **1.77 ± 0.05** | **^a^** |
| 2022 | 63 | 35 | 1.31 ± 0.04 |  | 1.40 ± 0.04 |  | 1.46 ± 0.07 |  | 1.43 ± 0.07 |  |
|  | 71 | 36 | 1.50 ± 0.09 |  | 1.63 ± 0.02 |  | 1.56 ± 0.10 |  | 1.55 ± 0.15 |  |
|  | 82 | 37 | 1.65 ± 0.03 |  | 1.77 ± 0.08 |  | 1.72 ± 0.04 |  | 1.83 ± 0.10 |  |
|  | 99 | 37 | 1.83 ± 0.07 |  | 2.02 ± 0.10 |  | 1.97 ± 0.07 |  | 2.03 ± 0.06 |  |
|  | **114** | **38** | **1.76 ± 0.08** | **^a^** | **1.90 ± 0.10** | **^a^** | **1.81 ± 0.05** | **^a^** | **1.87 ± 0.06** | **^a^** |
|  | **Average** | | **1.61 ± 0.05** | **^b^** | **1.75 ± 0.06** | **^a^** | **1.70 ± 0.05** | **^ab^** | **1.74 ± 0.06** | **^a^** |
