# Supplementary material for: Assessing the potential of seaweed extracts to improve vegetative, physiological and berry quality parameters in Vitis vinifera cv. Chardonnay under cool climatic conditions
Source: PLoS One. 2025 Sep 2;20(9):e0331039. doi: 10.1371/journal.pone.0331039 (PMC12404493; doi:10.1371/journal.pone.0331039)
Supplement: S7 Table — Chardonnay in response to treatment with water as control, an A. nodosum extract, an E. maxima extract, and an NPK-reference treatment. Each value represents the mean ± standard error of the raw data (n = 4). Treatments that showed significantly different responses, averaged over the ripening period and at harvest, are indicated with different letters based on their estimated marginal means (P < 0.05). (DOCX) [file pone.0331039.s011.docx]

S7 Table. Average titratable acidity, total soluble solids (°Brix) and sugar content per berry of *V. vinifera* cv. Chardonnay in response to treatment with water as control, an *A. nodosum* extract, an *E. maxima* extract, and an NPK-reference treatment. Each value represents the mean ± standard error of the raw data (*n* = 4). Treatments that showed significantly different responses, averaged over the ripening period and at harvest, are indicated with different letters based on their estimated marginal means (*P* < 0.05).

| Year | DAA | E-L stage | Control | | *A. nodosum* | | *E. maxima* | | NPK-Ref | |
| --- | --- | --- | --- | --- | --- | --- | --- | --- | --- | --- |
| **Titratable acidity (g L^-1^)** | | | | | | | | | | |
| 2021 | 72 | 35 | 22.8 ± 0.4 |  | 22.6 ± 0.8 |  | 22.0 ± 0.4 |  | 22.2 ± 0.7 |  |
|  | 93 | 36 | 12.5 ± 0.4 |  | 13.4 ± 0.7 |  | 12.3 ± 0.7 |  | 13.3 ± 0.6 |  |
|  | 106 | 37 | 11.3 ± 0.6 |  | 11.5 ± 0.5 |  | 11.1 ± 0.5 |  | 11.9 ± 0.6 |  |
|  | **120** | **38** | **10.2 ± 0.2** | **^a^** | **10.4 ± 0.1** | **^a^** | **10.4 ± 0.2** | **^a^** | **10.3 ± 0.2** | **^a^** |
|  | **Average** | | **14.2 ± 1.3** | **^a^** | **14.5 ± 1.3** | **^a^** | **14.0 ± 1.2** | **^a^** | **14.4 ± 1.2** | **^a^** |
| 2022 | 63 | 35 | 24.4 ± 1.3 |  | 24.0 ± 1.5 |  | 23.8 ± 0.2 |  | 24.8 ± 1.8 |  |
|  | 71 | 36 | 17.1 ± 0.6 |  | 18.9 ± 1.9 |  | 18.1 ± 0.5 |  | 17.7 ± 0.6 |  |
|  | 82 | 37 | 10.7 ± 0.3 |  | 12.0 ± 0.4 |  | 11.1 ± 0.4 |  | 11.4 ± 0.6 |  |
|  | 99 | 37 | 9.3 ± 0.2 |  | 9.7 ± 0.2 |  | 9.5 ± 0.2 |  | 9.5 ± 0.2 |  |
|  | **114** | **38** | **7.2 ± 0.4** | **^a^** | **8.3 ± 1.0** | **^a^** | **7.4 ± 0.3** | **^a^** | **7.8 ± 0.3** | **^a^** |
|  | **Average** | | **13.7 ± 1.5** | **^a^** | **14.6 ± 1.4** | **^a^** | **14.0 ± 1.4** | **^a^** | **14.2 ± 1.5** | **^a^** |
| **Total Soluble Solids (°Brix)** | | | | | | | | | | |
| 2021 | 72 | 35 | 9.9 ± 0.2 |  | 10.8 ± 0.5 |  | 10.5 ± 0.6 |  | 10.6 ± 0.8 |  |
|  | 93 | 36 | 15.2 ± 0.4 |  | 16.6 ± 0.4 |  | 15.8 ± 0.5 |  | 16.2 ± 0.8 |  |
|  | 106 | 37 | 17.5 ± 0.4 |  | 18.5 ± 0.3 |  | 17.9 ± 0.3 |  | 18.0 ± 0.9 |  |
|  | **120** | **38** | **20.5 ± 0.1** | **^a^** | **20.4 ± 0.3** | **^a^** | **20.8 ± 0.4** | **^a^** | **21.2 ± 0.6** | **^a^** |
|  | **Average** | | **15.8 ± 1.0** | **^b^** | **16.6 ± 0.9** | **^a^** | **16.3 ± 1.0** | **^ab^** | **16.5 ± 1.1** | **^ab^** |
| 2022 | 63 | 35 | 10.2 ± 0.5 |  | 10.4 ± 0.8 |  | 10.8 ± 0.4 |  | 10.8 ± 0.9 |  |
|  | 71 | 36 | 13.3 ± 0.3 |  | 13.9 ± 0.1 |  | 13.1 ± 0.6 |  | 13.9 ± 0.3 |  |
|  | 82 | 37 | 18.3 ± 0.2 |  | 18.3 ± 0.2 |  | 18.1 ± 0.4 |  | 18.8 ± 0.2 |  |
|  | 99 | 37 | 19.9 ± 0.1 |  | 19.9 ± 0.2 |  | 19.5 ± 0.3 |  | 20.3 ± 0.2 |  |
|  | **114** | **38** | **21.2 ± 0.2** | **^a^** | **20.7 ± 0.6** | **^a^** | **21.0 ± 0.2** | **^a^** | **21.3 ± 0.3** | **^a^** |
|  | **Average** | | **16.6 ± 1.0** | **^a^** | **16.7 ± 0.9** | **^a^** | **16.5 ± 0.9** | **^a^** | **17.0 ± 0.9** | **^a^** |
| **Sugar content (mg berry^-1^)** | | | | | | | | | | |
| 2021 | 72 | 35 | 139 ± 3 |  | 167 ± 8 |  | 158 ± 10 |  | 160 ± 16 |  |
|  | 93 | 36 | 275 ± 13 |  | 311 ± 16 |  | 291 ± 13 |  | 306 ± 26 |  |
|  | 106 | 37 | 318 ± 16 |  | 350 ± 3 |  | 325 ± 2 |  | 343 ± 27 |  |
|  | **120** | **38** | **347 ± 14** | **^a^** | **361 ± 6** | **^a^** | **366 ± 24** | **^a^** | **383 ± 17** | **^a^** |
|  | **Average** | | **270 ± 21** | **^a^** | **297 ± 21** | **^a^** | **285 ± 21** | **^a^** | **298 ± 24** | **^a^** |
| 2022 | 63 | 35 | 135 ± 7 |  | 146 ± 13 |  | 159 ± 12 |  | 156 ± 20 |  |
|  | 71 | 36 | 201 ± 15 |  | 227 ± 5 |  | 204 ± 15 |  | 217 ± 23 |  |
|  | 82 | 37 | 303 ± 3 |  | 326 ± 14 |  | 313 ± 14 |  | 345 ± 21 |  |
|  | 99 | 37 | 367 ± 13 |  | 405 ± 20 |  | 383 ± 9 |  | 414 ± 14 |  |
|  | **114** | **38** | **373 ± 16** | **^a^** | **396 ± 25** | **^a^** | **382 ± 13** | **^a^** | **400 ± 13** | **^a^** |
|  | **Average** | | **276 ± 22** | **^b^** | **300 ± 24** | **^a^** | **288 ± 22** | **^ab^** | **306 ± 25** | **^a^** |
