# Supplementary material for: Assessing the potential of seaweed extracts to improve vegetative, physiological and berry quality parameters in Vitis vinifera cv. Chardonnay under cool climatic conditions
Source: PLoS One. 2025 Sep 2;20(9):e0331039. doi: 10.1371/journal.pone.0331039 (PMC12404493; doi:10.1371/journal.pone.0331039)
Supplement: Attachment — Submitted filename: Comments on the Manuscript PONE-D-24-45452.pdf [file pone.0331039.s013.pdf]

Comments on the Manuscript

Title: Assessing the potential of seaweed extracts to improve vegetative, physiological and reproductive characteristics in *Vitis vinifera* cv. Chardonnay under cool climatic conditions
Manuscript ID: PONE-D-24-45452
Journal: PLOS ONE

Mail goal – to study the impact of foliar application of extracts from two algae species – *Ascophyllum nodosum* and *Ecklonia maxima* - as biostimulants on the performance of Chardonnay cultivar under cool-climate conditions, as those find in Belgium (where the experimental site is), as an opportunity to explore sustainable viticultural bio-based practices in new emerging/expanding vineyard regions.
Both brown algae (kelp) abundant in cold waters - *A. nodosum* from the North Sea to the north-occidental European coast and *E. maxima* mostly in the southern Atlantic coast of Africa, from the south of South Africa to Namibia – and already used for food and feed and as agricultural supplement.

General comments:

Globally this work does not constitute cutting edge research but:

- It focusses on an interesting and pertinent issue – viticultural practices based on natural sustainable inputs to improve yield and quality, proposing the use of marine algal extracts.
- It is very well written; the Introduction is well supported and M&M are thoroughly described and the field trials carefully planned and conducted.
- The amount and quality of the results are very good.
- It has a good discussion section.

However,

- The Introduction should be much more informative in some aspects, such as giving evidence/refs regarding macro- and micronutrients/extract components uptake by the leaves (as they test the treatments by foliar application); also, attention with the harvesting of seaweed material was not approach and is important, specially knowing that one of the species (*E. maxima*) is now under pressure due to CC.
- The Material and Methods section should provide more detailed information here and there (see more in Specific comments).
- Treatment effects are meagre but that is not a problem, however, the Results should be better described/discussed: highlighting differences in comparison with the "fair" reference (NPK-ref.) and not so much the Control (without any fertilization), but also acknowledging that similarities are also good news if a substitution of the traditional fertilization is envisioned.
- It has a good Discussion section but should be improved, specially the "Future research" part (see more in Specific comments).

Several specific comments/recommendations can be found below.

For all this I think that in the present form the manuscript is not yet adequate for publication but I'm sure that after the corrections/adaptations/improvements suggested it would be a very good, interesting and honest work, that would contribute with knowledge and new insights to the field.

Specific comments (section by section):

Title (full and short versions)
ok

Keywords: of the 6 words/expressions included, 2 (seaweed extracts and *Vitis vinifera*) are already present in the title so they could be replaced by other relevant key words/expressions (like new emerging/expanding vineyard sites). Also, the authors didn't assess "Photosystems' efficiency", only of PSII, so I would suggest to replace by "Maximum quantum yield of PSII".

Abstract
- Line 26 – total "leaf area" of the canopy or of the area of a leaf? – clarify.
- Line 26 – "chlorophyll content"– include units.
- Lines 27-28 – regarding "PSII quantum yield": is it "effective" or "maximum" quantum yield? Those are very different things – complete and use the same designation throughout the manuscript (ms) for consistency.
- Line 28 – regarding "These improvements lead xxx": how do the authors know that this is a cause-effect relationship or the only/main cause of the variation in berry size, mass, etc.? Avoid speculations – rephrase.
- Lines 32-34 – be specific about the parameters; avoid generalizations that may incur in incorrections.
- Line 34 – Clarify what do you mean by "effect sizes".

Introduction
- Line 79 – "have shown promise in grapevines and other xx" – it would be important to indicate some variables/ "numbers" to support this and illustrate que impact of seaweed extracts that is claimed.
- Lines 81-83 – regarding this difference in the number of studies between warmer/drought sites *vs* cooler/more humid sites, and to add pertinence to the approach/idea, the authors should indicate what may be the main limitations in these latter regions and in what way seaweed extracts may help to overcome.
- Line 87 – the hyphen is not needed here because you don't have another last word with one.
- Line 94 – regarding "several winegrape cultivars" – the type of climate/regions where the studies were made and mode of applications should be referred.
- Regarding the use of *E. maxima* – how do the authors picture the harvest and potential wide use of this algae as, currently, the populations in South Africa are at higher risk precisely due do climate changes in water temperature.

- Lines 99-100 – how can the authors evaluate this if no parallel/similar study was done in a warmer site (maintaining as much as possible all the other non-climatic factors) and the literature is so scarce with respect to studies in cooler sites? – rephrase.
- Line 103 – correct typo (cut the hyphen). Also, what does "grape ripening dynamics" mean exactly - specify.

Materials and Methods

- Experimental design
  - Line 124 – correct typo (without hyphen).

- Vine vegetative characteristics
  - "Specific leaf area" appears in S2 Table but the procedure to calculate this parameter was not referred in this section – include.
  - Lines 172-174 – indicate the time interval since the authors will assess starch content.
  - Line 181 – Biological replicates? n=12 or n=4/combined by block? – specify here.
  - Line 188 – use a comma to separate tens or hundreds of thousands, like in 12,500 – apply throughout the ms.
  - Line 196 – don't use a point for the operation multiplication in combined units when using the scientific system, use just a space like: 4.5 m s^{-1} or 3500 µmol m^{-2} s^{-1} - apply throughout the ms.

- Chlorophyll indices and stomatal conductance
  - Line 214 – indicate how CCI is calculated and what does this index translates (correlates with total chlorophyll content per area? per mass?) – it is important to interpret the results.
  - Line 218 – the same applies to Fv/Fm.
  - Line 222 – regarding "to reach the maximum Fv/Fm value" – what does this mean? Fv/Fm is already a parameter that gives the maximum quantum efficiency, supposedly the one and only.

- Berry classical parameters and morphology
  - Line 231 – reformulate the phrase to "Berries were sampled from veráison (XX) until harvest (XX) every xxxx).
  - Line 239 – "berries were crushed"- how, exactly? specify.
  - Line 253 – "The berry volume was estimated from length and diameter" - how, exactly? specify.

- Statistical analysis
  - Line 278 – the abbreviation "AVOVA" should be here and used in line 285.
  - Line 282 – replace "equality" for "homogeneity".

Results

- Weather data

- Line 302 – "from April to June" or "in April and June"?

- Seaweed extract effect on grapevine vegetative growth and physiology
  - Line 315 – replace "At that time" by "On that season".
  - Line 319 – "longer shoot lengths" – reformulate.
  - Line 324 – remove the point in (S2 Table.), it is not needed and the authors didn't use it in, e.g., (Table1) - apply throughout the ms.
  - Lines 333-336 – on line 333, referred to "leaf area", it should be (Fig 2C and D) and on line 336, referred to leaf dry mass, should be (Fig 2A and B).
  - Line 355 – It's "<u>maximum</u> quantum yield of photosystem II" – avoid inconsistencies, the authors use different designations for the parameter Fv/Fm across the ms – revise.
  - Lines 359-360 - "Treatment" was not associated with differences in stomatal conductance as can be seen in S2 Table and Table 1, only "Time" and "Season" and their interaction gave significant effects (S2 Table) – revise.
  - Lines 376-383 – To better follow this description authors should highlight these particular time-points on S4 Table. Also, they should include a column with the days 2021(before that of the developmental stages) to better compare with Figure 3.
  - Regarding Figure 3 – CCI are very different between years and should be addressed, but specially the decay in Fv/Fm in 2021, the least harsh year, should also be addressed.

- Seaweed extract effect on reproductive parameters
  - Line 390 – About "reproductive" – authors should reconsider designation, maybe fruit? berry? as reproductive also implies date of flowering, flowers, etc.
  - Lines 391-392 – On S5 Table, "strong main effects" can only be seen on those 4 berry parameters with "Time"; "Season" as "Treatment" only had significant effects on "berry volume" and "berry mass" – reformulate.
  - Line 401 – regarding "averaged over the ripening period" – more importantly than the average are the berry "metrics" at harvest, better related with fruit yield and quality, don't you think? By Figure 4 there are no differences on those two variables in the last timepoint (and for some reason you depict the last timepoint here and not in Figure 2) – defend your ideas and explain better.
  - Lines 405-407 – shorten the phrase as: "xxx treated with *A. nodosum* <u>and</u> NPK-ref were on average xxxx" – apply to similar situations in order to condense the ms, ease the reading, and to better retain the essentials.
  - Lines 399-408 – regarding comparisons with the Control (and this also applies to similar situations) – the only "fair" comparisons are those of the treatments (extracts) with the NPK-ref and that should be addressed without fear, there is a lot of room to improve this approach leaving that for Discussion or Future pespectives. Clearly, for most of the parameters there are no significant differences comparing to the NPK-ref, but there are some and those should be highlighted.
  - Line 439 – indicate Figure or Table in the end of this statement.

- Seaweed extract effect on harvest parameters
  - Line 466 – correct typo (cut the point)

Discussion:

- Seaweed extracts show environment dependent improvements in leaf anatomy and vine physiological parameters
  - Lines 490-491 – a hyphen is needed in "environment-dependent".
  - Line 492 – Better start with "As for other plants, xx" – foliar photosynthesis is crucial for all the plants, even if other organs could be photosynthetic, like aerial roots, petals, sepals, fruits, etc.
  - Lines 493-494 – If "specific leaf area" is an important parameter it should be better "treated" in M&M and Results. As I can recall it only appears once in Results and no effects were registered in SLA – avoid referring to results/parameters/factors of less or no importance to emphasize the most relevant parts of your work.
  - Lines 494-496 – this is an example where the authors should emphasize the differences found with *A. nodosum* comparing to NPK-ref to highlight the relevance of the extract if a substitution in the type of fertilization is envisaged (apply to other analogous situations).
  - Lines 499 – Take note that Fv/Fm is not the best parameter to assess plant stress (it's much less susceptible than other parameters as the effective quantum yield (Φ_{PSII}), for instance), and it doesn't correlate to "photosynthetic capacity" as Φ_{PSII} (see, e.g, Genty et al. 1989 - BBA, 900, 87-92), so it would be better to replace by "Photosynthetic potential" or "Photosynthetic performance" – this can be used here to explain the poor impact on Fv/Fm by the treatments tested. Also, a precise information of what CCI indicates should be on M&M and Results, and recall it here to better understand the impact of the increase in CCI with *A. nodosum*.
  - Lines 502-503 – Figure 2 says otherwise, there was more vegetative growth on 2021… - reformulate
  - Line 508 - correct typo (separate the units)
  - Line 511 – Better to use RuBisCO
  - Line 514 – Better to use "drier", the authors don't have a measure of water stress or even of water status either of soil or grapevines by the measurement of pre-dawn and/or midday water potentials. In this sense, maybe it should be added that, probably, not even water stress conditions were installed, supporting the no effect on stomatal conductance (together with the anisohydric characteristic of the cultivar).
  - Line 521 – Definitely one thing to "optimize" is the type and regime of application of the extracts (frequency, concentration, foliar vs by irrigation), as well as to monitor the possible lixiviation of the extracts from the leaves by the rain – the authors should address these aspects because it may explain the lack of differences comparing to NPK-ref.

- Berry morphology and classical parameters are positively impacted by seaweed extracts in the warmer second season.

  - Lines 524-525 – add that this stands for the seasonal average and not just to the last time-point (apply to analogous situations).
  - Lines 527-529 – There was a smaller leaf area and leaf mass in 2022 (see Fig. 2) – rephrase and avoid speculation.
  - Lines 534-536 – Globally, it is also be important to address the NPK-ref nutrient content because this (supposedly) reference condition lack all the other macro and micronutrients that the extracts have, specially *A. nodosum*, that is much richer than *E. maxima* on most of it (S1 Table), what may explain the poor differences observed between *A. nodosum* and the NPK-ref and the poorest performance with *E. maxima.*
  - Line 539 – “higher” than what? All the other treatments? – indicate and refer to a Fig/Table.

- Yield indicators may be improved by seaweed extracts applications
  - Line 558 – it’s not “from 2022 to 2021”, is it? – correct.
  - Lines 564-568 – Regarding this discussion it is important to remember that plant/algal extracts have secondary metabolites that may confer protection against water-, light-, temperature-induced stresses, and that could explain the higher impact in warmer/driers conditions – I suggest to add that to the discussion.
  - Lines 569-570 – Before the “Additionally, although outside of the scope”, future research” should focus in many other aspects, as those already referred regarding extract application, but also chemical composition of the extracts in terms of secondary metabolites, and monitor plant water status.

Conclusions:

- Lines 577-579 – again, be clearer/more truthful and objective regarding the effect of *A. nodosum* comparing to NPK-ref, remember that it doesn’t need to be better but if it’s as good as the traditional practice NPK is already good, don’t avoid that “fight” because you don’t need to.

References:

Didn’t revise systematically but it seems ok.

Figures/Tables:

- S1 Table – species in italic (footnote); the reference to the service responsible for the chemical analysis of commercial formulations should be included.
- S2 Table – revise on footnotes “Asterisks indicate significant differences between the treatments (0.05 > P > 0.01: * ; 0.01 > P > 0.001: ** ; P < 0.001: ***).” - it should be: (0.01 < P ≤ 0.05: * ; 0.001 < P ≤ 0.01: ** ; P ≤ 0.001: ***).
- S2 Table - “Stomatal conductance (σ)” is not a fluorescence index – revise table format.
- Figure 2 – I think the units of “Leaf dry mass” are mg (compare with that of Table 1) – correct.
- Table 1 – remove the number 1 after “Comparison”, I think there’s no need, it goes with the analysis/table contents just leave the footnote (the same applies to similar

situations as in Table 2, S2 and S5 Table). Also, as the variables CCI and Fv/Fm were already specified, the numbers 2 and 3 and respective footnotes with their full names are also unnecessary. On the other hand, the numbers inside the table refer to what? Average values over time? Last time-point? Compare with footnotes in Table 2, an analogous table and better explained – clarify/ harmonize.

- Figure 3 – Change “PSII efficiency (Fv/Fm)” to “PSII maximum quantum efficiency”, “PSII maximum quantum yield” or just “Fv/Fm”.
