# Supplementary material for: Assessing the potential of seaweed extracts to improve vegetative, physiological and berry quality parameters in Vitis vinifera cv. Chardonnay under cool climatic conditions
Source: PLoS One. 2025 Sep 2;20(9):e0331039. doi: 10.1371/journal.pone.0331039 (PMC12404493; doi:10.1371/journal.pone.0331039)
Supplement: Attachment — Submitted filename: PONE-D-24-45452_reviewer_report.pdf [file pone.0331039.s014.pdf]

PLOS ONE

Assessing the potential of seaweed extracts to improve vegetative, physiological and reproductive characteristics in <i>Vitis vinifera</i> cv. Chardonnay under cool climatic conditions.

--Manuscript Draft--

**Manuscript Number:**	PONE-D-24-45452
**Article Type:**	Research Article
**Full Title:**	Assessing the potential of seaweed extracts to improve vegetative, physiological and reproductive characteristics in <i>Vitis vinifera</i> cv. Chardonnay under cool climatic conditions.
**Short Title:**	Assessing the potential of seaweed extracts to improve grapevine growth characteristics in cooler climates.
**Corresponding Author:**	Sam Crauwels<br>KU Leuven Faculty of Engineering Technology: Katholieke Universiteit Leuven Faculteit Industriele Ingenieurswetenschappen<br>Leuven, BELGIUM
**Keywords:**	<i>Ascophyllum nodosum</i>; biostimulants; <i>Ecklonia maxima</i>; Photosystems' efficiency; seaweed extracts; <i>Vitis vinifera</i>
**Abstract:**	Seaweed extracts are promising plant biostimulants for viticulture, but their effects on white winegrape cultivars grown under cool climates is fairly undocumented. Furthermore there is limited information on the efficacy of some species like <i>Ecklonia maxima</i>. This study evaluated the impact of <i>Ascophyllum nodosum</i> and <i>Ecklonia maxima</i> extracts on <i>Vitis vinifera</i> cv. Chardonnay in Belgium in the 2021 and 2022 growing seasons. Treatments, including water and an NPK-reference treatment (containing comparable amounts of nitrogen, phosphorous and potassium as the extracts), were applied as foliar sprays, five times at regular intervals, from flowering to ripening. In 2021 and 2022 <i>A. nodosum</i> significantly increased leaf area (+13% and +15%) and chlorophyll content (+12% and +4%), and was associated with a small but statistically significant improvement in photosystem II quantum yield (1.5% and +0.7%). These improvements lead to increased berry size, mass, and sugar content during the 2022 ripening period. Conversely, <i>E. maxima</i> had smaller effects on vegetative, physiological and subsequent reproductive growth. When comparing the yield between the two seasons, greater increases were observed in 2022, relative to 2021, for <i>A. nodosum</i> (+53%), <i>E. maxima</i> (+60%), and NPK-reference vines (+80%) compared to control vines (+26%). Our results indicate that seaweed extracts can enhance grapevine vegetative growth, leading to small but significant effects in reproductive parameters in cool climates. However, effect sizes on grapevines may vary between extracts (species differences and batch variation) and under different environmental conditions.
**Order of Authors:**	Johan Yssel, M.Sc.
	Vicky Everaerts, M.Sc
	Wendy Van Hemelrijck, Ph.D
	Dany Bylemans, Ph.D
	Mathabatha Evodia Setati, Ph.D
	Bart Lievens, Ph.D
	Erna Blancquaert, Ph.D
	Sam Crauwels, Ph.D
**Additional Information:**	
**Question**	**Response**

Powered by Editorial Manager® and ProduXion Manager® from Aries Systems Corporation

**Financial Disclosure**<br><br>Enter a financial disclosure statement that describes the sources of funding for the work included in this submission. Review the submission guidelines for detailed requirements. View published research articles from *PLOS ONE* for specific examples.<br><br>This statement is required for submission and **will appear in the published article** if the submission is accepted. Please make sure it is accurate.<br><br>**Funded studies**<br>Enter a statement with the following details:<br>• Initials of the authors who received each award<br>• Grant numbers awarded to each author<br>• The full name of each funder<br>• URL of each funder website<br>• Did the sponsors or funders play any role in the study design, data collection and analysis, decision to publish, or preparation of the manuscript?<br><br>Did you receive funding for this work?	Yes
Please add funding details.<br>as follow-up to "**Financial Disclosure**<br><br>Enter a financial disclosure statement that describes the sources of funding for the work included in this submission. Review the submission guidelines for detailed requirements. View published research articles from *PLOS ONE* for specific examples.<br><br>This statement is required for submission and **will appear in the published article** if the submission is accepted. Please make sure it is accurate.	Johan (DJ) Yssel<br>Project number: 3E200528<br>KU Leuven Campus Group T<br>https://www.kuleuven.be/english/campuses/group-t-leuven-campus/Research<br>The sponsors played no additional roles apart from financing.

Powered by Editorial Manager® and ProduXion Manager® from Aries Systems Corporation

**Funded studies**<br>Enter a statement with the following details:<br>• Initials of the authors who received each award<br>• Grant numbers awarded to each author<br>• The full name of each funder<br>• URL of each funder website<br>• Did the sponsors or funders play any role in the study design, data collection and analysis, decision to publish, or preparation of the manuscript?<br><br>Did you receive funding for this work?"	
Please select the country of your main research funder (please select carefully as in some cases this is used in fee calculation).<br>as follow-up to "**Financial Disclosure**<br><br>Enter a financial disclosure statement that describes the sources of funding for the work included in this submission. Review the submission guidelines for detailed requirements. View published research articles from *PLOS ONE* for specific examples.<br><br>This statement is required for submission and **will appear in the published article** if the submission is accepted. Please make sure it is accurate.<br><br>**Funded studies**<br>Enter a statement with the following details:<br>• Initials of the authors who received each award<br>• Grant numbers awarded to each author<br>• The full name of each funder<br>• URL of each funder website<br>• Did the sponsors or funders play any role in the study design, data collection and analysis, decision to publish, or preparation of the manuscript?<br><br>Did you receive funding for this work?"	BELGIUM - BE
**Competing Interests**	The authors have declared that no competing interests exist.

Use the instructions below to enter a competing interest statement for this submission. On behalf of all authors, disclose any competing interests that could be perceived to bias this work—acknowledging all financial support and any other relevant financial or non-financial competing interests.<br><br>This statement is required for submission and **will appear in the published article** if the submission is accepted. Please make sure it is accurate and that any funding sources listed in your Funding Information later in the submission form are also declared in your Financial Disclosure statement.<br><br>View published research articles from *PLOS ONE* for specific examples.<br><br>**NO authors have competing interests**<br><br>Enter: *The authors have declared that no competing interests exist.*<br><br>**Authors with competing interests**<br><br>Enter competing interest details beginning with this statement:<br><br>*I have read the journal's policy and the authors of this manuscript have the following competing interests: [insert competing interests here]*<br><br>* typeset	
**Ethics Statement**<br><br>Enter an ethics statement for this submission. This statement is required if the study involved:<br><br>• Human participants<br>• Human specimens or tissue<br>• Vertebrate animals or cephalopods<br>• Vertebrate embryos or tissues	Trial: 21MUNCINECHARP6aKULRESXX<br>Research station for Fruit Growing

• Field research<br><br>Write "N/A" if the submission does not require an ethics statement.<br><br>General guidance is provided below. Consult the submission guidelines for detailed instructions. **Make sure that all information entered here is included in the Methods section of the manuscript.**	

**Format for specific study types**<br><br>**Human Subject Research (involving human participants and/or tissue)**<br>• Give the name of the institutional review board or ethics committee that approved the study<br>• Include the approval number and/or a statement indicating approval of this research<br>• Indicate the form of consent obtained (written/oral) or the reason that consent was not obtained (e.g. the data were analyzed anonymously)<br><br>**Animal Research (involving vertebrate animals, embryos or tissues)**<br>• Provide the name of the Institutional Animal Care and Use Committee (IACUC) or other relevant ethics board that reviewed the study protocol, and indicate whether they approved this research or granted a formal waiver of ethical approval<br>• Include an approval number if one was obtained<br>• If the study involved *non-human primates*, add *additional details* about animal welfare and steps taken to ameliorate suffering<br>• If anesthesia, euthanasia, or any kind of animal sacrifice is part of the study, include briefly which substances and/or methods were applied<br><br>**Field Research**<br><br>Include the following details if this study involves the collection of plant, animal, or other materials from a natural setting:<br>• Field permit number<br>• Name of the institution or relevant body that granted permission	
**Data Availability**<br><br>Authors are required to make all data underlying the findings described fully available, without restriction, and from the time of publication. PLOS allows rare exceptions to address legal and ethical concerns. See the PLOS Data Policy and FAQ for detailed information.	Yes - all data are fully available without restriction

Powered by Editorial Manager® and ProduXion Manager® from Aries Systems Corporation

A Data Availability Statement describing where the data can be found is required at submission. Your answers to this question constitute the Data Availability Statement and **will be published in the article**, if accepted.<br><br>**Important:** Stating 'data available on request from the author' is not sufficient. If your data are only available upon request, select 'No' for the first question and explain your exceptional situation in the text box.<br><br>Do the authors confirm that all data underlying the findings described in their manuscript are fully available without restriction?	
**Describe where the data may be found in full sentences. If you are copying our sample text, replace any instances of XXX with the appropriate details.**<br><br>• If the data are **held or will be held in a public repository**, include URLs, accession numbers or DOIs. If this information will only be available after acceptance, indicate this by ticking the box below. For example: *All XXX files are available from the XXX database (accession number(s) XXX, XXX.).*<br>• If the data are all contained **within the manuscript and/or Supporting Information files**, enter the following: *All relevant data are within the manuscript and its Supporting Information files.*<br>• If neither of these applies but you are able to provide **details of access elsewhere**, with or without limitations, please do so. For example:<br><br>*Data cannot be shared publicly because of [XXX]. Data are available from the XXX Institutional Data Access / Ethics Committee (contact via XXX) for researchers who meet the criteria for access to confidential data.*<br><br>*The data underlying the results presented in the study are available from (include the name of the third party*	All relevant data are within the manuscript and its Supporting Information files.

and contact information or URL).<br>• This text is appropriate if the data are owned by a third party and authors do not have permission to share the data.<br><br>* typeset	
Additional data availability information:	Tick here if the URLs/accession numbers/DOIs will be available only after acceptance of the manuscript for publication so that we can ensure their inclusion before publication.

Powered by Editorial Manager® and ProduXion Manager® from Aries Systems Corporation

Manuscript

Click here to access/download;Manuscript;Manuscript_YSSEL_Belgium_Gr

Assessing the potential of seaweed extracts to improve vegetative, physiological and reproductive characteristics in *Vitis vinifera* cv. Chardonnay under cool climatic conditions

Johan Yssel[1,2,3], Vicky Everaerts[4], Wendy van Hemelrijk[4], Dany Bylemans[2,4], Mathabatha Evodia Setati[3], Bart Lievens[1,2], Erna Blancquaert[3], Sam Crauwels[1,2*]

[1] CMPG Laboratory for Process Microbial Ecology and Bioinspirational Management (PME&BIM), Department of Microbial and Molecular Systems (M2S), KU Leuven, Leuven, Belgium

[2] Leuven Plant Institute (LPI), KU Leuven, Leuven, Belgium

[3] South African Grape and Wine Research Institute, Stellenbosch University, Stellenbosch, South Africa

[4] Research Station for Fruit Growing, Sint-Truiden, Belgium

*Corresponding author

Email: sam.crauwels@kuleuven.be (SC)

Abstract

Seaweed extracts are promising plant biostimulants for viticulture, but their effects on white winegrape cultivars grown under cool climates is fairly undocumented. Furthermore there is limited information on the efficacy of some species like *Ecklonia maxima*. This study evaluated the impact of *Ascophyllum nodosum* and *Ecklonia maxima* extracts on *Vitis vinifera* cv. Chardonnay in Belgium in the 2021 and 2022 growing seasons. Treatments, including water and an NPK-reference treatment (containing comparable amounts of nitrogen, phosphorous and potassium as the extracts), were applied as foliar sprays, five times at regular intervals, from flowering to ripening. In 2021 and 2022 *A. nodosum* significantly increased leaf area (+13% and +15%) and chlorophyll content (+12% and +4%), and was associated with a small but statistically significant improvement in photosystem II quantum yield (1.5% and +0.7%). These improvements lead to increased berry size, mass, and sugar content during the 2022 ripening period. Conversely, *E. maxima* had smaller effects on vegetative, physiological and subsequent reproductive growth. When comparing the yield between the two seasons, greater increases were observed in 2022, relative to 2021, for *A. nodosum* (+53%), *E. maxima* (+60%), and NPK-reference vines (+80%) compared to control vines (+26%). Our results indicate that seaweed extracts can enhance grapevine vegetative growth, leading to small but significant effects in reproductive parameters in cool climates. However, effect sizes on grapevines may vary between extracts (species differences and batch variation) and under different environmental conditions.

Introduction

Grapevine is one of the most economically valuable crops in the world, with grapes being the fifth most produced fruit crop globally [1] of which 46% is used to produce wine [2]. Grapevines are cultivated under diverse, environmental conditions, with regions differing considerably in terms of growing season temperature, sunlight exposure, rainfall and humidity, wind, elevation, and soil

characteristics [3]. Most of the world's wine production today occurs in the mid-latitudes, where the average growing season temperatures vary between 13 and 22 °C [4,5]. However, climate change is generally believed to drastically change the viticultural suitability of many winemaking regions [6–8]. In fact, 90% of traditional wine regions in coastal and lowland regions of Spain, Italy, Greece and southern California could be at risk of disappearing by the end of the century [9] due to increased heat- and drought stress and changes in precipitation patterns [10–12], as well as the spread of new pests and rise in disease incidence [13]. Altogether, these challenges result in reduced yields and alterations in grape quality, resulting in lower wine quality and/or production [14,15]. Nevertheless, while certain areas are under threat, opportunities arise as new production regions emerge in the cooler regions of North-western Europe, namely Belgium, northern Germany, the Netherlands, and the United Kingdom [9].

In response to the challenges posed by climate change, growers are increasingly relying on (chemical) fertilisers and pesticides as measures to ensure the consistent production of high-quality grapes [16,17]. However, researchers have reported that the excessive application of macronutrients such as nitrogen and phosphorous leads to reduced soil health and can inadvertently favour the development of pathogens over beneficial microorganisms [18–20], thereby negatively impacting the wine quality [21]. Likewise, pesticide use has been linked with surface water contamination, reduced soil health and biodiversity [22,23], as well as human health consequences such as increased cancer risk, endocrine disruption and neurological disorders [24,25]. Thus, there is an urgent need for alternative strategies to ensure the long-term sustainability of viticulture [26–28].

The use of biostimulants has emerged as a sustainable solution, offering the potential to enhance plant growth, plant performance, stress tolerance, and crop quality in a manner that reduces the need for synthetic inputs [29–31]. Biostimulants have long been loosely defined and were often regarded dubiously because of their composite nature and the inherent difficulty to determine which specific components were making beneficial contributions. The definition proposed by du Jardin [32] which

stipulates biostimulants as “any substance or microorganism applied to plants with the aim to enhance nutrition efficiency, abiotic stress tolerance and/or crop quality traits, regardless of its nutrients content” represents one of the more clear and concise ways to define biostimulants. Biostimulants can be classified in several ways, for example based on mode of action, composition, or source origin [33]. Following the source origin approach by Yakhin [33], biostimulants are classified into seven categories, including humic substances, protein hydrolysates, seaweed extracts, chitosan and other biopolymers, inorganic compounds, beneficial fungi, and beneficial bacteria. Of these, seaweed extracts, in particular those made from brown seaweed species such as *Laminaria, Sargassum* and *Ascophyllum* [34], have shown promise in horticulture [35] as well as viticulture [21,36]. They contain various beneficial minerals, organic acids, amino acids, and plant growth regulators such as cytokinins and auxins [37–39], contributing to improved stress tolerance and crop productivity and quality [35,40–43].

While seaweed extracts have shown promise in grapevines and other horticultural crops, research is still limited on their application and effects in commercial winegrape varieties, particularly white winegrape varieties [21,44]. Furthermore, most studies focus on the effects of seaweed extracts in drought and heat-prone climates [44], while only a limited number of studies have been conducted on their beneficial effects under colder climatic conditions [21]. Seaweed extracts are also subject to great variations in composition, arising from the type of seaweed species, the production process and season-dependent batch variations [35].

The aim of this study was to assess whether the foliar application of seaweed extracts leads to improvements in vegetative-, physiological- and reproductive growth characteristics of Chardonnay vines grown in a cool climate region (Belgium). Specifically, extracts from two brown seaweed species, *Ascophyllum nodosum* and *Ecklonia maxima*, were evaluated alongside an NPK-reference treatment containing comparable concentrations of the macronutrients nitrogen, phosphorus, and potassium, as found within the seaweed extracts. *Ascophyllum nodosum* is an important species of

brown seaweed, which grows in the intertidal region of the coasts of the North Atlantic, and has demonstrated significant plant growth promoting and abiotic stress protective effects in several agricultural crops [45–47] as well as several winegrape cultivars [21,48]. In contrast, *E. maxima*, a species of brown seaweed found in the shallow, temperate regions of the Atlantic coast of Southern Africa, has remained understudied, in particular regarding the effects on grapevine vegetative growth and physiology. A limited number of studies have shown beneficial effects on vine reproductive parameters [49,50]. Our study was conducted in a cool climate region over two growing seasons (2021 and 2022), to evaluate whether the growth stimulating effects of seaweed extracts are modulated by climatic conditions. Furthermore we included two brown seaweed species to determine if these effects are dependent on the seaweed species used. The findings of this research will contribute valuable insights into the application of seaweed extracts, to enhance cool climate viticulture practices, improve- vine growth and grape ripening dynamics, and advance sustainable agriculture in white winegrape production.

Materials and methods

Vineyard layout

The study was conducted during two consecutive growing seasons (2021 and 2022) on the experimental vineyard of Research Station for Fruit Growing, located 5 km south of the town Sint-Truiden, Belgium (50.774218 N, 5.155002 E) at 75 m above sea level. A total of 120 six-year-old vines of *Vitis vinifera* cv. Chardonnay (clone ENTAV-INRA 96), grafted onto SO4 rootstocks (*Vitis berlandieri* x *Vitis riparia*) ~~was~~ included in the study. Vines were planted in sandy-loam soil in a single row within the vineyard, which in total had 22 rows of vines. Row orientation was NNW/SSE, with row and vine spacing 2.0 m and 1.0 m, respectively (S1A Fig.). The vines were trained on a six-wire vertical shoot positioning (VSP) and cane-pruned single guyot system with two spurs on alternating sides of the crown. On alternating years, a fruiting cane and a backup cane was trimmed down to 8 nodes, while on the opposing side the spur was trimmed down to two nodes from which the shoots used in the subsequent year were grown. Canopy management involved minimal leaf removal in the fruiting zone and two instances of topping (S1B Fig.).

Experimental design

Vines were subjected to four treatments according to a randomized block design. All four treatments were prepared in demineralised water at a 1:500 dilution with 0.001% Tween-20 (Merck KGaA, Darmstadt, Germany) added as a surfactant to improve foliar uptake of the treatments, according to supplier specifications. These were (i) a blank treatment with no additional compounds (Control), (ii) a reference treatment for macro-nutrient content (NPK-Ref); (iii) a commercial *A. nodosum* based seaweed extract (Acadian Plant Health, Dartmouth, Canada); and (iv) a commercial *E. maxima*-based seaweed extract (COMPO EXPERT GmbH, Münster, Germany) (S2A Fig.). The reference treatment for nutrient content was prepared from a mixture of KCl (Honeywell International Inc, Charlotte, NC) and Haifa MAP™ (Haifa Group, Haifa, Israel) to mimic the average nitrogen, phosphorus and

potassium content (NPK) of the two biostimulant treatments (S1 Table). The nutrient composition of the seaweed extracts was determined by the Soil Service of Belgium (Leuven, Belgium). The contents of P, K, Mg, Ca, Na, S were analysed by ICP-AES (inductively coupled plasma atomic emission spectroscopy), while total N and total C were determined using spectrometry, according to standard protocols, following the Belgian Accreditation Institution (BELAC) reference methods. Treatments were applied every three weeks during 2021, starting at 18 days before flowering (11 June), which corresponded to stage E-L 15 according to the modified Eichhorn-Lorenz (E-L) system [51], and every two weeks during 2022, starting 12 days before the flowering stage (E-L 18/19) (3 June). The final treatment was applied before véraison (E-L 34) in both years (3 September 2021 and 5 August 2022) (S2A Fig.). Application timepoints were based on supplier recommendations in order to target the vegetative growth stages. On alternating weeks, the vines were treated with potassium hydrogen carbonate (Karma®, Certis Europe B.V, Brussels, Belgium) as a minimal intervention approach to reduce the pressure of *Erysiphe necator* (the causal agent of powdery mildew in grapevines) (S2A Fig.). Treatments were applied in four blocks, each consisting of 30 vines, to a specific subblock consisting of five vines. Additionally, each block contained a subblock of five vines that remained untreated. Between subblocks there was another vine that remained untreated as a buffer between subblocks (S2B Fig.). Treatments were applied by spraying both sides of the canopy using a STIHL SR 430 gas-powered backpack sprayer (ANDREAS STIHL NV, Puurs-Sint-Amands, Belgium) until run-off. All sprays were administered between 8:00 and 9:00 in the morning on clear days. Samples were collected from, and measurements done on, the three inner vines of each treated subblock (3 plants × 4 blocks per treatment), to minimise the impact of treatment carryover between subblocks (S2B Fig.).

Weather data

Environmental conditions were logged via an automated weather station located in the vicinity of the vineyard. The daily maximum (T_{max}), average (T_{avg}) and minimum temperature (T_{min}), precipitation and relative humidity (RH%) were logged from 1 April to 31 October during the 2021 and 2022 seasons. Cumulative growing degree days (GDD) [52] were calculated using the following formula:

$$GDD = \frac{1}{n}\sum_{i=0}^{n}(T - T_b) \quad (1)$$

where n is the number of days, T the daily mean temperature and Tb the base temperature (10 °C) for grapevine growth. Vapour pressure deficit (VPD) was calculated hourly using the following formula proposed by [53]:

$$VPD = 0.61078 \times e^{\frac{17.2694 \cdot T}{(T+237.3)}} \times \frac{1 - RH\%}{100} \quad (2)$$

where VPD is in kPa, T is the temperature in °C, and RH% the relative humidity. VPD data were then plotted over the course of the growing season.

Vine vegetative characteristics and leaf starch content

Three representative shoots per vine were tagged on the main fruiting cane at E-L 12 in the 2021 growing season (21 May 2021). These were measured over three weeks with a measuring tape, after which the average shoot length was determined for each replicate vine. Treatments were then assigned to subblocks to ensure each treatment had vines of a comparable phenological development, with each sampled vine a replicate (n = 12) (S2B Fig.). During the 2022 growing season three new representative shoots were tagged on the fruiting cane of vines (receiving the same treatments as in 2021) and measured weekly starting at E-L 15 (18 May 2022) until one week before the start of flowering (E-L 21). Following the start of treatment application (E-L 15 on 11 June 2021 and E-L

18/19 on 3 June 2022), leaf destructive measurements were done every 2-3 weeks on a single representative, fully expanded mature leaf, exposed to sunlight, which was sampled from each vine (*n* = 12). The sampled leaves were kept on ice during sampling in the field and stored in the laboratory at -20 °C until analysis. Leaves were scanned in colour at 400 dpi using a Ricoh IM C4500 multi-function printer (Ricoh, Vilvoorde, Belgium), and the leaf area was determined using Fiji software package [54]. After scanning, leaf petioles were removed with a scalpel and the fresh weight was recorded using an analytical balance (PIONEER PX 323, Ohaus, Nänikon, Switzerland). Leaves were then dried at 70 °C until a constant dry mass was achieved to calculate the leaf water content and dry matter percentage (DM%).

The dried leaves were ground to a powder using a mortar and pestle, and the soluble sugars were extracted using an ethanol extraction step, after which the leaf total starch content was determined using a commercial kit for total starches, K-TSTA-100 (Megazyme International, Bray, Ireland). Firstly, the soluble sugars extraction was done according to [55], with the following modifications: for the ethanol extraction, 400 µL of 80% ethanol was added to 50 mg of dried leaf powder in a 2 mL screw cap tube. Then, samples were vortexed and boiled for 15 minutes at 80 °C in a heating block (Model 2053-1, Lab-Line Instruments Inc., Melrose Park, IL). Samples were then vortexed again and centrifuged at 12 500 RCF for 10 minutes and the supernatant was discarded, retaining the leaf powder pellet. These steps (adding ethanol to the pellet, vortexing, centrifuging and discarding the supernatant) were repeated another two times to ensure the majority of the soluble sugars were removed. Then the remaining pellet was dried overnight at 70 °C and the starches were extracted using the aforementioned starch extraction kit (with the protocol optimised for use with microtiter plates [56]). Briefly, this involved adding 100 µL of 80% ethanol and 250 µL of ice-cold 1.7 M NaOH (VWR, Leuven, Belgium) and two 2 mm glass beads to the screw cap tube containing the dried leaf pellet. Tubes were then vortexed, and the content was homogenised in a bead mill homogenizer (Bead Ruptor Elite, Omni International, GA, USA) using a speed of 4.5 m·s-1 with five cycles of 30 seconds each. Next, 1000 µL of a 100 mM sodium-acetate buffer at pH 3.7 was added and the tubes were

vortexed. Then, 12.5 µL of undiluted thermostable α-amylase was added, followed immediately by 12.5 µL amyloglucosidase, after which the tubes were incubated at 50 °C for 60 minutes to break down and convert the starch to D-glucose. Tubes were then centrifuged at 13 000 RCF for three minutes and the retained supernatant transferred to new 1.5 mL microcentrifuge tubes. The sample supernatants were then diluted with 100 mM sodium-acetate buffer at pH 5.0, depending on the expected final concentrations of starch (e.g., 1:4 dilution early season and 1:7 late season). Subsequently, following brief vortexing, 20 µL of each sample was transferred in duplicate to a flat bottom 96-well plate (96 MicroWell™ Plates, Nunc™, VWR, Leuven, Belgium), to which 300 µL glucose oxidase/peroxidase (GOPOD) reagent (from the K-TSTA-100A extraction kit) was added. Plates were closed and incubated at 50 °C for 20 minutes, after which the absorbance was read using a SpectraMax® ABS spectrophotometer (Molecular Devices, San Jose, CA, USA) with the wavelength set to 510 nm. The total starch was then calculated using a standard curve of glucose as specified in the kit protocol.

Chlorophyll indices and stomatal conductance

Two mature, fully expanded leaves, exposed to full sunlight, and of a similar age (corresponding to leaves between the third and fifth node of the shoots) were tagged per replicate vine ($n = 12$) for leaf physiological measurements. The leaf chlorophyll content index (CCI) was measured in duplicate on each tagged leaf (giving four readings that were averaged per replicate vine) using a CCM-200 chlorophyll content meter (Opti-Sciences, Hudson, NH) between 10:00 and 11:30 in the morning. On the same leaves and between the same time interval, the maximum quantum yield of photosystem II (Fv/Fm, a measure of plant performance and stress) was measured using a handheld Pocket PEA chlorophyll fluorimeter (Hansatech Instruments, Norfolk, United Kingdom). For reference, values around 0.83 indicate maximum quantum yield in unstressed leaves [57]. Before measuring, the leaves were dark adapted for 30 minutes and subjected to an actinic light flash of 627 nm with an intensity of 3500 µmol·m-2·s-1, to reach the maximum Fv/Fm value [58]. The readings for the two leaves were

averaged per replicate vine. Additionally, leaf stomatal conductance was measured between 11:30 and 14:00 on the abaxial (under) side of the first tagged leaf (as measured from the crown of the vine) for each replicate vine (*n* = 12). Measurements were done for 30 seconds until steady state using an SC-1 leaf porometer (METER Group, Pullman, WA, USA). Parameters were measured weekly (between 15 June and 25 August in 2021; and 15 June to 12 August in 2022), with the exception of days with rain or full cloud cover, where the measurement was taken the following week on a clear day.

Berry classical parameters and morphology

Berries at véraison (E-L 34) were sampled until harvest (E-L 38) every 14 days for the 2021 growing season, and every 10-14 days for the 2022 season, with delayed sampling of at least two days following rainfall to reduce the impact of sugars dilution (S2C Fig.). A total of about 80 representative berries were sampled from healthy clusters across the inner three vines of a treated panel (subblock) (*n* = 4), using sterile nitrile gloves, into resealable bags, sampling from the top, middle and bottom of the cluster, and alternating between sun-exposed and shaded sides. Berries were kept on ice during sampling and transport until further processing. Thirty representative berries that were used for morphological measurements (*n* = 4) were stored in resealable bags at -20 °C, while the remainder were immediately processed at the laboratory for juice analyses. Briefly, berries were crushed to obtain a representative juice sample for each subblock (*n* = 4), and the juice was decanted and centrifuged at 4000 RCM for five minutes. The clear must was used to determine total soluble solids (°Brix), pH and titratable acidity. Soluble solids were determined with a digital refractometer (HI 96801, Hanna Instruments, Temse, Belgium) in duplicate and averaged per replicate. A pH probe (HI 1131, Hanna Instruments, Temse, Belgium) was used to determine the pH, while titratable acidity (TA) was measured using the same probe in conjunction with an autotitrator (HI 902, Hanna Instruments, Temse, Belgium). Each sample consisted of 20 mL juice and 20 mL demineralized water, titrated with 0.33 M NaOH (VWR, Leuven, Belgium) to a pH of 7.01, with results reported as

tartaric acid equivalent in g/L. For berry morphological measurements, 20 undamaged berries were randomly selected from each sampled subblock (n = 4). The berry length from pedicel to the bottom and berry diameter were measured using a vernier calliper (HOLEX 412821_100, Hoffman Group, Borne, The Netherlands). Individual berry weights were also recorded on an analytical balance (PIONEER PX 323, Ohaus, Nänikon, Switzerland), taking care that the berries remained frozen and surface ice was removed. The berry volume was estimated from the berry length and diameter, and the berry density was calculated. Following this, the 20 berries were dried at 75 °C until a constant mass was observed to determine the berry dry mass and DM%. The morphological measurements of the 20 berries were averaged per replicate subblock (n = 4).

Harvest data

At technological maturity (°Brix = 21) all berry clusters were harvested using sterile nitrile gloves into separate bags for each of the inner three vines per subblock, yielding 12 bags per treatment. The clusters were counted and weighed for each replicate vine (n = 12), to obtain an average number of clusters and average cluster weight for each replicate vine. Following this, the clusters were pooled per treated block, yielding four replicates per treatment. From each replicate, 100 berries were randomly sampled, of which 30 were frozen at -20 °C for morphological measurements and the remainder were immediately crushed to juice for analysis (as described previously).

Statistical analysis

A series of linear mixed models (LMM) were fit against the longitudinal vegetative and reproductive data, with treatment, season, time, GDD, temperature, rain, and humidity, as well as all interactions as fixed factors and replicate vine (n = 12 for vegetative and yield parameters) and block (n = 4 for reproductive parameters) as random factor (performed with the 'lmer' function from the R lme4 package) [59]. The models were compared against each other for each response variable using a likelihood ratio test, while non-contributing parameters were removed until the most parsimonious

model was obtained for further analyses. In all cases the best model was selected based on the Aikaike Information Criterion (AIC). The best model for the dataset consisting of plant vegetative and physiological parameters had treatment, time, season and the interaction between treatment and season, and time and season as fixed factors, with replicate as random factor, while the best model for the dataset consisting of plant reproductive parameters had treatment, time, season and GDD as fixed factors and block as random factor. Within season analyses followed the same approach but excluding 'season' from the initial model. An analysis of variance Type III test was performed on all models to determine if there was an overall difference between the different treatments, timepoints and seasons. The analysis was followed by a *post hoc* pairwise comparison with estimated marginal means, using the 'emmeans' package [60]. For the end-point harvest parameters, data were assessed for normality and equality of variances and analysed via a Generalized Linear Model (GLM) based on a Gamma distribution with a log link function using treatment, season, and their interaction as fixed factors (performed with the 'glm' function from the lme4 package) [59]. To evaluate overall differences between the different treatments and growing seasons, an analysis of variance (ANOVA) Type III test was performed on all models. When an overall difference was observed, a *post hoc* pairwise comparison (with estimated marginal means using the emmeans package) was performed to determine the pairwise differences between the different treatments and seasons [60]. A significance level of $\alpha = 0.05$ was used to determine significant differences in all analyses, and results were visualized using the ggplot2 package [61]. All analyses and visualization of the data were performed in R version 4.2.3 [62].

Results

Weather data

Whereas the nine-year average of GDDs between 2013 and 2021 was 1114, during the 2021 growing season (April to October) 1001 GDDs were accumulated, while 2022 saw a total of 1230 GDDs. In 2021, temperatures were cooler than average in April (-2.8 °C) and May (-0.9 °C), warmer in June

(+2.0 °C), and cooler in July (-1.9 °C) and August (-1.8 °C) (Fig 1A). Rainfall was significantly higher in July (231 mm vs 53 mm) and August (136 mm vs 62 mm), with lower than average rainfall in September (28 mm vs 45 mm) (Fig 1B). VPD exceeded 3 kPa only on three days before anthesis (flowering) (Fig 1C). In 2022, compared to the nine-year average (2013 to 2021) April was slightly cooler (-0.7 °C), followed by a warmer May (+2.4 °C), average June and July, and warmer August (+2.1 °C), with a cooler September (-1.1 °C) (Fig 1A). Rainfall was above average from April to June, but below average in July (9 mm vs 53 mm) and August (8 mm vs 62 mm), with higher than average rainfall in September (116 mm vs 45 mm) (Fig 1B). The 2022 season had elevated VPD values post-anthesis (flowering), exceeding 3 kPa on 18 days, peaking at 6.7 kPa at 34 days after anthesis (Fig 1C).

Fig 1. (A) Mean daily temperature and growing degree days (GDD), (B) monthly rainfall and vapour pressure deficit (VPD) for the 2021 and 2022 seasons. (A) Mean daily temperatures are indicated by blue circles for 2021, red triangles for 2022, and a purple dashed line for the average of the preceding eight years (2013-2020). The purple shading indicates the range over this period, and solid lines represent the cumulative growing degree days (GDD). The maximum daily vapour pressure deficit (VPD) for is shown with blue circles for 2021 and red triangles for 2022 (C).

Seaweed extract effect on grapevine vegetative growth and physiology

In 2021, the vines were treated with a negative control (water), two seaweed extracts based on *A. nodosum* and *E. maxima*, and an NPK-reference treatment. At that time, there was no significant difference in shoot length between the different treatment groups (P = 0.107) (S4 Fig.). In the following season (2022) the average shoot length of three tagged shoots emerging from the fruiting cane was found to be significantly influenced by treatment (P = 0.008) and time (date of measurement) (P < 0.001). Specifically, the NPK-treated vines had significantly longer shoot lengths on average compared to water treated vines (36.8 ± 2.2 cm vs 29.7 ± 2.0, P = 0.006) over the 2022

growing season, while no differences were observed for the seaweed extract treated vines relative to control vines (S4 Fig.).

For vegetative growth parameters, results showed strong main effects of time (development stage) on the leaf area, leaf dry mass, leaf dry matter (DM%) and leaf starch content (S2 Table.). Season showed a strong effect on leaf area and dry mass, with a less pronounced effect on starch content. Treatment had a significant effect on leaf area ($F_{3,8} = 28.60$, $P < 0.001$) and leaf dry mass ($F_{3,8} = 13.20$, $P = 0.004$), but not on specific leaf area, leaf DM% and leaf starch content. Leaf starch content had a moderate interaction effect between time and season (S2 Table.).

Vines treated with the *A. nodosum* extract had significantly larger leaves in 2021 (+13%, $P = 0.007$) and 2022 (+15%, $P < 0.001$) compared to control vines. This increase was not significant compared to the NPK-reference treatment, which had 5% and 18% larger leaves in the respective years compared to the control vines (Table 1). There was no evidence that *E. maxima* increased leaf area relative to control vines in 2021 (+6%) and 2022 (+8%) (Fig 2A and B, Table 1). While a trend of increased leaf dry mass compared to control vines was observed for the *A. nodosum* extract (+13% in 2021 and +14% in 2022), *E. maxima* extract (+12% in 2021 and +9% in 2022) and the NPK-reference treatment (+3% in 2021 and +15% in 2022) (Fig 2C and D, Table 1), the increase was only significant for the NPK-reference treatment in 2022 (Table 1).

Table 1. Comparison1 of treatment effects on vegetative growth and physiological parameters of *V. vinifera* cv. Chardonnay for the 2021 and 2022 seasons.

Treatment	Leaf area (cm^2)	Leaf dry mass (mg)	Fv/Fm[2]	CCI[3]	Stomatal conductance
2021					
Control	177 ± 4 [a]	1137 ± 39 [a]	0.772 ± 0.003 [ab]	16.0 ± 0.3 [a]	383 ± 13 [a]
A. nodosum	199 ± 6 [b]	1287 ± 43 [a]	0.777 ± 0.003 [b]	17.9 ± 0.3 [b]	383 ± 13 [a]
E. maxima	187 ± 5 [ab]	1277 ± 47 [a]	0.770 ± 0.003 [ab]	16.1 ± 0.3 [a]	382 ± 16 [a]
NPK-Ref	185 ± 5 [ab]	1170 ± 44 [a]	0.766 ± 0.003 [a]	16.6 ± 0.3 [a]	374 ± 15 [a]
P-value[1]	0.016	0.024	0.035	< 0.001	0.895
2022					
Control	140 ± 4 [a]	897 ± 33 [a]	0.788 ± 0.002 [a]	11.8 ± 0.1 [a]	263 ± 8 [a]
A. nodosum	161 ± 4 [b]	1022 ± 45 [ab]	0.793 ± 0.002 [b]	12.3 ± 0.2 [a]	266 ± 9 [a]
E. maxima	151 ± 4 [ab]	978 ± 34 [ab]	0.792 ± 0.002 [ab]	12.1 ± 0.2 [a]	254 ± 9 [a]
NPK-Ref	165 ± 5 [b]	1033 ± 40 [b]	0.794 ± 0.002 [b]	12.0 ± 0.2 [a]	253 ± 8 [a]
P-value[1]	0.001	0.042	0.007	0.211	0.508

[1] Based on ANOVA on four panels per treatment (n = 4) measured over the course of 12 weeks with biweekly measurements (Linear Mixed Model). Different letters indicate differences between treatments based on estimated marginal means ($P < 0.05$).

[2] Maximum quantum yield of photosystem II.

[3] Chlorophyll content index.

Fig 2. The evolution of leaf dry mass (2021, A; 2022, B) and leaf surface area (2021, C; 2022, D) of *V. vinifera* cv. Chardonnay treated with a water control, an *A. nodosum* extract, an *E. maxima* extract and an NPK-reference treatment. Measurements and samples were taken on clear and dry days. Each value represents the mean ± standard error (n = 12). Timepoints within seasons that showed significant differences between treatments are marked with an asterisk (ANOVA, $P < 0.05$). Central boxplots show results over all timepoints per season, with the lower, middle and upper lines of the boxplot representing the first quartile, median and third quartile, and whiskers extending to 1.5 times the interquartile range from the median. Treatments that showed significantly different responses from others within a season are indicated with unique letters based on their estimated marginal means ($P < 0.05$).

To evaluate the effect of the seaweed extracts on vine physiology, the chlorophyll content index (CCI), the quantum yield of photosystem II (Fv/Fm), and the stomatal conductance of the vines were measured. The results showed strong main effects of season and time (development stage) on the CCI, Fv/Fm and the stomatal conductance of the vines (S2 Table.). The effect of treatment was strong

on the CCI ($F_{3,17} = 41.89$, $P < 0.001$) but less pronounced on the fluorescence index Fv/Fm ($F_{3,24} = 9.592$, $P = 0.022$). Furthermore, treatment was associated with differences in stomatal conductance. However, these effects were qualified by a significant interaction between treatment and season for CCI, and time and season for Fv/Fm and the stomatal conductance (S2 Table.).

In 2021 between full-bloom (E-L 23) and the onset of berry softening (E-L 34), vines treated with the *A. nodosum* extract had a 12% higher CCI compared to negative control vines ($P < 0.001$), and an 8% higher CCI compared to NPK-treated vines ($P = 0.001$). In contrast, neither the *E. maxima* extract, nor the NPK-reference treatment had a significant impact on the CCI compared to the negative control vines (Fig 3A, Table 1). In the experiment done in 2022, the CCI was not significantly affected relative to control vines by *A. nodosum,* the NPK-reference treatment nor the *E. maxima* extract (Fig 3B, Table 1).

Vines treated with the *A. nodosum* extract had significantly higher Fv/Fm values on average during the 2021 season compared to the NPK-reference treated vines (+1.5%), but not compared to the negative control-treated vines. This contrasts with 2022, where vines treated with *A. nodosum* had a significantly higher average Fv/Fm value compared to negative control vines (+0.7%), but not compared to the NPK-reference treatment (Fig 3D, Table 1). Furthermore, *E. maxima* treatments did not significantly affect vines' Fv/Fm compared to control nor to the NPK-reference in both years during the growing period (Table 1).

Additionally, a significant difference in Fv/Fm was observed in the studied vines two weeks following a heat stress event in 2022 at 34 days after anthesis (E-L 32). During this time a maximum temperature of 39.5 °C was reached, while the vapour pressure deficit (VPD) exceeded 3 kPa for 21 hours over the preceding two days, peaking at 6.7 kPa. Fourteen days following this event, maximum temperatures remained well below 30 °C and VPD rarely exceeded 2 kPa (S3 Fig.). Vines showed a significant increase in Fv/Fm values compared to the control vines when treated with the *E. maxima* extract (+ 3.0%) and the NPK-reference treatment (2.6%), while vines treated with the *A. nodosum* did not show a significant improvement, 0.773 ± 0.006 (S4 Table.).

Fig 3. The evolution the leaf chlorophyll content index (2021, A; 2022, B) and leaf photosystem II efficiency (2021, C; 2022, D) of *V. vinifera* cv. Chardonnay treated with a water control, an *A. nodosum* extract, an *E. maxima* extract and an NPK-reference treatment. Measurements and samples were taken on clear and dry days. Each value represents the mean ± standard error ($n = 12$). Timepoints within seasons that showed significant differences between treatments are marked with an asterisk (ANOVA, $P < 0.05$).

Seaweed extract effect on reproductive parameters

The results of the reproductive parameter measurements showed strong main effects of season and time (development stage) on berry volume, berry mass, berry density and berry DM% (S5 Table.). Treatment was strongly associated with differences in berry volume ($F_{3,11} = 16.45$, $P < 0.001$) and mass ($F_{3,11} = 19.60$, $P < 0.001$), but not for density and DM%, similar to the results observed across different seasons (S5 Table.). The GDD accumulated over the season also had a strong effect on berry volume, mass, and density, with a less pronounced effect on DM% (S5 Table.). No interaction effects were detected between response variables for all the berry anatomical parameters investigated and were excluded from the final models.

Differences in berry volume were not associated with treatment in the experiment performed in 2021 ($P = 0.076$). However, in 2022, vines treated with the *A. nodosum* extract had an 8% increase in berry volume compared to the negative control when averaged over the ripening period, while NPK-reference treated vines had a 7.6% increase. This contrasts with the *E. maxima* extract-treated vines, which showed no significant increase in berry volume (Fig 4B, Table 2). Similarly, differences in berry mass were not associated with treatment in the experiment performed in 2021 ($P = 0.067$). However, in 2022 berries from vines treated with *A. nodosum* were on average 8.3% heavier during ripening and those from NPK-reference treated vines were 8.3% heavier, compared to berries from negative control treated vines. Berries from vines treated with *E. maxima* were not significantly different from any treatment (Fig 4D, Table 2).

Table 2. Comparison[1] of treatment effects on reproductive growth parameters of *V. vinifera* cv. Chardonnay for the 2021 and 2022 seasons.

Treatment	Berry volume[2] (mL)	Berry mass[2] (g)	Titratable acidity[3] (g/L)	Soluble solids[3] (°Brix)	Sugar content[3] (mg/berry)
			2021		
Control	1.49 ± 0.05 [a]	1.68 ± 0.05 [a]	10.2 ± 0.2 [a]	20.5 ± 0.1 [a]	347 ± 14 [a]
A. nodosum	1.58 ± 0.03 [a]	1.76 ± 0.04 [a]	10.4 ± 0.1 [a]	20.4 ± 0.3 [a]	361 ± 6 [a]
E. maxima	1.53 ± 0.04 [a]	1.72 ± 0.04 [a]	10.4 ± 0.2 [a]	20.8 ± 0.4 [a]	366 ± 24 [a]
NPK-Ref	1.58 ± 0.05 [a]	1.77 ± 0.05 [a]	10.3 ± 0.2 [a]	21.2 ± 0.6 [a]	383 ± 17 [a]
P-value[1]	0.076	0.085	0.220	0.031	0.008
			2022		
Control	1.44 ± 0.04 [a]	1.61 ± 0.05 [a]	7.2 ± 0.4 [a]	21.2 ± 0.2 [a]	373 ± 16 [a]
A. nodosum	1.56 ± 0.05 [b]	1.75 ± 0.06 [b]	8.3 ± 1.0 [a]	20.7 ± 0.6 [a]	396 ± 25 [a]
E. maxima	1.54 ± 0.04 [ab]	1.70 ± 0.05 [ab]	7.4 ± 0.3 [a]	21.0 ± 0.2 [a]	382 ± 13 [a]
NPK-Ref	1.55 ± 0.05 [b]	1.74 ± 0.06 [b]	7.8 ± 0.3 [a]	21.3 ± 0.3 [a]	400 ± 13 [a]
P-value[1]	0.019	0.008	0.389	0.151	< 0.001

[1] Based on ANOVA on four panels per treatment (n = 4) measured over the course of seven weeks with biweekly measurements (Linear Mixed Model). Different letters indicate differences between treatments based on estimated marginal means ($P < 0.05$)

[2] Results averaged over measurement timepoints and reported as mean ± standard error. Different letters indicate differences between treatments within a season, with $\alpha = 0.05$.

[3] Results at harvest and reported as mean ± standard error. Different letters indicate differences between treatments at harvest, with $\alpha = 0.05$.

Fig 4. Berry volume (2021, A; 2022, B) and berry mass (2021, C; 2022, D) of *V. vinifera* cv. Chardonnay treated with water, an *A. nodosum* extract, an *E. maxima* extract and an NPK-reference treatment. Samples were taken every 10-14 days except when the preceding days received rain. Each value represents the mean ± standard error (n = 4). Timepoints within seasons that showed significant differences between treatments are marked with an asterisk (ANOVA, $P < 0.05$). Central boxplots show the final timepoint at harvest, with the lower, middle and upper lines of the boxplot representing the first quartile, median and third quartile, and whiskers extending to 1.5 times the interquartile range from the median. Treatments that showed significantly different responses from others, within a season, at harvest are indicated with unique letters based on their estimated marginal means ($P < 0.05$).

Additionally, the berry classical parameters of titratable acidity TA, pH, soluble solids (°Brix) and berry sugar content were determined to evaluate the effect of treatment on ripening dynamics. The results showed strong main effects of time (development stage) on all classical parameters, while

season showed strong effects on TA and pH (S5 Table.). Treatment was strongly associated with differences in berry sugar content ($F_{3,11}$ = 27.82) but less pronounced on the soluble solids content ($F_{3,11}$ = 9.349), while being insignificant on pH and TA (Fig 5A and B, S5 Table.). Furthermore, the GDD accumulated during the season had a strong effect on TA and pH, and a less pronounced effect on berry sugar content (S5 Table.). No interaction effects were detected between response variables for all the berry classical parameters investigated and were excluded from the final models.

Berry soluble solids content (°Brix) was significantly associated with treatment in the experiment performed in 2021 (P = 0.031), but not in the experiment performed in 2022 (P = 0.151). Specifically, when compared to control vines, *A. nodosum* extract treated vines were associated with average °Brix values of 5.1% higher (P = 0.040) during the 2021 ripening period. However, by harvest vines treated with *A. nodosum* reached an average of 20.4 ± 0.3 °Brix which was comparable to the control vines at 20.5 ± 0.1 °Brix. The NPK-reference treated vines and *E. maxima* treated vines had °Brix values that were not significantly higher during the season nor at harvest (Fig 5C and D, Table 2).

Sugar content per berry was significantly associated with treatment in 2021 (P = 0.008) and more strongly in 2022 (P < 0.001), with *A. nodosum* treated vines having a 9% higher average berry sugar content over the ripening period in 2021 (P = 0.036) and 10% higher in 2022 (P = 0.033) compared to negative control vines. At harvest, however, berry sugar content was only 5% and 6% higher for *A. nodosum* vines compared to control vines in 2021 (361 ± 6 mg, P = 0.823) and 2022 (396 ± 25 mg, P = 0.631), respectively (Fig 5E and F, Table 2). Vines treated with the NPK-reference solution showed a similar significant trend, with 11% more sugar per berry during 2021 (P = 0.031) and 2022 (P = 0.005), as well as 11% more sugar at harvest in 2021 and 7% more in 2022 when compared to the control treatment (383 ± 17 mg and 400 ± 13 mg). In contrast, *E. maxima* treated vines did not show significant increases in sugar content per berry during both seasons, nor at harvest (Fig 5E and F, Table 2).

Fig 5. Development of titratable acidity (2021, A; 2022, B), soluble solids (2021, C; 2022, D) and berry sugar content (2021, E; 2022, F) of *V. vinifera* cv. Chardonnay treated with a water control, an *A. nodosum* extract, an *E. maxima* extract and an NPK-reference treatment. Samples were taken every 10-14 days except when the preceding days received rain. Timepoints within seasons that showed significant differences between treatments are marked with an asterisk (ANOVA, $P < 0.05$). Central boxplots show the final timepoint at harvest, with the lower, middle and upper lines of the boxplot representing the first quartile, median and third quartile, and whiskers extending to 1.5 times the interquartile range from the median. Treatments that showed significantly different responses from others, within a season, at harvest are indicated with unique letters based on their estimated marginal means ($P < 0.05$).

Seaweed extract effect on harvest parameters

At harvest. the treatments were not significantly associated with differences in the number of berry clusters per vine ($P = 0.551$) (Fig 6A and B), cluster mass ($P = 0.929$) (Fig 6C and D) or yield per vine ($P = 0.783$) (Fig 6E and F), with season being the main driver of differences for these parameters ($P < 0.001$). However, a large increase in yield within treatments was observed in 2022 compared to 2021, with vines treated with *A. nodosum, E. maxima* and the NPK-reference treatment yielding respectively 53%, 60% and 80% extra on average in 2022 relative to 2021, compared to the control treated vines yielding only 28% in surplus (Fig 6E and F). Post-hoc comparisons between years for each treatment revealed that yield increase was only significant for NPK-reference treated vines ($P = 0.010$).

Fig 6. Total number of clusters (2021, A; 2022, B), average cluster mass (2021, C; 2022, D) and average yield per vine (2021, E; 2022, F) of *V. vinifera* cv. Chardonnay treated with a water control treatment, an *A. nodosum* extract, an *E. maxima* extract and an NPK-reference treatment (*n* = 12). Samples were taken at technical maturity (average of 21 °Brix). Shaded violin plots show the distribution of data, bar hinges represent quartile values, while whiskers extend to 1.5 times the inter quartile range and outliers are indicated as circled dots. Treatments that showed significantly different responses from others at harvest, over both seasons, are indicated with unique letters based on their estimated marginal means ($P < 0.05$).

Discussion

In this study, the effect of two seaweed-based foliar sprays (*Ascophyllum nodosum* and *Ecklonia maxima*) on the vegetative and reproductive growth characteristics of grapevine was investigated over two consecutive growing seasons (2021 and 2022) in a cool-climate vineyard (Köppen–Geiger classification *Cfb*, with a mean average temperature across both seasons of below 16 °C) in Belgium, where viticulture is substantially expanding in recent years. Below the most important results are discussed.

Seaweed extracts show environment dependent improvements in leaf anatomy and vine physiological parameters.

Grapevine leaves are crucial for photosynthesis, supporting various growth aspects such as ripening berries and new shoot growth [63]. Evaluating parameters like leaf area, leaf mass, and specific leaf area provides insights into vegetative growth [64]. In our study, the *A. nodosum* extract significantly increased total leaf area of vines (+13% compared to control) in 2021, similar to the NPK-reference treatment. This aligns with findings where *A. nodosum* was applied on other white winegrape cultivars such as Narince [65], and white table grape cultivars such as Perlette [42], under warm climate conditions.

Photosynthetic capacity, assessed through chlorophyll fluorescence measurements, showed that the *A. nodosum* extract enhanced vine photosynthesis during cool and cloudy weather conditions in 2021, with a significantly improved chlorophyll content index (CCI) (+12%) and a significant, yet small, improvement in quantum yield of photosystem II (Fv/Fm) (+1.5%). This effect was less pronounced in 2022 due to more optimal growing conditions (warmer and sunnier), aligning with observations that higher light and temperature conditions correlate with reduced chlorophyll production and enhanced photosystem efficiency [66,67]. Conversely, *E. maxima* showed limited effects on photosynthetic capacity, though it did result in a significantly improved Fv/Fm compared to the water control, two weeks following a heat stress event at the start of véraison in 2022 (maximum temperature of 39.5°C and VPD of 6.7 kPa, (Fig 1A and C)). Based on the nutrient analyses of the extracts, *A. nodosum* had ~59 times more sulphur and ~73 times more magnesium compared to *E. maxima* (S1 Table.). Sulphur is a key component in the creation of chlorophyll molecules and magnesium an important co-factor for the activation of important enzymes like rubisco [68].

Previous studies have documented the potential of *A. nodosum* extracts to improve water relations while retaining stomatal opening under water-stressed conditions on some grape varieties [45,47]. However, our results showed no significant differences in stomatal conductance under dry conditions in 2022, potentially due to Chardonnay's anisohydric nature which allows it to retain photosynthetic performance through lower stomatal sensitivity, at the cost of more variable midday leaf water potentials [69,70].

Overall, *A. nodosum* extracts can promote vegetative growth in oceanic climate conditions (Köppen–Geiger *Cfb*), potentially enhancing photosynthetic capacity and energy availability during later growth stages [71]. These findings underscore the potential benefits of *A. nodosum* extracts but also highlight the need to optimize the source-sink balance for improved yield and berry quality [72].

Berry morphology and classical parameters are positively impacted by seaweed extracts in the warmer second season.

Berry morphology was positively associated with *A. nodosum* treated vines, with a +4.8% increase in volume in 2021 and a significant +8.3% increase in 2022 compared to control vines. A similar trend is observed for berry mass, with an increase of +6% in 2021 and +8.3% in 2022 (comparable to 6.9% for *E. maxima* treated vines and 7.6% for NPK-reference treated vines). These improvements are likely due to the larger leaf area resulting from treatments over both years, leading to increased carbon assimilation and sugar loading, thereby increasing the osmotic pressure and enhancing water uptake in the berries [73]. The stronger trend in 2022 could be explained by the warmer, sunnier conditions during ripening, which further enhanced the photosynthetic capacity of the plants. Previous studies corroborate these findings, showing that *A. nodosum* extracts enhance berry size and mass in white wine- and table grape varieties [42,65,74]. Conversely, *E. maxima* showed limited impact on berry morphology, aligning with a study on three red winegrape varieties [50]. Nutrient content may also play a role, with *A. nodosum* containing ~144 time more potassium than *E. maxima* (S1 Table.), which is particularly important as a regulator of osmotic pressure and thus berry expansion [68]. Classical ripening parameters, including soluble solids (°Brix) and berry sugar content, were also positively associated with *A. nodosum* treatments. In both seasons, *A. nodosum* treated vines had significantly higher °Brix and sugar content during the season. However, as the berries progressed through maturation, the rate of sugar loading started decreasing, allowing all vines to reach comparable °Brix by harvest. Comparing the sugar loading curves for each treatment to the sugar content of the control vines at harvest, it appears that NPK-reference, *A. nodosum*, and *E. maxima* treated vines may have reached these levels earlier in both seasons (ordered from earliest to latest). These results have implications for wine style, as titratable acidity and pH showed no significant differences between treatments during the season and at harvest, consistent with existing literature [42,50,65,74–79]. Additionally, as mentioned, the K content of *A. nodosum* was ~144 times

greater than what was determined for *E. maxima*, being more comparable to the NPK-reference treatment. Potassium plays a key role the translocation of water and sugars from the leaves to the berries, thereby affecting berry development and ripening [68], although it should be noted the extract only contained about 0.3% (S1 Table.) of the amount of K removed by the fruit crop (sink) in a study done by [80].

Yield indicators may be improved by seaweed extract applications.

Yield indicators include the total yield per vine, the number of clusters per vine, and the average cluster mass per vine. In our study, no significant differences were observed between treatments in the 2021 season. This aligns with expectations, as yield components are heavily influenced by the prior year's environmental conditions and interventions, which affect inflorescence formation and cluster number, contributing 60% to the final yield [81,82]. Comparing the yield within treatments from 2022 to 2021, vines treated with *A. nodosum* (+53%), *E. maxima* (+60%), and NPK-nutrient solution (+80%) had a greater increase compared to control vines (+28%). The differences in yield increase seems to be driven mainly by an increased berry mass, due to treatment-enhanced photosynthetic capacity (larger leaves, higher Fv/Fm). However, except for the NPK-reference treated vines, these increases were not statistically significant due to high variation between vines within treatments, especially on the level of cluster number.

This finding contrasts with previous reports of significant yield improvements in hot semi-arid climates following treatment for Chardonnay [48], Thompson seedless [74], Narince [65], and Merlot [77], and in humid subtropical climates for Niágara Rosada [83]. Studies in more comparable climate regions showed no significant yield improvements relative to control vines in red winegrape varieties [50,75,76,79].

Future research should also investigate the effect of different extraction methods on the composition of the seaweed extracts. Additionally, although outside of the scope of this study, the eventual wine quality could be impacted by seaweed extracts by, for example, impacting key metabolic pathways

during ripening, or altering the composition of the microbiome on the berries and leaves, which could be important aspects for future research.

Conclusions

This study investigated the effects of *A. nodosum* and *E. maxima* seaweed-based foliar sprays on grapevine growth over two seasons in an emerging cool-climate wine region in Belgium. This is a first report demonstrating positive effects of *A. nodosum* on leaf area and photosynthetic capacity under cool and cloudy conditions, leading to increased berry size and sugar content during ripening, and improved yields in a subsequent season. While *E. maxima* did not demonstrate the same effect sizes in terms of leaf growth, it did show promise in helping vines recover from heat stress, while also leading to improved yields. A likely reason is the significant differences in key nutrients between the extracts, with *A. nodosum* containing significantly more magnesium, potassium and sulphur. This study underscores the importance of understanding the composition of seaweed extracts on a batch and species level.

Acknowledgments

The authors would like to thank L. Decorte, T. Kocijan, A. Papageorgiou, A. White, and for their assistance with the field measurements and F.A.C. van Neerbos for her assistance with the data analysis. Furthermore, the authors thank COMPO Expert GmbH and Acadian Plant Health Ltd. for supplying the seaweed extracts used in this study.

References

1. FAO. Agricultural production statistics 2000–2022. FAOSTAT Analytical Briefs. 2023;79. doi:https://doi.org/10.4060/cc9205en

2. OIV. Annual Assessment of the World Vine and Wine Sector in 2022. 2022. Available: https://www.oiv.int/sites/default/files/documents/OIV_Annual_Assessment-2023.pdf

3. Anderson K, Nelgen S. Which Winegrape Varieties are Grown Where? 1st ed. Adelaide: University of Adelaide Press; 2020. Available: https://economics.adelaide.edu.au/wine-economics/ua/media/27/winegrapes-revised-ebook-0920.pdf

4. Jones GV, Reid R, Vilks A. Climate, Grapes, and Wine: Structure and Suitability in a Variable and Changing Climate. In: Dougherty PH, editor. The Geography of Wine: Regions, Terroir and Techniques. Dordrecht: Springer Netherlands; 2012. pp. 109–130. doi:10.1007/978-94-007-0464-0

5. Jones GV. Climate and Terroir: Impacts of climate variability and change on wine. 9th ed. In: R.W. Macqueen, L.D. Meinert, editors. Fine Wine and Terroir—The Geoscience Perspective. 9th ed. St John's, NL, Canada: Geoscience Canada, Geological Association of Canada; 2006.

6. Hannah L, Roehrdanz PR, Ikegami M, Shepard A V., Shaw MR, Tabor G, et al. Climate change, wine, and conservation. Proceedings of the National Academy of Sciences. 2013;110: 6907–6912. doi:10.1073/pnas.1210127110

7. Venios X, Korkas E, Nisiotou A, Banilas G. Grapevine Responses to Heat Stress and Global Warming. Plants. 2020;9: 1754. doi:10.3390/plants9121754

8. Sgubin G, Swingedouw D, Mignot J, Gambetta GA, Bois B, Loukos H, et al. Non-linear loss of suitable wine regions over Europe in response to increasing global warming. Glob Chang Biol. 2023;29: 808–826. doi:10.1111/gcb.16493

9. van Leeuwen C, Sgubin G, Bois B, Ollat N, Swingedouw D, Zito S, et al. Climate change impacts and adaptations of wine production. Nat Rev Earth Environ. 2024;5: 258–275. doi:10.1038/s43017-024-00521-5

10. Delgado-Baquerizo M, Guerra CA, Cano-Díaz C, Egidi E, Wang J-T, Eisenhauer N, et al. The proportion of soil-borne pathogens increases with warming at the global scale. Nat Clim Chang. 2020;10: 550–554. doi:10.1038/s41558-020-0759-3

11. Trivedi P, Batista BD, Bazany KE, Singh BK. Plant–microbiome interactions under a changing world: responses, consequences and perspectives. New Phytologist. 2022;234: 1951–1959. doi:10.1111/nph.18016

12. Wing IS, De Cian E, Mistry MN. Global vulnerability of crop yields to climate change. J Environ Econ Manage. 2021;109: 102462. doi:10.1016/j.jeem.2021.102462

13. Chaloner TM, Gurr SJ, Bebber DP. Plant pathogen infection risk tracks global crop yields under climate change. Nat Clim Chang. 2021;11: 710–715. doi:10.1038/s41558-021-01104-8

14. Mira de Orduña R. Climate change associated effects on grape and wine quality and production. Food Research International. 2010;43: 1844–1855. doi:10.1016/j.foodres.2010.05.001

15. van Leeuwen C, Destrac-Irvine A, Dubernet M, Duchêne E, Gowdy M, Marguerit E, et al. An Update on the Impact of Climate Change in Viticulture and Potential Adaptations. Agronomy. 2019;9: 514. doi:10.3390/agronomy9090514

16. Pretty J. Agricultural sustainability: concepts, principles and evidence. Philosophical Transactions of the Royal Society B: Biological Sciences. 2008;363: 447–465. doi:10.1098/rstb.2007.2163

17. Nesbitt A, Kemp B, Steele C, Lovett A, Dorling S. Impact of recent climate change and weather variability on the viability of UK viticulture - combining weather and climate records with producers' perspectives. Aust J Grape Wine Res. 2016;22: 324–335. doi:10.1111/ajgw.12215

18. Lekberg Y, Arnillas CA, Borer ET, Bullington LS, Fierer N, Kennedy PG, et al. Nitrogen and phosphorus fertilization consistently favor pathogenic over mutualistic fungi in grassland soils. Nat Commun. 2021;12: 3484. doi:10.1038/s41467-021-23605-y

19. Searchinger T, Hanson C, Ranganathan J, Lipinski B, Waite R, Winterbottom R, et al. Creating a Sustainable Food Future: Interim Findings. 2013 Feb. Available: https://www.wri.org/research/creating-sustainable-food-future-interim-findings

20. Veresoglou SD, Barto EK, Menexes G, Rillig MC. Fertilization affects severity of disease caused by fungal plant pathogens. Plant Pathol. 2013;62: 961–969. doi:10.1111/ppa.12014

21. Gutiérrez-Gamboa G, Moreno-Simunovic Y. Seaweeds in viticulture: a review focused on grape quality. Ciência e Técnica Vitivinícola. 2021;36: 9–21. doi:10.1051/ctv/20213601009

22. de Souza RM, Seibert D, Quesada HB, de Jesus Bassetti F, Fagundes-Klen MR, Bergamasco R. Occurrence, impacts and general aspects of pesticides in surface water: A review. Process Safety and Environmental Protection. 2020;135: 22–37. doi:10.1016/j.psep.2019.12.035

23. Karpouzas DG, Papadopoulou E, Ipsilantis I, Friedel I, Petric I, Udikovic-Kolic N, et al. Effects of nicosulfuron on the abundance and diversity of arbuscular mycorrhizal fungi used as indicators of pesticide soil microbial toxicity. Ecol Indic. 2014;39: 44–53. doi:10.1016/j.ecolind.2013.12.004

24. Kaur R, Choudhary D, Bali S, Bandral SS, Singh V, Ahmad MA, et al. Pesticides: An alarming detrimental to health and environment. Science of The Total Environment. 2024;915: 170113. doi:10.1016/j.scitotenv.2024.170113

25. Rani L, Thapa K, Kanojia N, Sharma N, Singh S, Grewal AS, et al. An extensive review on the consequences of chemical pesticides on human health and environment. J Clean Prod. 2021;283: 124657. doi:10.1016/j.jclepro.2020.124657

26. Palliotti A, Tombesi S, Silvestroni O, Lanari V, Gatti M, Poni S. Changes in vineyard establishment and canopy management urged by earlier climate-related grape ripening: A review. Sci Hortic. 2014;178: 43–54. doi:10.1016/j.scienta.2014.07.039

27. Palliotti A, Tombesi S, Frioni T, Famiani F, Silvestroni O, Zamboni M, et al. Morpho-structural and physiological response of container-grown Sangiovese and Montepulciano cvv. (*Vitis vinifera*) to re-watering after a pre-veraison limiting water deficit. Functional Plant Biology. 2014;41: 634. doi:10.1071/FP13271

28. Poni S, Gatti M, Palliotti A, Dai Z, Duchêne E, Truong T-T, et al. Grapevine quality: A multiple choice issue. Sci Hortic. 2018;234: 445–462. doi:10.1016/j.scienta.2017.12.035

29. Rouphael Y, Colla G. Editorial: Biostimulants in Agriculture. Front Plant Sci. 2020;11. doi:10.3389/fpls.2020.00040

30. Colla G, Rouphael Y. Biostimulants in horticulture. Sci Hortic. 2015;196: 1–2. doi:10.1016/j.scienta.2015.10.044

31. Calvo P, Nelson L, Kloepper JW. Agricultural uses of plant biostimulants. Plant Soil. 2014;383: 3–41. doi:10.1007/s11104-014-2131-8

32. du Jardin P. Plant biostimulants: Definition, concept, main categories and regulation. Sci Hortic. 2015;196: 3–14. doi:10.1016/j.scienta.2015.09.021

33. Yakhin OI, Lubyanov AA, Yakhin IA, Brown PH. Biostimulants in plant science: A global perspective. Frontiers in Plant Science. Frontiers Media S.A.; 2017. doi:10.3389/fpls.2016.02049

34. Zhang L, Liao W, Huang Y, Wen Y, Chu Y, Zhao C. Global seaweed farming and processing in the past 20 years. Food Production, Processing and Nutrition. 2022;4: 23. doi:10.1186/s43014-022-00103-2

35. Battacharyya D, Babgohari MZ, Rathor P, Prithiviraj B. Seaweed extracts as biostimulants in horticulture. Sci Hortic. 2015;196: 39–48. doi:10.1016/j.scienta.2015.09.012

36. Zarraonaindia I, Cretazzo E, Mena-Petite A, Díez-Navajas AM, Pérez-López U, Lacuesta M, et al. Holistic understanding of the response of grapevines to foliar application of seaweed extracts. Front Plant Sci. 2023;14: 1119854. doi:10.3389/FPLS.2023.1119854/BIBTEX

37. Lötze E, Hoffman EW. Nutrient composition and content of various biological active compounds of three South African-based commercial seaweed biostimulants. J Appl Phycol. 2016;28: 1379–1386. doi:10.1007/s10811-015-0644-z

38. Stirk WA, Van Staden J. Comparison of cytokinin-and auxin-like activity in some commercially used seaweed extracts. J Appl Phycol. Kluwer Academic Publishers; 1997.

39. Stirk WA, Tarkowská D, Turečová V, Strnad M, van Staden J. Abscisic acid, gibberellins and brassinosteroids in Kelpak®, a commercial seaweed extract made from *Ecklonia maxima*. J Appl Phycol. 2014;26: 561–567. doi:10.1007/s10811-013-0062-z

40. Khan W, Rayirath UP, Subramanian S, Jithesh MN, Rayorath P, Hodges DM, et al. Seaweed Extracts as Biostimulants of Plant Growth and Development. J Plant Growth Regul. 2009;28: 386–399. doi:10.1007/s00344-009-9103-x

41. Craigie JS. Seaweed extract stimuli in plant science and agriculture. J Appl Phycol. 2011;23: 371–393. doi:10.1007/s10811-010-9560-4

42. Khan AS, Ahmad B, Jaskani MJ, Ahmad R, Malik AU. Foliar Application of Mixture of Amino Acids and Seaweed (*Ascophylum nodosum*) Extract Improve Growth and Physico-chemical Properties of Grapes. Int J Agric Biol. 2012;14: 383–388. Available: https://www.cabidigitallibrary.org/doi/pdf/10.5555/20123198898

43. Lukács AF, Kovács GM. Effect of aboveground plant conditioner treatment on arbuscular mycorrhizal colonization of tomato and pepper. Horticultural Science. 2019;46: 208–214. doi:10.17221/4/2019-HORTSCI

44. Samuels LJ, Setati ME, Blancquaert EH. Towards a Better Understanding of the Potential Benefits of Seaweed Based Biostimulants in *Vitis vinifera* L. Cultivars. Plants. 2022;11: 348. doi:10.3390/plants11030348

45. Sharma HSS, Fleming C, Selby C, Rao JR, Martin T. Plant biostimulants: a review on the processing of macroalgae and use of extracts for crop management to reduce abiotic and biotic stresses. J Appl Phycol. 2014;26: 465–490. doi:10.1007/s10811-013-0101-9

46. Shukla PS, Borza T, Critchley AT, Prithiviraj B. Seaweed-Based Compounds and Products for Sustainable Protection against Plant Pathogens. Mar Drugs. 2021;19: 59. doi:10.3390/md19020059

47. Shukla PS, Mantin EG, Adil M, Bajpai S, Critchley AT, Prithiviraj B. *Ascophyllum nodosum*-based Biostimulants: Sustainable Applications in Agriculture for the Stimulation of Plant Growth, Stress Tolerance, and Disease Management. Front Plant Sci. 2019;10. doi:10.3389/fpls.2019.00655

48. Arioli T, Mattner SW, Hepworth G, McClintock D, McClinock R. Effect of seaweed extract application on wine grape yield in Australia. J Appl Phycol. 2021;33: 1883–1891. doi:10.1007/s10811-021-02423-1

49. Petoumenou DG, Patris V-E. Effects of Several Preharvest Canopy Applications on Yield and Quality of Table Grapes (*Vitis vinifera* L.) Cv. Crimson Seedless. Plants. 2021;10: 906. doi:10.3390/plants10050906

50. Vasconcelos Botelho R, Rusin C, Tumbarello G, Domenico Rombolà A. Yield and physicochemical characteristics of grapes from vines treated with extract of *Ecklonia maxima*. Pesquisa Aplicada & Agrotecnologia. 2018;11. doi:10.5935/PAeT.V11.N2.01

51. Coombe BG. Growth Stages of the Grapevine: Adoption of a system for identifying grapevine growth stages. Aust J Grape Wine Res. 1995;1: 104–110. doi:10.1111/j.1755-0238.1995.tb00086.x

52. Baskerville GL, Emin P. Rapid Estimation of Heat Accumulation from Maximum and Minimum Temperatures. Ecology. 1969;50: 514–517. doi:10.2307/1933912

53. Buck AL. New Equations for Computing Vapor Pressure and Enhancement Factor. Journal of Applied Meteorology. 1981;20: 1527–1532.

54. Schindelin J, Arganda-Carreras I, Frise E, Kaynig V, Longair M, Pietzsch T, et al. Fiji: an open-source platform for biological-image analysis. Nat Methods. 2012;9: 676–682. doi:10.1038/nmeth.2019

55. Chow PS, Landhausser SM. A method for routine measurements of total sugar and starch content in woody plant tissues. Tree Physiol. 2004;24: 1129–1136. doi:10.1093/treephys/24.10.1129

56. Smith JP, Holzapfel BP. Cumulative Responses of Semillon Grapevines to Late Season Perturbation of Carbohydrate Reserve Status. Am J Enol Vitic. 2009;60: 461–470. doi:10.5344/ajev.2009.60.4.461

57. Murchie EH, Lawson T. Chlorophyll fluorescence analysis: a guide to good practice and understanding some new applications. J Exp Bot. 2013;64: 3983–3998. doi:10.1093/jxb/ert208

58. Strasser RJ, Srivastava A, Govindjee. Polyphasic Chlorophyll *a* Fluorescence Transient in Plants and Cyanobacteria*. Photochem Photobiol. 1995;61: 32–42. doi:10.1111/j.1751-1097.1995.tb09240.x

59. Bates D, Mächler M, Bolker B, Walker S. Fitting Linear Mixed-Effects Models Using lme4. J Stat Softw. 2015;67: 1–48. doi:10.18637/jss.v067.i01

60. Lenth R V. emmeans: Estimated Marginal Means, aka Least-Squares Means. 2022. Available: https://CRAN.R-project.org/package=emmeans

61. Wickham H. ggplot2: Elegant Graphics for Data Analysis. 2nd ed. New York: Springer-Verlag; 2016. Available: https://ggplot2.tidyverse.org

62. R Core Team. R: A language and environment for statistical computing. R Foundation for Statistical Computing, Vienna, Austria. Vienna, Austria: R Foundation for Statistical Computing; 2021. Available: https://www.R-project.org/

63. Vivin P, Castelan M, Gaudillère JP. A source/sink model to simulate seasonal allocation of carbon in grapevine. Acta Horticulturae. International Society for Horticultural Science; 2002. pp. 43–56. doi:10.17660/ActaHortic.2002.584.4

64. Hugalde IP, Agüero CB, Barrios-Masias FH, Romero N, Viet Nguyen A, Riaz S, et al. Modeling vegetative vigour in grapevine: unraveling underlying mechanisms. Heliyon. 2020;6. doi:10.1016/j.heliyon.2020.e05708

65. Sabir A, Yazar K, Sabir F, Kara Z, Yazici MA, Goksu N. Vine growth, yield, berry quality attributes and leaf nutrient content of grapevines as influenced by seaweed extract (*Ascophyllum nodosum*) and nanosize fertilizer pulverizations. Sci Hortic. 2014;175: 1–8. doi:10.1016/j.scienta.2014.05.021

66. Chen L, Cheng L. Both xanthophyll cycle-dependent thermal dissipation and the antioxidant system are up-regulated in grape (*Vitis labrusca* L. cv. Concord) leaves in response to N limitation. J Exp Bot. 2003;54: 2165–2175. doi:10.1093/jxb/erg220

67. Keller M. Deficit Irrigation and Vine Mineral Nutrition. Am J Enol Vitic. 2005;56: 267–283. doi:10.5344/ajev.2005.56.3.267

68. Keller M. The Science of Grapevines. 3rd ed. Connors K, editor. Prosser, WA: Elsevier; 2020. doi:10.1016/C2013-0-06797-7

69. Sade N, Gebremedhin A, Moshelion M. Risk-taking plants Anisohydric behavior as a stress-resistance trait. Plant Signal Behav. 2012;7: 767–770. doi:https://doi.org/10.4161/psb.20505

70. Gutiérrez-Gamboa G, Pérez-Donoso AG, Pou-Mir A, Acevedo-Opazo C, Valdés-Gómez H. Hydric behaviour and gas exchange in different grapevine varieties (*Vitis vinifera* L.) from the Maule Valley (Chile). South African Journal of Enology and Viticulture. 2019;40. doi:10.21548/42-2-3224

71. Wilberts L, Rojas-Preciado N, Jacquemyn H, Lievens B. Fungal strain and crop cultivar affect growth of sweet pepper plants after root inoculation with entomopathogenic fungi. Front Plant Sci. 2023;14. doi:10.3389/fpls.2023.1196765

72. Auzmendi I, Holzapfel BP. Leaf area to fruit weight ratios for maximising grape berry weight, sugar concentration and anthocyanin content during ripening. Acta Hortic. 2016;1115: 127–132. doi:10.17660/ACTAHORTIC.2016.1115.19

73. Keller M, Romero P, Gohil H, Smithyman RP, Riley WR, Casassa LF, et al. Deficit Irrigation Alters Grapevine Growth, Physiology, and Fruit Microclimate. Am J Enol Vitic. 2016;67: 426–435. doi:10.5344/AJEV.2016.16032

74. Norrie J, Keathley JP. Benefits of *Ascophyllum nodosum* Marine-plant Extract Applications to ´Thompson Seedless´ Grape Production. Acta Hortic. 2006; 243–248. doi:10.17660/ActaHortic.2006.727.27

75. Frioni T, Sabbatini P, Tombesi S, Norrie J, Poni S, Gatti M, et al. Effects of a biostimulant derived from the brown seaweed *Ascophyllum nodosum* on ripening dynamics and fruit quality of grapevines. Sci Hortic. 2018;232: 97–106. doi:10.1016/j.scienta.2017.12.054

76. Frioni T, Tombesi S, Quaglia M, Calderini O, Moretti C, Poni S, et al. Metabolic and transcriptional changes associated with the use of *Ascophyllum nodosum* extracts as tools to improve the quality of wine grapes (*Vitis vinifera* cv. Sangiovese) and their tolerance to biotic stress. J Sci Food Agric. 2019;99: 6350–6363. doi:10.1002/jsfa.9913

77. Taskos DG, Stamatiadis S, Yvin J-C, Jamois F. Effects of an *Ascophyllum nodosum* (L.) Le Jol. extract on grapevine yield and berry composition of a Merlot vineyard. Sci Hortic. 2019;250: 27–32. doi:10.1016/j.scienta.2019.02.030

78. Salvi L, Brunetti C, Cataldo E, Storchi P, Mattii GB. Eco-Physiological Traits and Phenylpropanoid Profiling on Potted *Vitis vinifera* L. cv Pinot Noir Subjected to *Ascophyllum nodosum* Treatments under Post-Veraison Low Water Availability. Applied Sciences. 2020;10: 4473. doi:10.3390/app10134473

79. Salvi L, Brunetti C, Cataldo E, Niccolai A, Centritto M, Ferrini F, et al. Effects of *Ascophyllum nodosum* extract on *Vitis vinifera*: Consequences on plant physiology, grape quality and secondary metabolism. Plant Physiology and Biochemistry. 2019;139: 21–32. doi:10.1016/j.plaphy.2019.03.002

80. Conradie WJ. Seasonal Uptake of Nutrients by Chenin Blanc in Sand Culture: II. Phosphorus, Potassium, Calcium and Magnesium. South African Journal of Enology & Viticulture. 1981;2. doi:10.21548/2-1-2403

81. Laurent C, Oger B, Taylor JA, Scholasch T, Metay A, Tisseyre B. A review of the issues, methods and perspectives for yield estimation, prediction and forecasting in viticulture. European Journal of Agronomy. 2021;130: 126339. doi:10.1016/J.EJA.2021.126339

82. Vaillant-Gaveau N, Wojnarowiez G, Petit A-N, Jacquens L, Panigai L, Clement C, et al. Relationships between carbohydrates and reproductive development in chardonnay grapevine: impact of defoliation and fruit removal treatments during four successive growing seasons. OENO One. 2014;48: 219. doi:10.20870/oeno-one.2014.48.4.1694

83. de Carvalho RP, Pasqual M, de Oliveira Silveira HR, de Melo PC, Bispo DFA, Laredo RR, et al. "Niágara Rosada" table grape cultivated with seaweed extracts: physiological, nutritional, and yielding behavior. J Appl Phycol. 2019;31: 2053–2064. doi:10.1007/s10811-018-1724-7

84. National Geographic Institute. Orthophoto-based spatial map of Research Station for Fruit Growing [online map] [CC BY 4.0 (https://creativecommons.org/licenses/by/4.0/)]. Brussels: National Geographic Institute; 2022. Available: https://www.geo.be/map?l=en&x=573829.12&y=6581454.45&zoom=19&topic=fed&baseLayer=ngi.cartoweb.topo_bw.be&catalogNodes=1536&layers=orthophoto

Supporting information

S1 Fig. (A) Overview of the vineyard layout and trial row location from orthophoto-based spatial map of Research Station for Fruit Growing. Source: National Geographic Institute [84]**. Licensed under CC BY 4.0 (https://creativecommons.org/licenses/by/4.0/), and (B) the pruning and canopy management schedule.**

S2 Fig. Overview of the spraying schedule (A), the experimental randomized block design (B) and the berry sampling schedule (C). Odd-numbered timepoints represent experimental treatments and even-numbered timepoints the application of a minimal intervention potassium bicarbonate-based pesticide Karma® (A). Note that samples were only taken from the three central vines of each five-vine treatment block to reduce the impact of carryover between treatments (indicated with a check mark). Grey panels and blocks were treated only with Karma® (C).

S3 Fig. Comparison of the maximum daily vapour pressure deficit (VPD) (A), and hourly VPD and temperature profiles for the reproductive growth stages of 2021 (B) and 2022 (C). VPD is shown as a dashed line and temperature as a solid line for both growing seasons (B and C).

S4 Fig. Average shoot length of three representative shoots per replicate vine (*n* = 12) in 2021 (A) and 2022 (B) of *V. vinifera* cv. Chardonnay treated with a water control, an *A. nodosum* extract, an *E. maxima* extract and an NPK-reference treatment.

S1 Table. Overview of the nutrient composition of the applied seaweed extracts in relation to the NPK-reference treatment.

S2 Table. Effects [a] of treatment [b], development stage (time) [c], season (2021 vs 2022) and their interaction [d] on the leaf anatomical and photosynthetic parameters over two growing seasons.

S3 Table. Average leaf area and leaf dry mass of *V. vinifera* cv. Chardonnay in response to treatment with water as control, an *A. nodosum* extract, an *E. maxima* extract and an NPK-reference treatment. Each value represents the mean ± standard error (n = 12). Within each development stage for each season, treatments that showed significantly different responses are indicated with different letters.

S4 Table. Average leaf photosystem II efficiency (F_v/F_m), leaf chlorophyll content index (CCI) and leaf stomatal conductance of *V. vinifera* cv. Chardonnay following treatment with water as control, an *A. nodosum* extract, an *E. maxima* extract and an NPK-reference treatment. Each value represents the mean ± standard error (n = 12). Within each development stage for each season, treatments that showed significantly different responses are indicated with different letters.

S5 Table. Effects [a] of treatment (four levels) [b], plant development stages (variable dependent) [c], growing season (2021 and 2022), and their interactions [d] on berry morphology and classical parameters of *V. vinifera* cv. Chardonnay.

S6 Table. Average berry volume and berry mass of *V. vinifera* cv. Chardonnay in response to treatment with water as control, an *A. nodosum* extract, an *E. maxima* extract and an NPK-reference treatment. Each value represents the mean ± standard error (n = 4). Within each development stage for each season, treatments that showed significantly different responses are indicated with different letters.

S7 Table. Average titratable acidity, soluble solids (°Brix) and sugar content per berry of *V. vinifera* cv. Chardonnay in response to treatment with water as control, an *A. nodosum* extract, an *E. maxima* extract and an NPK-reference treatment. Each value represents the mean ± standard error (n = 4). Within each development stage for each season, treatments that showed significantly different responses are indicated with different letters.

S1 Data. Data behind all results presented in this manuscript.

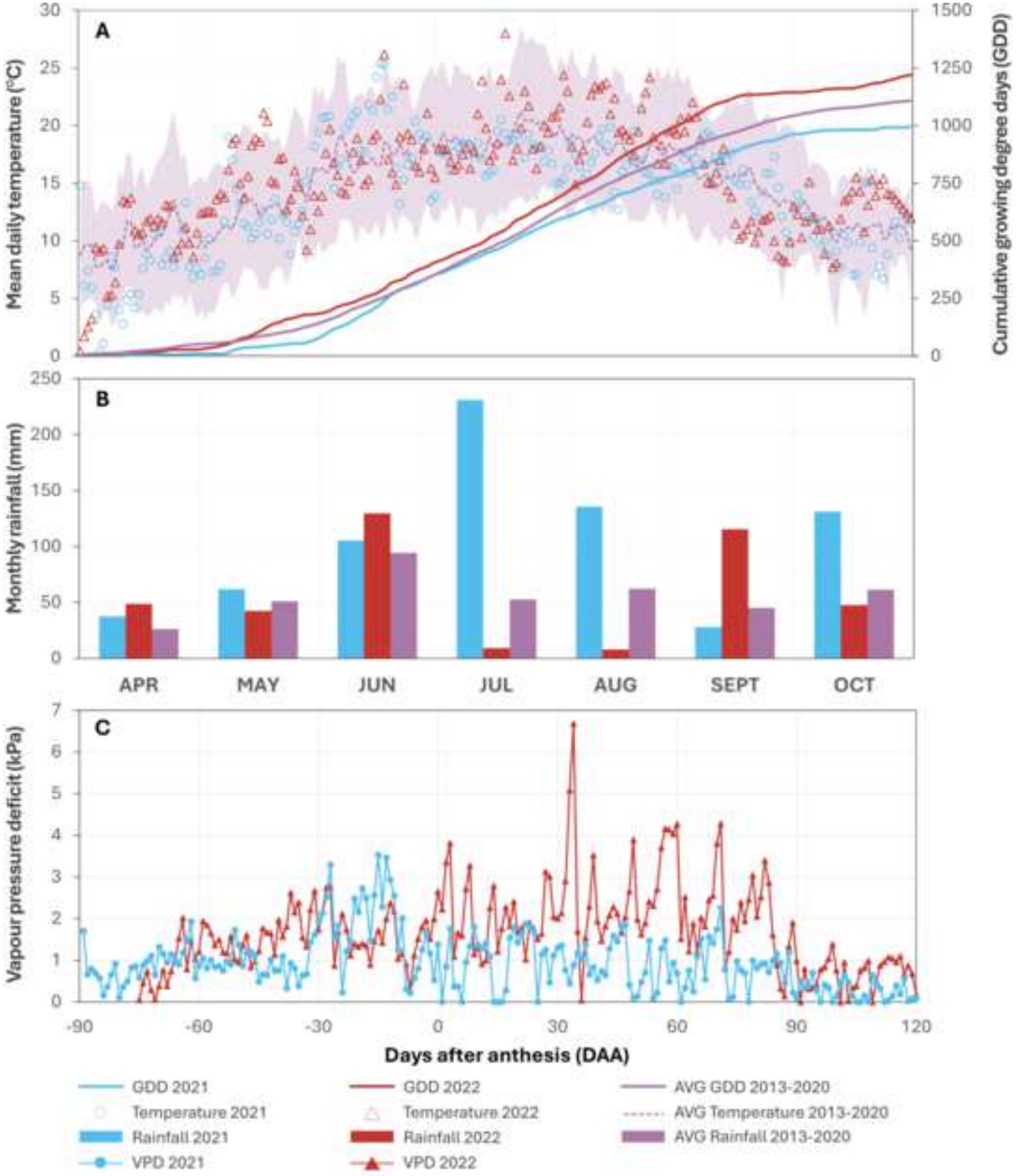
A
B
C
Mean daily temperature (°C)
Cumulative growing degree days (GDD)
Monthly rainfall (mm)
Vapour pressure deficit (kPa)
APR
MAY
JUN
JUL
AUG
SEPT
OCT
Days after anthesis (DAA)
GDD 2021
GDD 2022
AVG GDD 2013-2020
Temperature 2021
Temperature 2022
AVG Temperature 2013-2020
Rainfall 2021
Rainfall 2022
AVG Rainfall 2013-2020
VPD 2021
VPD 2022

Fig2

Click here to access/download;Figure;Fig2.tif

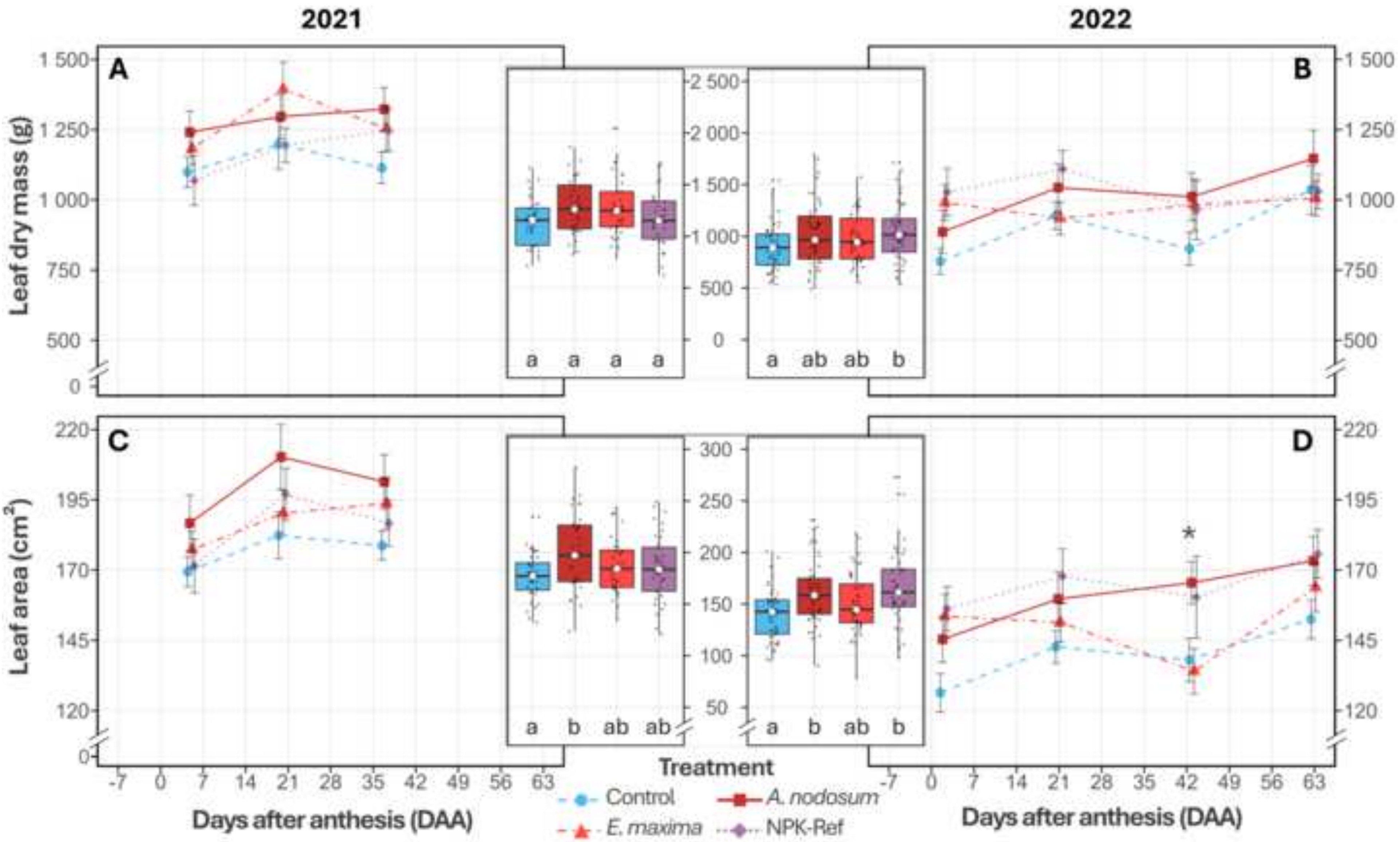

Click here to access/download;Figure;Fig3.tif

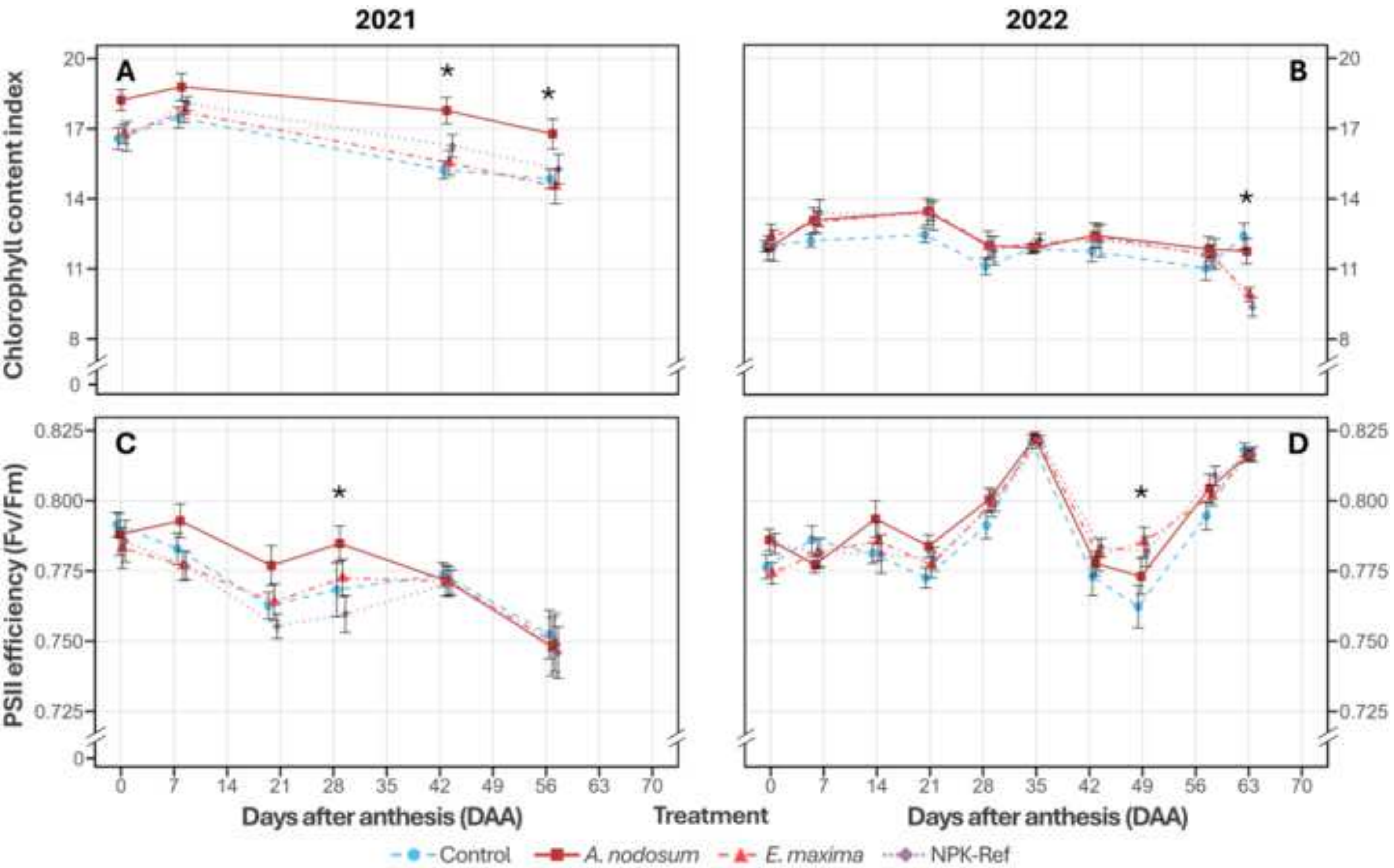

Click here to access/download;Figure;Fig4.tif

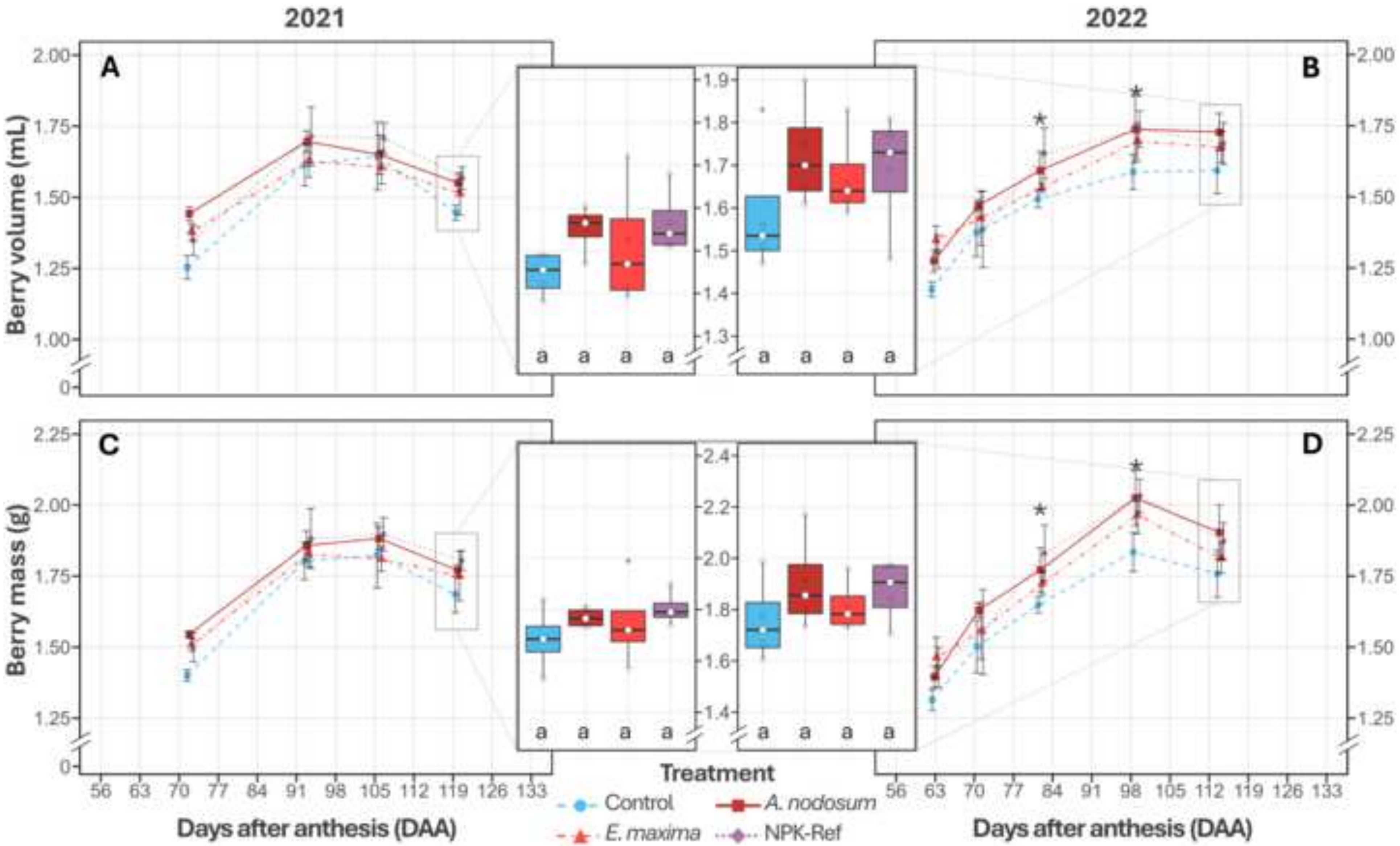

Fig5

Click here to access/download;Figure;Fig5.tif

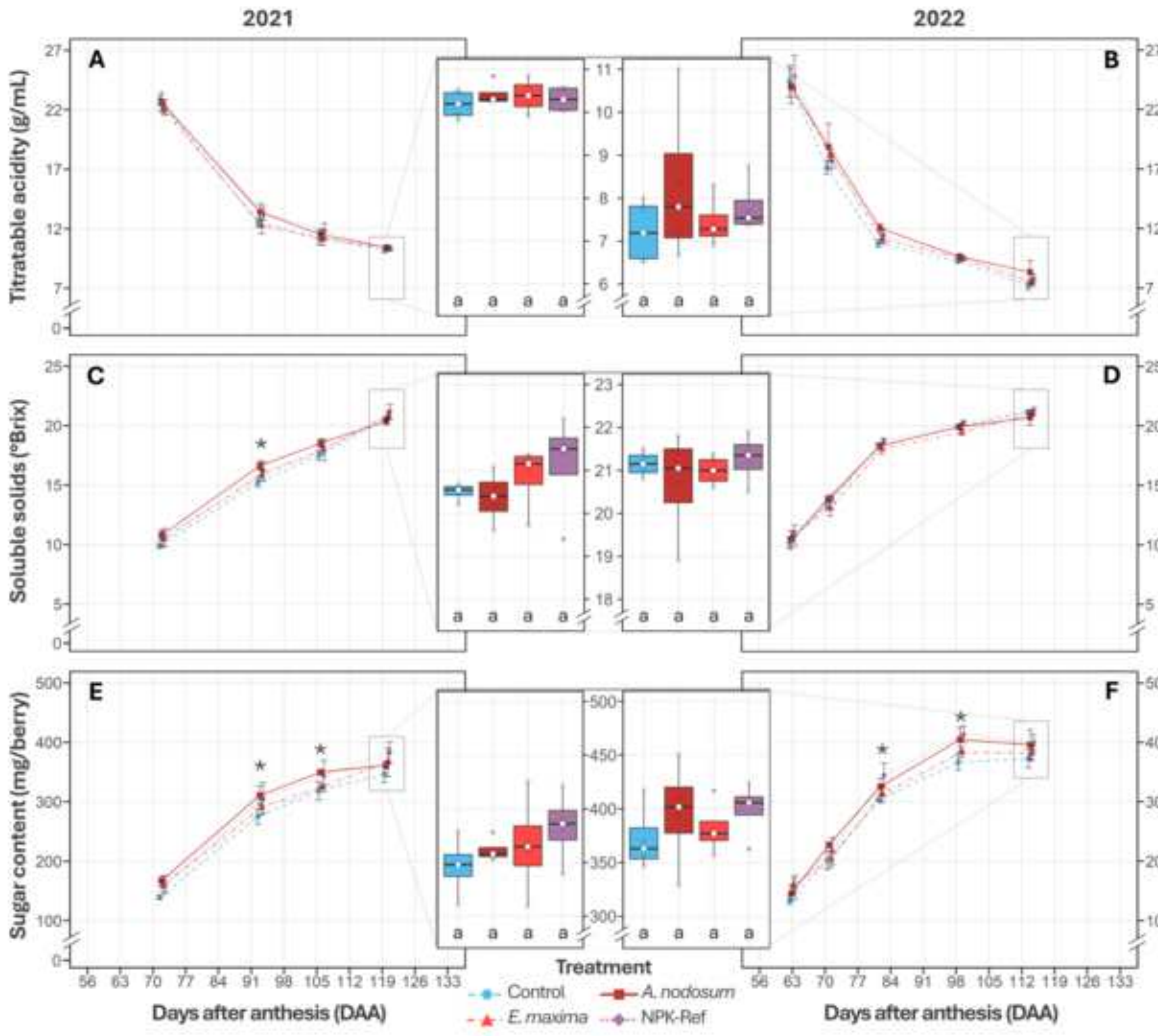

Fig6

Click here to access/download;Figure;Fig6.tif

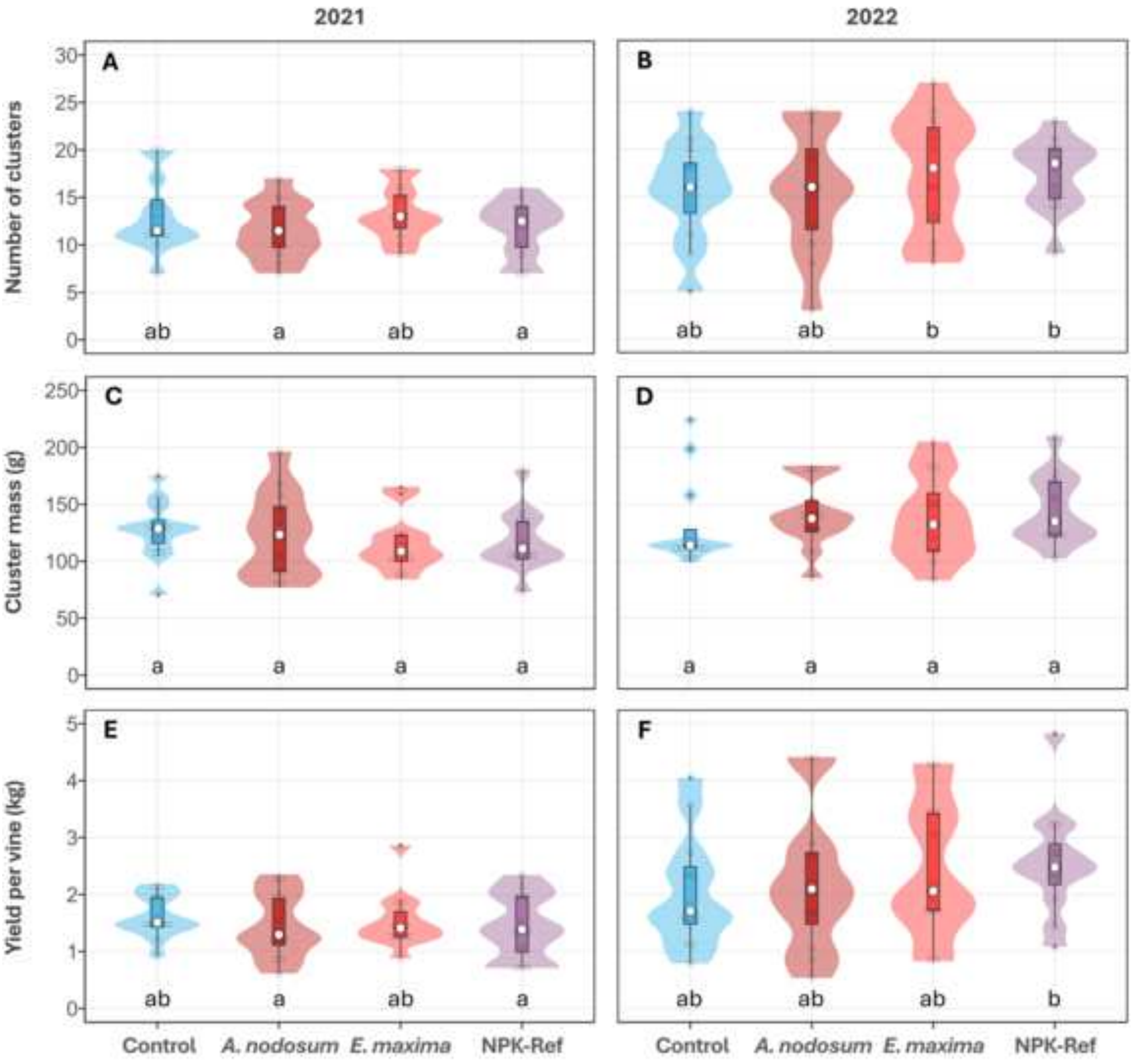

S1 Fig.

Click here to access/download
Supporting Information
S1_Fig.docx

S2 Fig.

Click here to access/download
Supporting Information
S2_Fig.docx

S3 Fig.

Click here to access/download
Supporting Information
S3_Fig.docx

S4 Fig.

Click here to access/download
Supporting Information
S4_Fig.docx

S1 Table.

Click here to access/download
Supporting Information
S1_Table.docx

S2 Table.

Click here to access/download
Supporting Information
S2_Table.docx

S3 Table.

Click here to access/download
Supporting Information
S3_Table.docx

S4 Table.

Click here to access/download
Supporting Information
S4_Table.docx

S5 Table.

Click here to access/download
Supporting Information
S5_Table.docx

S6 Table.

Click here to access/download
Supporting Information
S6_Table.docx

S7 Table.

Click here to access/download
Supporting Information
S7_Table.docx

Supplementary Data

Click here to access/download
Supporting Information
S1_Data.xlsx
