# Supplementary material for: Assessing the potential of seaweed extracts to improve vegetative, physiological and berry quality parameters in Vitis vinifera cv. Chardonnay under cool climatic conditions
Source: PLoS One. 2025 Sep 2;20(9):e0331039. doi: 10.1371/journal.pone.0331039 (PMC12404493; doi:10.1371/journal.pone.0331039)
Supplement: Attachment — Submitted filename: Reviewer1.pdf [file pone.0331039.s016.pdf]

Comments on the revised Manuscript

New Title: Assessing the potential of seaweed extracts to improve vegetative, physiological and berry quality parameters in *Vitis vinifera* cv. Chardonnay under cool climatic conditions
Manuscript ID: PONE-D-24-45452
Journal: PLOS ONE

Global comment:
The manuscript improved a lot, it's almost a whole new paper. The authors have addressed the vast majority of the comments of both reviewers very diligently and competently, with a special emphasis on Discussion and Statistics, where they did an enormous and great job. I have no doubt that it's adequate for publication in PLOS.
I still have some specific comments on few aspects that I'm sure will be easy to handle.

Specific comments (the referred lines are of the revised ms):

- Regarding the need for a better justification for focusing on cooler/moist regions and the eventual contribution of seaweed extracts on the mitigation of potential stresses, an important point raised by the editor and the reviewers, and addressed by the authors (lines 101-105), I still think that, although they indicated that "these regions are challenged by variable weather conditions as well as extreme weather events" (what about the hotter/drier regions?) and that grapevines may be "particularly vulnerable showing delayed ripening and increased disease incidence", they didn't explain and it's not clear "in what way seaweed extracts may help to overcome" those problems in particular (already on my previous specific comments - lines 81-83). The authors add that "seaweed may mitigate stress by regulating photosynthesis, enhancing WUE and nutrient uptake", but: i) regulating Pn in what sense? Increasing?; ii) is there a problem with WUE in cooler/more humid regions? I don't think so; iii) or with NU? And even if so, how to link an increase in WUE and NU with a less delayed ripening or less susceptibility to diseases? I call attention for the fact that ref. 47 does not address stress in cool/ humid regions, but mainly the benefits of *A. nodosum* extracts in plants under drought stress. Having said this, I think that the authors can accommodate these issues putting the tonic on that the addition of seaweeds extracts (namely *A. nodosum*) can "stimulate natural processes to enhance/benefit nutrient uptake, nutrient efficiency, tolerance to abiotic stresses (47)", and that grapevines well fertilized an in better physiological status are also better prepared to respond to biotic as well as abiotic challenges. All in all, it is important to clearly refer to the stresses/challenges that grapevine varieties can endure in cool/humid regions, so it would be reasonable to accept that the extracts could, in fact, help alleviating from/avoiding injury caused by the stress factors.
- Line 101 – add examples of "extreme events".
- Line 103 – rephrase, I cannot understand exactly what do you want to say with this sentence – i) what "stress conditions"?; ii) and "similar" to what?; iii) "experienced by grapevine varieties that are more adapted to these conditions", if by "these conditions" you mean cool/moist ones, how can varieties more adapted to those conditions experience stress?
- Line 126 – don't start a sentence with numerals or abbreviations – use *Ecklonia* in full.

- Lines 271 – reformulate adding what I've underligned - "xxx dark adapted for 30 min to open the RC and record minimal fluorescence (F0) and subject to xxx to obtain maximum fluorescence (Fm) and compute Fv/Fm. Fv/Fm =$(F_m-F_0)/F_m$
- Accordingly to the above comment, it is important to include the procedure to calculate Φ_{PSII} (=$(F'_m-F_s)/F'_m$). This parameter is measured in light-adapted plants/leaves, so the AL intensity and duration of the light adaptation period before another saturation pulse (or flash) is given is important to include.
- Line 424 – "were no significant differences in shoot length between xxx"
- Line 962 – don't use "photosynthetic capacity" because it has a proper meaning and you didn't measure/calculated this parameter - substitute by the parameters you've measured or say just "photochemical efficiency", what can be a general expression that may englobe Fv/Fm and Φ_{PSII}.
