# Supplementary material for: Assessing the potential of seaweed extracts to improve vegetative, physiological and berry quality parameters in Vitis vinifera cv. Chardonnay under cool climatic conditions
Source: PLoS One. 2025 Sep 2;20(9):e0331039. doi: 10.1371/journal.pone.0331039 (PMC12404493; doi:10.1371/journal.pone.0331039)
Supplement: Attachment — Submitted filename: Response to Reviewers.docx [file pone.0331039.s015.docx]

Leuven, Belgium, March 31, 2025

Dear Editor,

Please find herewith our revised manuscript ‘Assessing the potential of seaweed extracts to improve vegetative, physiological and berry quality parameters in *Vitis vinifera* cv. Chardonnay under cool climatic conditions’.

We thank the referees for their extensive and constructive comments and suggestions on the previous version of this manuscript (PONE-D-24-45452). Not only were the comments very useful when revising the text, but they also allowed the authors to gain additional insight into fields orthogonal to their research. Given the extensive nature of the comments, the rebuttal letter is structured as follows:

- Response to the global comments of the editor p. 2-3
- Summary of the major comments with the authors’ response or corrective action – in certain instances the authors refer to a detailed response, specified by line number in the Appendix of the rebuttal letter p. 3-9
  - Responses are also linked to lines in the **updated MS** as “**MS Lxxx-xxx**”.
- Shorter comments are addressed directly according to their respective suggestions (based on the line numbers in the *original MS*), and can be found in the Appendix at the end of the rebuttal letter, grouped by reviewer.

In addition to a clean version of the revised manuscript, a version with changes marked through Track Changes, has been uploaded.

We hope that you find this revised version suitable for publication in PLOS ONE and we look forward to receiving comments in due time.

Sincerely,

Prof. Dr. Ir. Sam Crauwels

CMPG Laboratory for Process Microbial Ecology and Bioinspirational Management (PME&BIM)

Department of Microbial and Molecular Systems (M2S), KU Leuven

Willem De Croylaan 46, B-3001 Leuven, Belgium

E-mail: [sam.crauwels@kuleuven.be](mailto:sam.crauwels@kuleuven.be)

# Editor comments

Thank you for submitting your manuscript to PLOS ONE. After careful consideration, we feel that it has merit but does not fully meet PLOS ONE’s publication criteria as it currently stands. Therefore, we invite you to submit a revised version of the manuscript that addresses the points raised during the review process. You will see that both reviewers were very positive about your study and provided highly detailed suggestions to help improve the manuscript. I agree that your study has merit and deserves publication in PLOS ONE after a substantial revision (without requiring additional experiments) throughout the text.

***Authors:*** *We thank the editor and reviewers for their constructive and extensive comments and suggestions, and appreciate the time and thoroughness with which they have evaluated the manuscript. Not only have the suggestions and comments made by the editor and reviewers been very helpful in improving the manuscript, but they also contributed to additional insight for the authors to fields orthogonal to their research field.*

*Given the extensive nature of the comments and suggestions, the authors first present a summary of the major comments combined with the author’s response or corrective action. Shorter comments are addressed directly according to their respective suggestions (based on the original line numbers in the MS), and can be found in the Appendix at the end of the rebuttal letter, grouped by reviewer.*

While the study compellingly explores the effects of seaweed extracts on grapevines in cool climates, the rationale for focusing on colder regions needs to be better articulated. Simply stating that cold climates represent "another stress factor" is insufficient to justify this focus, particularly as most of the existing literature emphasizes drought and heat stress as the primary targets for seaweed-based treatments. Please revise the introduction to provide a stronger justification.

***Authors:*** *In the revised introduction, we explicitly highlight several unique challenges faced by cooler growing regions. For instance, these regions not only experience variable weather and extreme events but are also particularly vulnerable to issues such as delayed ripening and increased disease incidence. This contrasts with the better‐studied drought and heat stress conditions which typically aid ripening. The authors highlight the scarcity of literature regarding biostimulant effects in these regions, in particular under variable climatic conditions and thus provides a stronger justification for exploring seaweed extract effects in cool climates.*

Additionally, as highlighted by Reviewer #2, although no direct molecular investigations were performed, discussing the potential mechanisms underlying the observed effects—such as phytohormone activity, stress-response pathways, or enhanced nutrient uptake—would greatly enhance the depth of the study and provide a foundation for future research. Furthermore, it would be valuable to explore how these mechanisms might overlap with or differ from those involved in protection against drought or heat stress, as this comparison could offer new insights into the broader applicability of seaweed extracts in stress mitigation.

***Authors:*** *The authors agree that the original discussion lacked depth. Therefore, the section has been substantially revised to provide possible explanations for the observed effects, focussing more extensively on seaweed extract composition (nutrients as reported, phytohormones and bioactive compounds based on literature) and, as suggested by the editor, also the interplay of plant responses to the specific climatic conditions that were observed. A summary of changes is reported under ‘Major comments’, with details provided in the Appendix.*

The term "reproductive parameters" in the title might not be entirely accurate, especially in light of the data and discussion presented. More precise terms such as fruit development, berry attributes, or berry traits would better reflect the study's focus. Please consider revising the title accordingly.

***Authors:*** *We agree with the editor and the reviewer on this matter and have updated the title to instead refer to ‘berry quality parameters’. Furthermore, the terms ‘berry development’ were also used in the manuscript in lieu of reproductive parameters.*

As also suggested by the reviewers, the manuscript would benefit from being less descriptive. For instance, you might consider removing the first sentence of the discussion (line 489) and the sentence in line 292. Additionally, please address the remaining comments and suggestions from the reviewers, as they provide relevant and valuable insights for improving the manuscript.

***Authors:*** *The authors agree with the comments made by the editor and the reviewers, the manuscript has been updated as outlined below under Major comments, with additional details provided and minor corrections addressed in the Appendix, grouped per reviewer.*

# Major comments

## Main changes

- **Introduction:** Strengthened rationale for cool-climate study, expanded on possible benefits of seaweed extracts and their variability in terms of composition, and provided additional support regarding the relevant issue of sustainability of harvesting *Ecklonia maxima*.
- **Materials and Methods:** Clarified relevant aspects of study design. Major revision of the explanation of the statistical method, with two additional parameters included (from already generated fluorescent transient data), and additional statistical diagnostic checks to ensure robustness.
- **Results:** Analyses rerun according to streamlined statistical method to ensure robustness. Figures received major overhaul to better accommodate reviewer recommendations (e.g. violin-boxplot inlay added for complete season’s fluorescence measurement data). Figure and table captions and footnotes harmonised, as well as Supplementary figures and tables.
- **Discussion**: Major revisions to structure, first results are contextualised within the context of similar studies, keeping climatic differences in mind. Next, possible mechanisms are explored relating to composition, more specifically examining possible mechanisms based on quantified nutrients, as well as potential mechanisms based on literature reported phytohormones and bio-active compounds. Study limitations are outline more clearly and extensively, followed by a more in depth exploration of potential future studies.
- **Conclusion**: The conclusion has been updated to be more clear and structured, first defining the study, reviewing main results and contextualising it within literature, followed by a closing statement highlighting that the study demonstrates the potential of seaweed extracts to replace or serve as adjuncts to NPK foliar treatments.
- **References**: References have been migrated from Mendeley to Zotero, with care taken to conform to PLOS ONE’s guidelines.
- **Figures and Tables**: Recreated based on reviewer recommendations, with updated Figure elements (violin-boxplots showing data variation within seasons, application timepoints and shaded zones indicating specific climate conditions) and more harmonised captions and footnotes.

## Abstract

- **Clarity and speculative statements**
  - Suggested edits for clarity made according to individual advice in Appendix
  - Statements that are speculative were removed, reworked into discussion as potential mechanisms, based on literature.
- **Wordcount increased from 235 to 314**

## Introduction

- **Impact and Variability of Seaweed Extracts**
  - Expanded the discussion on the benefits of seaweed extracts by referring to two key variables influenced by seaweed extract [1,2] and clarifying the inherent variability in nutrient content and seasonal performance [3,4].
  - **MS L85-87**
  - *(Reviewer 1: Line 79 & Reviewer 2: Lines 83-85)*
- **Contextualizing Effects in Cool/Moist Regions**
  - Strengthened the rationale for studying cooler/more humid regions by detailing how extreme weather, increased disease pressure, and delayed ripening (due to climate change) can influence vineyard performance [5].
  - **MS L91-96**
  - *(Reviewer 1: Lines 81-83; Reviewer 2: Lines 43-45)*
- **Citing Regional Studies and Climatic Monitoring**
  - Provided detailed climate context and application methods using an illustrative Australian study [6], while also noting that climatic monitoring (temperature, precipitation) was performed and elaborated in the Materials and Methods section.
  - **MS L109-111; L178-188**
  - *(Reviewer 1: Line 94; Reviewer 2: Line 102-103)*
- **Sustainability of Ecklonia maxima**
  - Addressed concerns about the sustainable harvest of *E. maxima* by highlighting its recent range expansion in South Africa and its broad thermal tolerance, reinforcing its relevance as a biostimulant source [7–9].
  - **MS L114-117**
  - *(Reviewer 1: Lines 94-98)*
- ***Wordcount increased from 903 to 1122***

## Materials and Methods

- **Treatment Details and Inter-Annual Variations**
  - Clarified treatment compositions by stating that all treatments were commercial products—with the control comprising demineralised water and Tween‑20 (no additional compounds)—and provided product details in S1 Fig.
  - Explained inter-annual differences in treatment application timepoints as a response to variable climatic conditions that affect crop development and the risk of treatment wash-off by rain.
  - **MS L143-151; L159-165**
  - *(Reviewer 1: Reviewer 2: Lines 120–124, 133–136)*
- **Experimental Design & Vine Vegetative Characteristics**
  - Specified time interval for assessing vegetative characteristics, detailed calculation for specific leaf area (SLA).
  - Clarified that measurements were conducted on each biological replicate vine for vegetative/physiological and harvest parameters (*n* = 12), and each replicate panel (*n* = 4) for berry ripening and quality parameters.
  - **MS L191-192, 198-199, 258-260; L207; L199-200, 264-269**
  - *(Reviewer 1: Line 172-181, 181)*
- **Chlorophyll Indices and Stomatal Conductance**
  - Provided explicit calculations and definitions for key parameters:
    - **CCI:** Described as a unitless transmittance-based measure reflecting the relative chlorophyll concentration per unit leaf area.
    - **Fluorescence transients:** Clarified measurement procedure (using Pocket PEA fluorimeter) and which parameters were derived from the transients:
      - **RC/CS:** Reaction centre density per excited cross section.
      - **Fv/Fm:** The maximum quantum yield of photosystem II
      - **ФPSII:** Effective quantum yield of photosystem II.
    - Emphasized that dark-adaptation was required to obtain the maximum *possible* F_v_/F_m_, otherwise the measured F_v_/F_m_ is underestimated.
  - **MS L242-245; 246-250; L252-254**
  - *(Reviewer 1: Lines 214–222)*
- **Berry Parameters and Morphology**
  - Detailed berry sampling and processing methods: in Appendix p. 11 lines 187-196
  - **MS L262-289**
  - *(Reviewer 1: Lines 231, 239, 253)*
- **Statistical Analysis Overview**
  - Noted that the statistical analysis was rewritten for enhanced clarity and completeness.
  - Analyses were redone following the updated workflow which includes a more robust model selection step in addition to diagnostic checks. While the core results remained consistent, some borderline effects changed to non-significant following additional checks for model assumptions.
  - **A proof of concept for the parameter RC/CS is found after the appendix on p. 30**
  - Briefly we analyzed longitudinal vegetative and berry quality data using linear mixed models (LMMs) at a significance level of α = 0.05. For the vegetative dataset, a global model was built with fixed effects for season, treatment, time (and their interactions) plus environmental covariates, and with replicate vines nested within blocks as random effects to account for repeated measures. Candidate models were compared using AICc to select the most parsimonious model, with model assumptions checked and adjusted as necessary. Final models were refitted using REML, and significance was assessed via Type III ANOVA followed by post hoc pairwise comparisons using estimated marginal means.
  - A similar approach was used for the berry quality data, with time modelled as a 2nd order polynomial to capture ripening dynamics. Continuous responses were analysed using LMMs, while count data were modelled with a negative binomial GLM. Seasonal analyses and planned pairwise contrasts were conducted, with multiple comparisons adjusted accordingly.
  - Standardized abbreviations (e.g., using ANOVA) to ensure consistency throughout the manuscript.
  - **MS L299-355**
  - *(Reviewer 1: Lines 278, 282; Reviewer 2: Line 294)*
- ***Wordcount increased from 2657 to 3273 words***

## Results

**Grapevine Vegetative Growth & Physiology**

- **Fluorescence Parameters:**
  - Standardized the term “maximum quantum yield of photosystem II” for Fv/Fm and added the effective quantum yield (Ф_PSII_) as an additional parameter.
  - *(Reviewer 1: L355; also addressed in L359–360 by emphasizing treatment was not significant for stomatal conductance)*
  - Added extra fluorescence-derived parameters (RC/CS and ФPSII) to support observations:
    - Reduced CCI in 2022 is correlated with downregulation of PSII reaction centres under abundant light/warm conditions.
    - The decay in Fv/Fm in 2021 is attributed to moderate temperatures, high humidity, and increased downy mildew infections in Discussion.
  - *(Reviewer 1: L355–383; Figure 3 comments)*
- **Data Presentation Enhancements:**
  - Incorporated time (DAA and E-L stage) columns in Supplementary Table S4 and highlighted high VPD stress periods in Figure 3 to improve data interpretability.
  - *(Reviewer 1: L376–383)*

**Berry Development & Quality**

- **Terminology and Parameter Reporting:**
  - Updated “reproductive” to “berry development” or “berry quality” to accurately reflect the measured parameters.
  - *(Reviewer 1: L390)*
  - Revised descriptions in S5 Table to clearly state that Season and Treatment significantly affected berry volume, mass, density, and DM%, with significant Season × Time and VPD interactions on some parameters.
  - *(Reviewer 1: L391–392)*
- **Averaging vs. Harvest Timepoints:**
  - Defended averaging berry metrics over the ripening period to capture maturation dynamics, while also reporting final harvest values (Figure 4 and 5) to relate directly to yield and quality.
  - *(Reviewer 1: L401 and related discussion)*
  - Emphasized that the most meaningful comparisons are between seaweed extract treatments and the NPK-reference rather than to the water control.
    *(Reviewer 1: L399–408)*
- **Figure Presentation & Statistical Analysis**
  - **Figures:**
  - Redesigned figures to combine time-series plots with inlay violin-boxplots, using uppercase significance letters for season-averaged treatment effects and lowercase letters for harvest timepoints.
  - *(Reviewer 2: Comments on figure organization, L384, L400–408)*
  - Removed asterisks from time-series figures and instead used them only for indicating overall significance for Treatment, Time, or their interaction.
  - *(Reviewer 2: L400–408)*
  - **Statistical Approach:**
  - Revised the statistical analysis to clarify that ANOVAs were conducted on linear mixed models (LMMs) with model selection based on AICc. Where interactions were not significant, further two-way ANOVAs were applied.
  - *(Reviewer 2: L338, and additional comments on statistical procedures)*
  - Corrected a formulaic error that had misassigned CCI data for a specific timepoint in Figure 3B (L366–368) so that the data now consistently reflect seasonal trends.
  - *(Reviewer 2: L366–368)*
- **Harvest Parameters**
  - Minor typographical corrections were made to ensure consistency in the final harvest data presentation.
  - *(Reviewer 1: L466)*
- ***Wordcount increased from 3205 to 4161 words***

## Discussion

- **Global: Lacking depth/mechanistic insight beyond nutrient profile, explanation regarding nutrient discrepancies lacking.**
  - Expanded the discussion on the benefits of seaweed extracts by referencing two key biostimulant variables and clarifying the inherent variability in nutrient content and seasonal performance.
  - **Detailed response Appendix Rev1 Line 314-323 ; Rev2 Line 509-515**
  - *(Reviewer 1: Lines 534-536 & Reviewer 2: Global comment)*
- **Usage of Fluorescence Parameters Fv/Fm vs ФPSII as stress indicators & CCI definition and discussion**
  - We now include effective quantum yield (ФPSII) data (along with Fv/Fm) and additional RC/CS data to better reflect photosynthetic performance.
  - Chlorophyll Content Index (CCI) specified as transmittance based measure of relative chlorophyll concentration per unit leaf area in the Methods and Results sections by (Parry et al., 2014).
  - **Detailed response Appendix Lines 357-368**
  - *(Reviewer 1: Line 499)*
- **Biological Relevance of 1.5% Fv/Fm Increase**
  - In the context of controlled plant stress experiments it’s not relevant, where stressed vines typically have a 30-50% reduction in Fv/Fm. However in our study it did represent a sustained small-medium effect size using Cohen’s *d* standardized effect size (small = 0.2, medium = 0.5, large = 0.8) over the season with Cohen’s *d* = 0.42. Compare this to the medium to large effect size of the CCI for 2021 with Cohen’s *d* = 0.65.
  - **Detailed response Appendix Lines 730-734**
  - *(Reviewer 2: Line 502)*
- **Vegetative Growth Consistency: Reframe statements to align with Figure 2, noting that while vegetative growth was greater in 2021, the focus should be on fluorescence and CCI trends.**
- The authors note that Figure 2 refers to leaf area and mass, both which were higher in 2021, however the statement refers to fluorescence parameters and CCI per unit measured leaf area, where the following was observed:
  - Reduced CCI from 2021 to 2022 in addition to a trend of decreasing CCI values as the season progresses. The significant reduction in CCI from 2021 to 2022 is likely a response to regulate against potential photosystem damage resulting from high temperatures and light.
  - **Detailed response Appendix Lines 371-377**
  - *(Reviewer 1: Line 502-503)*
- **Why is there a reduction in chlorophyll content and an increase in photochemistry processes, which, in the first place, depend on chlorophyll?**
  - We acknowledge the seeming contradiction between reduced chlorophyll content and increased photochemical efficiency.
  - Under high irradiance, plants reduce chlorophyll (and RC/CS) to avoid overexcitation and photodamage.
  - Despite lower chlorophyll levels, efficiency per reaction centre improves (higher Fv/Fm and ΦPSII), likely due to enhanced enzymatic activity and PSII repair mechanisms
  - **Detailed response Appendix Lines 737-744**
  - *(Reviewer 2: Lines 502-505)*
- **L514 Water Stress Considerations – rephrase and clarify lack of water stress measurements**
  - Added details on lack of water stress due to high water table from previous season’s rain, and clarified that proposed effect of anisohydric characteristic was not explicitly confirmed as leaf water potentials were not measured.
  - **Detailed response Appendix Lines 387-389**
  - *(Reviewer 1: Line 514)*
- **Highlight needs for extract application optimisation (type, frequency, concentration, and method)**
  - For this study, the authors chose to focus on different species at supplier recommended concentrations, however Discussion expands on several aspects that can be optimised in future studies.
  - Additionally lixiviation/leaching was not considered a problem in this study as application were done in periods that were free of rain at least 24h post-application.
  - **Detailed response Appendix Lines 394-401**
  - *(Reviewer 1: Line 521)*
- **Ensure more objective comparison is made with NPK-Ref and discuss whether yield is improved with NPK.**
  - Corrected as suggested, MS makes clear mention where treatments did not significantly differ from NPK-Ref, thereby acting as possible substitute.
  - Yield not significantly different within 2022 season, only between season contrasts were evaluated.
  - **Detailed response Appendix Rev1 Lines 445-450; Rev2 Lines 760-769**
  - *(Reviewer 1: L577-579 & Reviewer 2 Lines 558-559)*
- **Contribution of Other Bioactive Components and secondary metabolites**
  - Expanded discussion on the nutrient profile differences between extracts (e.g., *A. nodosum* is much richer in several nutrients than *E. maxima*) to explain observed performance variations (L534–539).
  - Differences were based on measured composition, as well as proposed composition based on literature, in order to add depth to the discussion.
  - **Detailed response Appendix Rev1 Lines 426-431; Rev2 Lines 779-782**
  - *(Reviewer 1: L564-568 & Reviewer 2:Lines 581-582)*
- ***Wordcount increased from 1193 to 1693 words***

## Conclusion

- **More objective comparison with NPK Reference.**
  - Adjusted language to state comparable performance rather than implying superiority and clarified that *A. nodosum* achieved comparable effects to NPK-Ref.
- **Evaluation of *E. maxima***
  - Noted potential for heat stress recovery with a call for further studies and reported its yield improvement relative to the NPK-Ref treatment.
- **Hormonal Profile Emphasis:**
  - Added brief discussion on differing hormone profiles (cytokinin dominance in *A. nodosum* vs. auxin dominance in *E. maxima*) as a contributing factor.
- **Practical Implications:**
  - Expanded the conclusion to highlight seaweed extracts as a potential substitute or adjunct to synthetic NPK foliar fertilization, stressing the importance of understanding extract composition.
- **Detailed response Appendix Rev1 Lines 445-450; Rev2 Lines 779-782**
- *(Reviewer 1: Lines 534-536 & Reviewer 2: Global comment)*
- ***Wordcount increased from 140 to 251***

***Total wordcount increased from 8333 to 10814***

- *Main drivers were additional details in discussion, additional fluorescence parameters and more extensive statistical methods description, additional results as well as expanded discussion to include potential mechanisms for observed effects, based on literature.*

# Appendix

# Reviewer 1

Comments on the Manuscript

Title: Assessing the potential of seaweed extracts to improve vegetative, physiological and reproductive characteristics in Vitis vinifera cv. Chardonnay under cool climatic conditions

Manuscript ID: PONE-D-24-45452

Journal: PLOS ONE

Mail goal – to study the impact of foliar application of extracts from two algae species – Ascophyllum nodosum and Ecklonia maxima - as biostimulants on the performance of Chardonnay cultivar under cool-climate conditions, as those find in Belgium (where the experimental site is), as an opportunity to explore sustainable viticultural bio-based practices in new emerging/expanding vineyard regions.

Both brown algae (kelp) abundant in cold waters - A. nodosum from the North Sea to the north occidental European coast and E. maxima mostly in the southern Atlantic coast of Africa, from the south of South Africa to Namibia – and already used for food and feed and as agricultural supplement.

## General comments:

Globally this work does not constitute cutting edge research but:

- It focusses on an interesting and pertinent issue – viticultural practices based on natural sustainable inputs to improve yield and quality, proposing the use of marine algal extracts.
- It is very well written; the Introduction is well supported and M&M are thoroughly described and the field trials carefully planned and conducted.
- The amount and quality of the results are very good.
- It has a good discussion section.

***Authors:*** *The authors would like to thank the reviewer wholeheartedly for their kind words regarding the quality of the study design and the description of the methodology and results. That said the authors also acknowledge the points of attention that were raised by both reviewers and the editor, in addition to that arose from working through the revisions. The amount and quality of the comments have made this process rewarding and the authors have gained new insights into fields orthogonal to their research.*

However,

The Introduction should be much more informative in some aspects, such as giving evidence/refs regarding macro- and micronutrients/extract components uptake by the leaves (as they test the treatments by foliar application); also, attention with the harvesting of seaweed material was not approach and is important, specially knowing that one of the species (E. maxima) is now under pressure due to CC.

***Authors:*** *The authors have addressed the major comments for Introduction as outlined previously, further details are provided in the Appendix.*

The Material and Methods section should provide more detailed information here and there (see more in Specific comments).

***Authors:*** *The authors have addressed the comments for Materials and methods as outlined previously, further details are provided in the Appendix.*

Treatment effects are meagre but that is not a problem, however, the Results should be better described/discussed: highlighting differences in comparison with the “fair” reference (NPK-ref.) and not so much the Control (without any fertilization), but also acknowledging that similarities are also good news if a substitution of the traditional fertilization is envisioned.

***Authors:*** *The authors have addressed this major comment as outlined previously, further details are provided in the Appendix. Briefly, differences are now more clearly highlighted as relative to control or NPK-Ref, and mention of non-significant difference with NPK-Ref is also made (in the context of substitution with seaweed extracts).*

It has a good Discussion section but should be improved, specially the “Future research” part (see more in Specific comments).

***Authors:*** *The authors have addressed the major comments on the Discussion as outlined previously, further details are provided in the Appendix.*

For all this I think that in the present form the manuscript is not yet adequate for publication but I’m sure that after the corrections/adaptations/improvements suggested it would be a very good, interesting and honest work, that would contribute with knowledge and new insights to the field.

***Authors:*** *The authors agree that the manuscript had significant room for improvement and have taken care to consider each specific comment individually and in context, to improve the quality of the reporting as well as the cohesiveness and flow of the manuscript. That said, given the extensive nature of the corrections, the authors addressed comments in the response letter as briefly as possible, except in cases where comments resulted in extensive changes. For ease of reference, the authors first present a summary of the key concerns/comments in the manuscript from both referees and the editor (with line references based on the new MS). This is followed by each comment addressed specifically in the Appendix, grouped by reviewer.*

## Title (full and short versions) ok

Keywords: of the 6 words/expressions included, 2 (seaweed extracts and Vitis vinifera) are already present in the title so they could be replaced by other relevant key words/expressions (like new emerging/expanding vineyard sites). Also, the authors didn’t assess “Photosystems' efficiency”, only of PSII, so I would suggest to replace by “Maximum quantum yield of PSII”.

- The authors take note and suggest as replacements “emergent viticultural regions” and “maximum quantum yield of PSII”.

## Abstract

Line 26 – total “leaf area” of the canopy or of the area of a leaf? – clarify.

- Changed to “individual leaf area”.
- In 2^nd^ mention in materials and methods added statement (hereafter referred to as ‘leaf area’) for the sake of brevity.

Line 26 – “chlorophyll content”– include units.

- Added ‘Index’ as CCI is a unitless index, proportional to chlorophyll content (in mg/cm^2^).

Lines 27-28 – regarding “PSII quantum yield”: is it “effective” or “maximum” quantum yield? Those are very different things – complete and use the same designation throughout the manuscript (ms) for consistency.

- Maximum quantum yield was reported in this instance. However, following the advice from comments in the results and discussion regarding Genty et al. (1989), the authors include both maximum and effective quantum yield as separate parameters. Both are referred to accordingly in the MS.

Line 28 – regarding “These improvements lead xxx”: how do the authors know that this is a cause-effect relationship or the only/main cause of the variation in berry size, mass, etc.? Avoid speculations – rephrase.

- The authors agree with the reviewer, it cannot be solely attributed to treatment as there are multiple factors interacting (season and climatic conditions x treatment). Possible links and alternative cause-effect relationships are explored in the Discussion section.

Lines 32-34 – be specific about the parameters; avoid generalizations that may incur in incorrections.

- Specific parameters are mentioned instead of general categories.
- The authors explicitly state which parameters were improved by *A. nodosum* and *E. maxima*

Line 34 – Clarify what do you mean by “effect sizes”.

- The authors initially intended to calculate and report standardised Cohen’s *d* effect sizes, however this additional made the MS more difficult to follow.
- The authors now mention “the magnitude of the effects”

## Introduction

Line 79 – “have shown promise in grapevines and other xx” – it would be important to indicate some variables/ “numbers” to support this and illustrate que impact of seaweed extracts that is claimed.

- The authors agree that this will make a more impactful statement and have added as an example two variables commonly explored in biostimulant studies done in vineyards. The authors have refrained from adding numerical data as for these studies it typically involves comparing the response per treatment and timepoint as well. The authors believe these are more valuable in the Discussion section of the MS.

Lines 81-83 – regarding this difference in the number of studies between warmer/drought sites vs cooler/more humid sites, and to add pertinence to the approach/idea, the authors should indicate what may be the main limitations in these latter regions and in what way seaweed extracts may help to overcome.

- The authors have expanded on the challenges that these regions face; firstly extreme weather events may also impact these areas, with increasing frequency as mentioned due to climate change, to which these varieties and regions are less adapted to than warmer regions. Additionally they are more likely to face negative consequences such as delayed ripening and increased disease incidence under cooler/more humid conditions

Line 87 – the hyphen is not needed here because you don’t have another last word with one.

- Hyphens removed

Line 94 – regarding “several winegrape cultivars” – the type of climate/regions where the studies were made and mode of applications should be referred.

- The authors refer to this in detail in the discussion; in the interest of brevity these details were omitted from the introduction initially. However, as a demonstrative example, the authors added the details (climate, application regime and cultivar) of an Australian study (Arioli et al, 2021) [6], but refrained from doing so for the cited review article by Gutiérrez-Gamboa and Moreno-Simunovic (2021) [10], as many of these studies are cited in the discussion section of the MS with the relevant details.

L94-98 – Regarding the use of E. maxima – how do the authors picture the harvest and potential wide use of this algae as, currently, the populations in South Africa are at higher risk precisely due do climate changes in water temperature.

- The authors agree that this is an important point of consideration in the climate change related context of the study. To this end the authors have found that in contrast to several species, *Ecklonia maxima* has shown a range expansion in recent years of 70 km eastwards in South Africa (Bolton et al; 2012, Straub et al, 2016) [8,9], facilitated by a local upwelling of cool, nutrient rich water. *E. maxima* also grows over a wide range of temperatures (Bolton, 1985) [7] and is classified as a warm-temperate organism. This is now mentioned briefly in the MS.

Lines 99-100 – how can the authors evaluate this if no parallel/similar study was done in a warmer site (maintaining as much as possible all the other non-climatic factors) and the literature is so scarce with respect to studies in cooler sites? – rephrase.

- Our study was conducted in a cool climate region over two growing seasons (2021 and 2022) to evaluate the growth-stimulating effects of seaweed extracts and explore how these effects may vary across differing seasonal climatic conditions. While our findings are limited to this cool climate context, they contribute to the scarce body of literature on the use of seaweed extracts in such regions .

Line 103 – correct typo (cut the hyphen). Also, what does “grape ripening dynamics” mean exactly - specify.

- Hyphen removed; expanded sentence: (…) grape ripening dynamics in terms of total soluble solids and acid evolution (…)

## Materials and Methods

**Experimental design**

Line 124 – correct typo (without hyphen).

- Corrected to macronutrients

**Vine vegetative characteristics**

Lines 172-174 – indicate the time interval since the authors will assess starch content.

- Specified weekly for shoot length measurement

Line 180 – “Specific leaf area” appears in S2 Table but the procedure to calculate this parameter was not referred in this section – include.

- Added calculation for specific leaf area (SLA)

Line 181 – Biological replicates? n=12 or n=4/combined by block? – specify here.

- Specified: Each dried leaf per replicate vine (n = 12) (…)

Line 188 – use a comma to separate tens or hundreds of thousands, like in 12,500 – apply throughout the ms.

- Could not find PLOS specific reference.

Line 196 – don’t use a point for the operation multiplication in combined units when using the scientific system, use just a space like: 4.5 m s^-1^ or 3500 µmol m^-2^ s^-1^ - apply throughout the ms.

- Replaced all bullet multiplication operators with non-breaking spaces throughout MS.

**Chlorophyll indices and stomatal conductance**

Line 214 – indicate how CCI is calculated and what does this index translates (correlates with total chlorophyll content per area? per mass?) – it is important to interpret the results.

- Specified: “The CCI is a unitless transmittance-based measure of the relative concentration of chlorophyll per unit leaf area”

Line 218 – the same applies to Fv/Fm.

- Clarified: “the parameter F_v_/F_m_ (the ratio of variable fluorescence over the maximum fluorescence), was measured using a handheld Pocket PEA chlorophyll fluorimeter (Hansatech Instruments, Norfolk, United Kingdom). F_v_/F_m_ is frequently used to indicate the maximum quantum yield of photosystem II (a measure of plant performance and stress)”

Line 222 – regarding “to reach the maximum Fv/Fm value” – what does this mean? Fv/Fm is already a parameter that gives the maximum quantum efficiency, supposedly the one and only.

- Clarified: maximum *possible* F_v_/F_m_, samples need to be dark-adapted otherwise the measured F_v_/F_m_ is underestimated.

**Berry classical parameters and morphology**

Line 231 – reformulate the phrase to “Berries were sampled from veráison (XX) until harvest (XX) every xxxx).

- Done as suggested

Line 239 – “berries were crushed”- how, exactly? specify.

- Clarified: “Briefly, the remaining berries were gently crushed by hand, externally through the bag, to extract a representative juice sample for each subblock (n = 4)”

Line 253 – “The berry volume was estimated from length and diameter” - how, exactly? specify.

- Specified: “The berry volume was calculated from the berry length and diameter, assuming a perfect spherical shape”
- Additionally added calculation for berry density to ensure completeness.

**Statistical analysis**

The authors would like to kindly draw attention to the fact that the statistical analysis has been rewritten for clarity and completeness, which required some analyses to be updated to reflect the additional checks for model and method assumptions. The overarching themes regarding the results still hold, however some borderline *P*-values may have changed due to their sensitivity. More robust *P*-values (either side of the cut-off) remained unchanged or shifted marginally.

**A proof of concept is provided on p. 30**

Line 278 – the abbreviation “ANOVA” should be here and used in line 285.

- Moved acronym ANOVA to first mention of ‘analysis if variance’, replaced later mention with ANOVA.

Line 282 – replace “equality” for “homogeneity”.

- Changed to homogeneity.

## Results

**Weather data**

Line 302 – “from April to June” or “in April and June”?

- Corrected to in April and June

**Seaweed extract effect on grapevine vegetative growth and physiology**

Line 315 – replace “At that time” by “On that season”.

- Changed to “During this season”

Line 319 – “longer shoot lengths” – reformulate.

- Dropped ‘lengths’, changed to longer shoots

Line 324 – remove the point in (S2 Table.), it is not needed and the authors didn’t use it in, e.g., (Table1) - apply throughout the ms.

- Removed point from “Table” and “Fig”, seemed to be a naming issue with supplementary tables/figures.

Lines 333-336 – on line 333, referred to “leaf area”, it should be (Fig 2C and D) and on line 336, referred to leaf dry mass, should be (Fig 2A and B).

- Updated reference to figures

Line 355 – It’s “maximum quantum yield of photosystem II” – avoid inconsistencies, the authors use different designations for the parameter Fv/Fm across the ms – revise.

- Corrected to maximum quantum yield in each instance in the MS, otherwise authors simply refer to ‘F_v_/F_m_’
- Additionally the authors hope that with the inclusion of the effective quantum yield ФPSII as an additional parameter of study, that the difference is made pertinent in the MS.

Lines 359-360 - “Treatment” was not associated with differences in stomatal conductance as can be seen in S2 Table and Table 1, only “Time” and “Season” and their interaction gave significant effects (S2 Table) – revise.

- Fixed typo and emphasised that treatment was not associated with differences in stomatal conductance.

Lines 376-383 – To better follow this description authors should highlight these particular time-points on S4 Table. Also, they should include a column with the days 2021(before that of the developmental stages) to better compare with Figure 3.

- The authors fully agree with this point and have implemented the following:
  - Time column in all Supplementary Tables now contain DAA in addition to E-L stage to improve interpretability
  - Timepoints highlighted in S4 Table
- High VPD stress periods shown as light red shaded blocks in Fig 3 to better link climatic conditions to observed changes in fluorescence parameters

Regarding Figure 3 – CCI are very different between years and should be addressed, but specially the decay in Fv/Fm in 2021, the least harsh year, should also be addressed.

- The authors agree that on the first notice these results may seem at odds and therefore included two additional parameters (based on the obtained fluorescence transients) in Figure 3 to provide more support for the observations of reduced CCI in 2022 and lower (but more stable) Fv/Fm values in 2021:
  - The active reaction centres per measured cross section of leaf (RC/CS)
  - The effective quantum yield of PSII (also highlighted by the reviewer in the discussion section)
- The reduced chlorophyll content index is likely ascribed to a downregulation of PSII reaction centres in response to the abundant light and warm climate conditions experienced in 2022. The reduced RC/CS of at the start of the 2021 season (immediately following a warm period) relative to the remainder of the season (which was cooler with high humidity) provides further support. The authors included a reference to Keller (2005) and Chen & Cheng (2003).
- Regarding the decay in Fv/Fm, this is likely ascribed to the moderate temperatures in combination with high humidity which led to increased downy mildew infections across the vineyard.

**Seaweed extract effect on reproductive parameters**

Line 390 – About “reproductive” – authors should reconsider designation, maybe fruit? berry? as reproductive also implies date of flowering, flowers, etc.

- Updated to berry development or berry quality throughout the manuscript

Lines 391-392 – On S5 Table, “strong main effects” can only be seen on those 4 berry parameters with “Time”; “Season” as “Treatment” only had significant effects on “berry volume” and “berry mass” – reformulate.

- Updated text to correctly reflect results from the analysis in S5 Table:
  - Strong main effects of Season and Treatment on berry volume, mass, density and DM%
  - Strong effects of season x time interaction and VPD on berry density and DM%
  - No association with treatment, but low values for volume and mass which prompted additional per season analysis

Line 401 – regarding “averaged over the ripening period” – more importantly than the average are the berry “metrics” at harvest, better related with fruit yield and quality, don’t you think? By Figure 4 there are no differences on those two variables in the last timepoint (and for some reason you depict the last timepoint here and not in Figure 2) – defend your ideas and explain better.

We appreciate the reviewer’s comment and agree that the berry metrics at harvest are critical indicators of yield and quality. However, our approach of averaging berry parameters over the ripening period was chosen because the dynamics of berry maturation provide essential insights into fruit development that the harvest timepoint alone does not capture. Specifically:

1. **Rate of Maturation Matters:**

While the final harvest metrics are undeniably important, the rate at which berries mature affects not only their biochemical composition (sugar accumulation, acid degradation, flavour compound development) but also the timing of harvest, which in turn can influence both yield and quality. Analysing these parameters over the period provides a more integrated view of the maturation process and highlights subtle treatment effects that may be diluted or masked when looking solely at the endpoint.

2. **Link to Vegetative Function and Photosynthesis:**

Our study emphasizes the role of a properly functioning photosystem in driving overall vegetative growth. Since photosynthetic efficiency throughout the season directly impacts resource allocation, carbohydrate production, and ultimately the ripening process, we believe that the cumulative effect (captured by the averaged values) is highly relevant. These averaged metrics can reveal how treatments improve or impair the efficiency of berry development over time—even if, by harvest, differences have converged.

We intentionally depict the final timepoint for berry metrics (in Figure 4 and 5) to provide a direct link to yield and quality outcomes, while Figures such as Figure 2 and 3 (which focus on leaf and photosynthetic parameters) reflect the overall physiological status leading up to the ripening period. This dual approach allows us to connect both the integrated physiological responses and the endpoint fruit metrics.

Lines 405-407 – shorten the phrase as: “xxx treated with A. nodosum and NPK-ref were on average xxxx” – apply to similar situations in order to condense the ms, ease the reading, and to better retain the essentials.

- Corrected as suggested for all instances in the MS

Lines 399-408 – regarding comparisons with the Control (and this also applies to similar situations) – the only “fair” comparisons are those of the treatments (extracts) with the NPK-ref and that should be addressed without fear, there is a lot of room to improve this approach leaving that for Discussion or Future pespectives. Clearly, for most of the parameters there are no significant differences comparing to the NPK-ref, but there are some and those should be highlighted.

- We thank the reviewer for this valuable feedback. We agree that the most meaningful comparisons in our study are those between the treatments (extracts) and the NPK-reference. In the revised manuscript, we have adjusted our reporting to place greater emphasis on these comparisons. Specifically, when a difference with the water control is observed, we also contextualise the result in terms of pairwise comparison to the NPK-Ref treatment. Although many parameters did not show significant differences compared to the NPK-ref, the few instances where significant improvements were observed have been highlighted. We have also expanded our Discussion section to address how our approach could be further refined in future studies. We believe this revision better aligns with agronomic practices and clarifies the implications of our results for yield and quality.

Line 439 – indicate Figure or Table in the end of this statement.

- Corrected as suggested

**Seaweed extract effect on harvest parameters**

Line 466 – correct typo (cut the point)

- Corrected as suggested

## Discussion

**Seaweed extracts show environment dependent improvements in leaf anatomy and vine physiological parameters**

Lines 490-491 – a hyphen is needed in “environment-dependent”.

- Corrected as suggested

Line 492 – Better start with “As for other plants, xx” – foliar photosynthesis is crucial for all the plants, even if other organs could be photosynthetic, like aerial roots, petals, sepals, fruits, etc.

- Corrected as suggested

Lines 493-494 – If “specific leaf area” is an important parameter it should be better “treated” in M&M and Results. As I can recall it only appears once in Results and no effects were registered in SLA – avoid referring to results/parameters/factors of less or no importance to emphasize the most relevant parts of your work.

- The authors agree with the reviewer, the SLA calculation has been better described in methods, and we mentioned a lack of observed differences in the results section, and removed it from the discussion.

Lines 494-496 – this is an example where the authors should emphasize the differences found with A. nodosum comparing to NPK-ref to highlight the relevance of the extract if a substitution in the type of fertilization is envisaged (apply to other analogous situations).

- Differences were with control only, but in 2022 extracts performed comparably to NPK-Ref, suggesting possible substitution.

Lines 499 – Take note that Fv/Fm is not the best parameter to assess plant stress

(it’s much less susceptible than other parameters as the effective quantum yield (F_PSII_), for instance), and it doesn’t correlate to “photosynthetic capacity” as F_PSII_ (see, e.g, Genty et al. 1989 - BBA, 900, 87-92), so it would be better to replace by “Photosynthetic potential” or “Photosynthetic performance” – this can be used here to explain the poor impact on Fv/Fm by the treatments tested. Also, a precise information of what CCI indicates should be on M&M and Results, and recall it here to better understand the impact of the increase in CCI with A. nodosum.

- The authors thank the reviewer for sharing their insight, after reading Genty et al (1989) and a thorough review of the work done by Strasser et al. on analysing the fluorescence transient, the authors have included two additional datasets;
  - Ф_PSII_ the effective quantum yield as suggested, to be interpreted in combination with Figure 3E,F for the maximum quantum yield (F_v_/F_m_).
  - RC/CS – the number of active (Q_A_-reducing) reaction centra per cross section which should substantiate the observations made for the chlorophyll content index (CCI).
  - Furthermore the authors clarified in the Methods section that the CCI is a transmittance‑based measure of the relative concentration of chlorophyll per unit leaf area, with a power-law relationship with respect to absolute chlorophyll concentration (Parry et al., 2014). A brief mention is made in the results and discussion sections to enhance readability as suggested by the reviewer.

Lines 502-503 – Figure 2 says otherwise, there was more vegetative growth on 2021… - reformulate

- The authors note that Figure 2 refers to leaf area and mass, both which were higher in 2021, however the statement refers to fluorescence parameters and CCI per unit measured leaf area, where the following was observed:
  - Reduced CCI from 2021 to 2022 in addition to a trend of decreasing CCI values as the season progresses. The significant reduction in CCI from 2021 to 2022 is likely a response to regulate against potential photosystem damage resulting from high temperatures and light.

Line 508 - correct typo (separate the units)

- Corrected as suggested

Line 511 – Better to use RuBisCO

- Corrected as suggested

Line 514 – Better to use “drier”, the authors don’t have a measure of water stress or even of water status either of soil or grapevines by the measurement of pre-dawn and/or midday water potentials. In this sense, maybe it should be added that, probably, not even water stress conditions were installed, supporting the no effect on stomatal conductance (together with the anisohydric characteristic of the cultivar).

- Added details on lack of water stress due to high water table from previous season’s rain, and clarified that proposed effect of anisohydric characteristic was not explicitly confirmed as leaf water potentials were not measured.

Line 521 – Definitely one thing to “optimize” is the type and regime of application of the extracts (frequency, concentration, foliar vs by irrigation), as well as to monitor the possible lixiviation of the extracts from the leaves by the rain – the authors should address these aspects because it may explain the lack of differences comparing to NPK-ref.

- The authors take note of the very relevant aspects regarding treatment application regime and concentration, that can be valuable future investigations to optimise the treatment outcomes. For this study, the authors chose to focus on different species at supplier recommended concentrations. Additionally lixiviation/leaching was not considered a problem in this study as there was no rain for 24-36 hours post-application during the first 4 out of 5 time points in 2021, while no rainfall post-application was recorded for all days in 2022. Furthermore, the authors added details on the possible lack of water stress due to the high water table, from the previous season’s (2021) rainfall.

Lines 524-525 – add that this stands for the seasonal average and not just to the last time-point (apply to analogous situations).

- Emphasised effect was averaged over season where relevant and clearly highlighted non-significance of treatment at harvest.

Lines 527-529 – There was a smaller leaf area and leaf mass in 2022 (see Fig. 2) – rephrase and avoid speculation.

- Emphasised that the specific treatment, *A. nodosum* had a larger leaf area compared to control in 2022, and further highlighted that globally, leaves were smaller in 2022 compared to 2021.

Lines 534-536 – Globally, it is also be important to address the NPK-ref nutrient content because this (supposedly) reference condition lack all the other macro and micronutrients that the extracts have, specially A. nodosum, that is much richer than E. maxima on most of it (S1 Table), what may explain the poor differences observed between A. nodosum and the NPK-ref and the poorest performance with E. maxima.

- Corrected as suggested

Line 539 – “higher” than what? All the other treatments? – indicate and refer to a Fig/Table.

- Section rewritten with references to relevant Figures.

**Yield indicators may be improved by seaweed extracts applications**

Line 558 – it’s not “from 2022 to 2021”, is it? – correct.

- Corrected as suggested

Lines 564-568 – Regarding this discussion it is important to remember that plant/algal extracts have secondary metabolites that may confer protection against water-, light-, temperature-induced stresses, and that could explain the higher impact in warmer/driers conditions – I suggest to add that to the discussion.

- The authors take note and appreciate the emphasis on secondary metabolites. While these were not quantified in this study, the authors have added possible mechanistic explanations for the observed differential effects based on literature, under the assumption that similar levels of secondary metabolites were present in these particular batches. For future studies the authors would recommend a full metabolite analysis and hormone content assays in addition to the nutrient content analysis that was performed,

Lines 569-570 – Before the “Additionally, although outside of the scope”, future research” should focus in many other aspects, as those already referred regarding extract application, but also chemical composition of the extracts in terms of secondary metabolites, and monitor plant water status.

- The authors take note that the study limitations were not clearly highlighted in the discussion. As corrective measure, the authors did the following:
  - Mention specific limitations alongside their relevant observation.
  - Summarised key study limitations to consider when conducting future studies.

## Conclusions

Lines 577-579 – again, be clearer/more truthful and objective regarding the effect of A. nodosum comparing to NPK-ref, remember that it doesn’t need to be better but if it’s as good as the traditional practice NPK is already good, don’t avoid that “fight” because you don’t need to.

- The authors take note that many of the comparisons were only made in relation to differences between treatments (mainly from control), while not explicitly stating similarities with, for example, the NPK-Ref treatment. The authors hope that the rewritten portions of the results and discussion sections are more holistic in including non-significant differences from treatments with the NPK-Ref treatment, as a possible substitution for synthetic foliar fertilization.

## References

Didn’t revise systematically but it seems ok.

- The authors would like to bring attention to the fact that referencing software was switched from Mendeley to Zotero. References were compared afterwards to ensure no stylistic changes occurred during the transfer.

## Figures/Tables

S1 Table – species in italic (footnote); the reference to the service responsible for the chemical analysis of commercial formulations should be included.

- Species names are now italicized, reference to Bodemkudige Dienst van België following reference method of analysis was added

S2 Table – revise on footnotes “Asterisks indicate significant differences between the treatments (0.05 > P > 0.01: * ; 0.01 > P > 0.001: ** ; P < 0.001: ***).” - it should be: (0.01 < P ≤ 0.05: * ; 0.001 < P ≤ 0.01: ** ; P ≤ 0.001: ***).

- The authors thank the reviewer for this correction and have implemented it as suggested across all figures and supplementary figures in the MS

S2 Table - “Stomatal conductance (σ)” is not a fluorescence index – revise table format.

- The authors take note and have changed the subsection heading to “Gas exchange and fluorescence indices”

Figure 2 – I think the units of “Leaf dry mass” are mg (compare with that of Table 1) – correct.

- Corrected leaf dry mass units for both Figure 2 and Table S2 to mg

Table 1 – remove the number 1 after “Comparison”, I think there’s no need, it goes with the analysis/table contents just leave the footnote (the same applies to similar situations as in Table 2, S2 and S5 Table). Also, as the variables CCI and Fv/Fm were already specified, the numbers 2 and 3 and respective footnotes with their full names are also unnecessary. On the other hand, the numbers inside the table refer to what? Average values over time? Last time-point? Compare with footnotes in Table 2, an analogous table and better explained – clarify/ harmonize.

- The authors have carefully considered these aspects and incorporated them into the relevant tables and supplementary tables.

Figure 3 – Change “PSII efficiency (Fv/Fm)” to “PSII maximum quantum efficiency”, “PSII maximum quantum yield” or just “Fv/Fm”.

- The authors have completely revised Figure 3 as mentioned above in the results section.

# Reviewer 2:

Dear Authors,

I have carefully reviewed your manuscript, which addresses a highly relevant and timely topic with practical implications for viticulture in the face of current climatic crisis. Overall, the manuscript is well-written and logically structured. However, several aspects, particularly related to the Results and Discussion sections, require further attention and improvement. A detailed PDF highlighting specific issues is attached to this report. Still, below, I am outlining the major points for your consideration:

***Authors:*** *The authors would like to thank the reviewer for their kind words regarding the overall writing and structure of the manuscript. The authors further agree that the results could benefit from being more clearly structured, and the discussion by having more depth and exploring possible modes of action for the observed treatment effects. We appreciate the reviewer’s suggestions and have incorporated them in as much as possible. General comments are addressed first, followed by minor and detailed comments per section.*

## General comments

1. Why did the frequency of application differ between years? This aspect is important, especially because you often compare the efficiency of the treatments between 2021 and 2022 growing seasons.

***Authors:*** *Treatments were applied with the aim of targeting specific phenological development stages based on supplier recommendations. Differences in application dates were the result of slower phenological progression in 2021 compared to 2022 due to cooler and wetter climatic conditions during the targeted application period (±2 week pre-flowering to start of véraison). This was made more clear in the Materials and Methods and we refer to results of the weather data collected during the season.*

2. In the M&M, you refer that the NPK treatment was performed in order to adjust the nutrient values of the extracts; however, in the discussion, you clearly mention the big differences, regarding nutrient levels, between the two seaweed extracts. So, how was this performed?

***Authors:*** *The authors agree that the explanation of the NPK-Ref treatment preparation could be better explained. To clarify, the NPK-reference was prepared to mimic only on the measured N, P and K values of the seaweed extract treatments, as closely as possible. When extracts differed significantly in one of the components, the highest value was used in the NPK formulation. While N and K were closely approximated, the P-content was 67% higher than the highest P-containing seaweed (*E. maxima*). The Materials and Methods section has been updated to ensure this distinction is explicitly clear, as shown in S1 Table as well.*

3. The manuscript lacks clarity regarding the methodology for post-hoc tests in cases where significant interaction between factors was detected. From the presentation of the figures, it appears that post-hoc tests were conducted uniformly, regardless of the ANOVA results. I am asking this because, as you known, in cases of significant interaction among factors, the simple main effects cannot be easily interpreted, since the response to one factor depends on the other. There are already some programs that can adjust the p-values to allow a fair comparison.

***Authors:*** *The authors take note and agree that main effects are difficult to interpret when interactions are significant. Fortunately, for the season-specific models, most parameters did not have a significant interaction and so main effects could be isolated. Regardless, to ensure statistical robustness against type-I errors, while avoiding the inflation of type-II errors, the authors conducted post-hoc analyses using a-priori contrasts. Specifically, for berry quality parameters this involved testing 6 pairwise differences between treatments over the ripening period, in addition to sequential contrasts between timepoints (3-4) and finally 6 pairwise differences between treatments at harvest. This resulted in a total of 15-16 comparisons, which were corrected for multiple comparisons using Šidák’s adjustment. This has been outlined in the materials and methods section.*

4. I encourage you to avoid overemphasizing non-significant results in the description of the obtained data. Additionally, please consider reorganizing the figures in line with the suggestions provided in the attached PDF.

***Authors:*** *The authors have removed all non-significant results, specifically pertaining to previously mentioned % differences from the water-only control that were non-significant. However, in accordance with reviewer 1, non-significance (without % difference) is reported explicitly when it concerns seaweed comparisons to NPK-Ref treatments.*

5. The Discussion section would benefit from a major revision, especially addressing the mechanisms and biological effects underlying the observed treatment responses. Currently, the justification frequently centers on differences in nutrient inputs, but seaweed extracts also contain a diverse range of biologically active compounds that can significantly influence plant responses. I recommend incorporating this aspect into your discussion to provide a more comprehensive analysis.

***Authors:*** *We agree with the reviewer and have therefore added possible mechanisms of action based on, for example, phytohormone content or osmolytes such as glycine-betaine. However as these compounds were not quantified in this study, the authors made clear mention of this in relation to literature, to differentiate from the nutrients that were quantified, and to avoid speculating too much. While the discussion brings more depth, there is still room for future studies to test the effects of these compounds individually and in combination(s) to further elucidate the effects of seaweed extracts.*

## Abstract

L20 biostimulant potential of some species, such as Ecklonia maxima. Here, it would be appropriate to refer which kind of algae is this species \(red, brown, green?\)

- Corrected as suggested

L22 Commercial formulations? Or did the authors prepare the extracts? Aqueous or organic extracts?

- The authors clarified that two commercial extracts were used, based on two species.

L25 In terms of growing season, when were the treatments applied? Throughout the whole life-cycle? only in the vegetative growth period? Covering both vegetative and reproductive phases?

- The authors specified that vegetative and reproductive growth (from flowering to ripening) were covered.

L25-28 In relation to the CTL \(no treatments\) or to the application of NPK?

- Clarified in relation to control (no treatment). Additional mention was made when there was no difference compared to NPK-Ref, as has been done throughout the MS based on the recommendation by reviewer 1.

L29 At this point, it is clear that the application was made along the whole life-cycle, which is good. Try to introduce this info above.

- Corrected as suggested

## Introduction

L39 Separate into a different sentence.

- Corrected as suggested

L42 Currently, most \(...\)

- Corrected as suggested

L43-45: I would not say "it is believed", since we are already dealing with the damage caused by climate change

- The authors agree and have strengthened the statement regarding climate change effects.

L48-49: “Results vs resulting”

- Changed to “leading to”L63: (...) crop quality, while reducing the need for synthetic agrochemical inputs
- Corrected as suggested

L69: “more clear”

- Changed to “clearer and more concise (…)

L75 promising features

- Corrected as suggested

L79 stress-ameliorative features

- Corrected as suggested

L83-85 subjected; Here, it would be appropriate to give some clear examples about this variability.

- The authors agree that doing so will make it more clear that there is inherent variation associated with seaweed extracts, which might affect their performance in agricultural studies. Therefore a reference is made to the range in which the macro nutrients are found, and a reference to a specific study that showed inter-season variation.

L102 with valuable

- Corrected as suggested

L103 Did the authors perfom any kind of climatic monitoring, in terms of temperature and precipitation, for instance?

- Yes, materials and methods of climatic monitoring are expanded following the Vineyard subsection.

## Layout and Experimental design subsections.

L111 were

- Corrected as suggested

L120 These treatments were comercial?

- Yes, except the control treatment which was demineralised water.

L123 water + tween, right? please mention this.

- Rephrased to “a blank treatment containing only demineralised water and Tween‑20 with no additional compounds (Control)”.

L124 Can you provide some details about the product?

- To do; add as appendix

L131 First the full name, then the abbreviation.

- Corrected as suggested

L133-136 Why these differences between years?

- The authors refer the reviewer to the major comments for a detailed explanation, in brief; different climatic conditions led to different rates of development. In order to target specific, comparable timepoints and to avoid treatment wash-off by rain, the supplication frequency differs between years.

L294 Cumulative growing degree days

- Corrected as suggested

## Results:

I think the results are generally well-described, however the statistical procedure should be better explained, especially in what concerns the procedure executed when an interaction was found. **If there is an interaction, the main facto\ r effects cannot be analysed separetely. Did the authors have this into account?** Furthermore, I think the figures could be improved. Authors often represent the response across time to draw temporal curves, but this is not correct, in my opinion,\ because you did not measure in a continuous way. Also, in the results, you barely compare the different time-points between them, so, my suggestion is: Only use bar graphics, organized between seasons \(2021 separated from 2022\), with each trea\ tment consisting of 4 bars \(each time-point\). The figures would be very much easier to analyse and I think you can retrive the same kind of information. Lastly, in general, the highest value should be the 'a' - this is not happening here. pleas\ e revise this.

- The authors agree that previously, the statistical pipeline was not well described and have therefore done an extensive revision to include additional diagnostic checks to ensure the most parsimonious and accurate models are used in the analysis. The procedure was outlined in brief under; **Major comments** – “Statistical Analysis Overview”.
- **A proof of concept is provided on p. 30**
- The authors agree that strictly speaking, measurements were not continuous, however the time series plots have the added value of showing trends over a season, which the authors have found from previous experience to be lacking with grouped bar plots. Furthermore, using time series plots in vineyard field trials are commonly done, as shown by [2,11–14].
- Below the authors show an illustrative comparison of time series with violin-boxplot inlay (left) vs grouped bar plots (right):

- The authors believe the addition of the inlay violin-boxplots improve the interpretability of the results greatly and allow clear differences within the main effect of treatment to be seen.
- The updated plots include the following additional elements:
  - Inlay violin-boxplot or boxplot for berry quality parameters for entire season data
  - Markers indicating biostimulant application dates
  - Light blue shaded areas indicating high RH% and clouds, and light red shaded areas indicating elevated VPD (>3 kPa).
  - Model legend showing which factors and interactions are significant.

L331 This was not significant - it is "ab" so it means it is identical to the control.

- The authors recognise the importance of not overstating results that did not show significance at the level of α = 0.05, however, it cannot be concluded that these treatments are “identical” to the control. Rather it should be said that no significant difference was found, which could be due to various reasons, ranging from the treatments indeed being very similar, or more likely that the study did not have sufficient statistical power to detect differences – the authors make a reference to this in the limitations of the study design in the Discussion section.

L332-333 If it is not significant, please don't refer to % of change.

L333-337 Same comment as above. Please stick to only significant differences.

- The authors have removed mention of % difference in instances where non-significant effects were reported.

L338 How were the ANOVAs done? three-way? If so, because there are 3 factors, then it is advisable to show the differences for each factor alone and their interactions.

- The authors thank the reviewer for this recommendation and refer the reviewer to the updated description of the statistical analysis method. In brief ANOVA was done on LMM’s with a varying number of factors that define the most optimal model describing the data (determined with the corrected Aikaike Information Criterion AICc), to determine which factors are significant. Depending on these results, further 2-way ANOVA’s were conducted to better elucidate treatment effects, in the majority of instances the interaction effects were not significant between treatment and timepoint for these within season analyses.

L366-368 In the figure, there is an * in the last time point of 2022 campaign.

- The authors decided to do a thorough check of all the datasets of the study, and in this specific instance a formula error led to the incorrect assigning of CCI data to treatment on the final date. The formula was corrected and there is no longer a local, significant difference; instead the data follow the same pattern throughout the season.

L369-371 For all time points? it is not clear.

- The increase was on average (across timepoints) over the season.
- The authors have clarified throughout the MS when the time-averaged value is reported.
- In particular for berry quality and ripening parameters, a distinction is made between the time-averaged value and harvest value. Significant differences (or their absence) at harvest are directly reported afterward.

L384 (Fig 3) Why isn't this figure organized as the previous one to ilustrate the main effects of the treatments in CCI and Fv/Fm?

- After careful consideration the authors have decided to include a combination plot with a timeseries over the season and an inlay of the average treatment effect as a violin-boxplot. The authors refer the reviewer to individual figure captions where a distinction is made whether the inlay plot is the season average (for vegetative and physiological parameters), or the harvest timepoint (for berry quality parameters).

L400-402: This refers to the * placed in the Figure B between 80-100 days, right? It is confusing, because in the Figure the graphic illustrating the effects of the treatment does not have significant differences, since it represents the harvest \ time-point.

- The authors have decided to remove asterisks indicating specific significant timepoint differences from all time-series figures, instead asterisks are used in a plot legend, to indicate whether Treatment, Time or Treat x Time has a significant influence on the plotted parameter. Furthermore, uppercase alphabet letters show differences between treatments, averaged over the season, and are located at the bottom of the inlay violin-boxplot. For berry quality/ripening parameters, the additional lowercase letters at the top relate to differences between harvest timepoints.

L407-408: Why are the data from the last timepoint plotted in the figure and not those for which significant differences were found?

- Typically vintners are interested in the quality of the berries at harvest, even though differences at timepoints before harvest would point toward a different rate of ripening, which influences final wine quality as well. This is emphasised more clearly in the text, while retaining the values at harvest.

## Discussion

This section should be improved. The authors discuss their obtained data in a very shallow way, not focusing on the mechanisms and biological effects underlying the observed responses. What are the differences between extracts, besides \ nutrient profiling, that might explain these differences? Also, comparing to the inorganic fertilizer, what are the real gains of using these kind of approaches? This aspects should be discussed to further support the importance of the study.

- The authors take note and agree that the possible mechanisms were not properly discussed, in addition to the chemical differences between the extracts. The rationale was to avoid as much as possible speculation in the first instance, and also to focus on the observations of the current study in the context of the study design and environmental conditions.
- That said, the authors agree that there is great value in expanding the discussion by integrating a larger variety of literature that focusses on extract composition and modes of actions, in relation to these compositional differences (while keeping in mind the differing climatic conditions between seasons within this study). The authors also clearly state which conclusions are drawn directly from the data and which are more exploratory based on literature.
- For future studies the authors would recommend a full metabolite analysis and hormone content assays in addition to the nutrient content analysis that was performed.
- The authors note that in many instances the extracts performed comparatively well compared to the NPK-Ref treatment, and more clearly state that seaweed extracts could be used as an alternative/adjunct to NPK foliar sprays, as opposed to being an improvement on NPK from a plant growth promoting point of view.

L502 Is this biologically relevant?

- In the context of controlled plant stress experiments it’s not relevant, where stressed vines typically have a 30-50% reduction in Fv/Fm. However in our study it did represent a sustained small-medium effect size using Cohen’s *d* standardized effect size (small = 0.2, medium = 0.5, large = 0.8) over the season with Cohen’s *d* = 0.42. Compare this to the medium to large effect size of the CCI for 2021 with Cohen’s *d* = 0.65.

L502-505 Why is there a reduction in chlorophyll content and an increase in photochemistry processes, which, in the first place, depend on chlorophylls?

- We agree that at first glance the results seem at odds, however although chlorophyll is essential for photochemistry, under high irradiance plants often reduce chlorophyll content—and, in our case, even RC/CS—to prevent overexcitation and photodamage. Despite this reduction, the efficiency per reaction centre improved, as reflected by the higher Fv/Fm and ΦPSII under warmer, sunnier conditions. We interpret this as a protective adjustment, where improved enzymatic activity—such as enhanced RuBisCO activation and more efficient PSII repair—helps sustain higher photochemical efficiency even with lower overall chlorophyll and RC/CS. We hope this clarifies our findings.

L506 resulted

- Corrected as suggested

L549- 551 This sentence is not clear. Can you rephrase it, please?

- The study by Conradie (1981) [15] quantified the amount of K that is taken up by grapes during ripening. The aim was to explain that applying *A. nodosum* at the dosage specified in the Methods, would only contribute 0.3% of this total amount of K that is expected to be taken up by the maturing berries.
- The authors have decided to remove this sentence, and instead mention that “*A. nodosum’s* substantially greater potassium content (~144-fold higher than *E. maxima*; S1 Table), essential for osmotic balance and water transport, could partly explain its superior performance, in addition to the presence of osmolytes like glycine-betaine” (which is based on literature.
- The authors hope to avoid too much speculation on this regard, as osmolyte content in the berries and leaves were not measured in this study.

L558-559 So, based on these data, the application of NPK results in better yield outcomes?

- This cannot be concluded statistically based on within season comparisons, however based on the within treatment contrasts comparing 2021 to 2022 for each treatment, both NPK-Ref (+80%) and *E. maxima* (+60%) had significantly increased yields, while in comparison the observed average increases for *A. nodosum* (+53%) and water control (+28%), were not statistically significant (*P* > 0.05).
- The authors tried not to speculate too much (only two seasons are compared, the study was done in a single biogeographical region). Additional studies need to be done to conclude whether: (1) NPK-Ref outperforms the seaweed extracts on yield parameters; and (2) *A. nodosum* does not significantly improve yield from one season to the next (while controlling for in-field climatic conditions).

L580 Since this was only observed once in one of the experiments, I would be very cautious with this affirmation.

- In agreement with the cautious approach followed in the previous comment, the authors use the phrase “shows potential” instead of promise.
- Furthermore the authors emphasise the need for additional studies to confirm whether this observation is a true effect, or a statistical anomaly.

L581-582 What about the other components of the extract? Seaweeds are rich in phytohormones, sugars, phenolics... they could have \(and probably did\) played a role in these responses.

- The authors have expanded in this discussion and the conclusion that differential responses are likely due differences in chemical composition and hormonal profile, while using Mg, K and S differences (that were measured in the study) as an additional aspect that might contribute to the differential effects.

# References

1.	Salvi L, Brunetti C, Cataldo E, Niccolai A, Centritto M, Ferrini F, et al. Effects of *Ascophyllum nodosum* extract on *Vitis vinifera*: Consequences on plant physiology, grape quality and secondary metabolism. Plant Physiology and Biochemistry. 2019;139: 21–32. doi:10.1016/j.plaphy.2019.03.002

2.	Tombesi S, Frioni T, Sabbatini P, Poni S, Palliotti A. *Ascophyllum nodosum* extract improves leaf thermoregulation by reducing stomatal sensitivity to VPD in *Vitis vinifera* L. Journal of Applied Phycology. 2021;33: 1293–1304. doi:10.1007/S10811-020-02336-5/FIGURES/10

3.	Battacharyya D, Babgohari MZ, Rathor P, Prithiviraj B. Seaweed extracts as biostimulants in horticulture. Scientia Horticulturae. 2015;196: 39–48. doi:10.1016/j.scienta.2015.09.012

4.	Garcia-Vaquero M, Rajauria G, Miranda M, Sweeney T, Lopez-Alonso M, O’Doherty J. Seasonal Variation of the Proximate Composition, Mineral Content, Fatty Acid Profiles and Other Phytochemical Constituents of Selected Brown Macroalgae. Mar Drugs. 2021;19: 204. doi:10.3390/md19040204

5.	Shukla PS, Mantin EG, Adil M, Bajpai S, Critchley AT, Prithiviraj B. *Ascophyllum nodosum*-based Biostimulants: Sustainable Applications in Agriculture for the Stimulation of Plant Growth, Stress Tolerance, and Disease Management. Frontiers in Plant Science. 2019;10. doi:10.3389/fpls.2019.00655

6.	Arioli T, Mattner SW, Hepworth G, McClintock D, McClinock R. Effect of seaweed extract application on wine grape yield in Australia. Journal of Applied Phycology. 2021;33: 1883–1891. doi:10.1007/s10811-021-02423-1

7.	Bolton JJ, Levitt GJ. Light and temperature requirements for growth and reproduction in gametophytes of *Ecklonia maxima* (Alariaceae: Laminariales). Mar Biol. 1985;87: 131–135. doi:10.1007/BF00539420

8.	Bolton JJ, Anderson RJ, Smit AJ, Rothman MD. South African kelp moving eastwards: the discovery of *Ecklonia maxima* (Osbeck) Papenfuss at De Hoop Nature Reserve on the south coast of South Africa. African Journal of Marine Science. 2012 [cited 26 Feb 2025]. Available: https://www.tandfonline.com/doi/abs/10.2989/1814232X.2012.675125

9.	Straub SC, Thomsen MS, Wernberg T. The Dynamic Biogeography of the Anthropocene: The Speed of Recent Range Shifts in Seaweeds. In: Hu Z-M, Fraser C, editors. Seaweed Phylogeography: Adaptation and Evolution of Seaweeds under Environmental Change. Dordrecht: Springer Netherlands; 2016. pp. 63–93. doi:10.1007/978-94-017-7534-2_3

10.	Gutiérrez-Gamboa G, Moreno-Simunovic Y. Seaweeds in viticulture: a review focused on grape quality. Ciência e Técnica Vitivinícola. 2021;36: 9–21. doi:10.1051/ctv/20213601009

11.	Frioni T, Sabbatini P, Tombesi S, Norrie J, Poni S, Gatti M, et al. Effects of a biostimulant derived from the brown seaweed *Ascophyllum nodosum* on ripening dynamics and fruit quality of grapevines. Scientia Horticulturae. 2018;232: 97–106. doi:10.1016/j.scienta.2017.12.054

12.	Frioni T, VanderWeide J, Palliotti A, Tombesi S, Poni S, Sabbatini P. Foliar vs. soil application of *Ascophyllum nodosum* extracts to improve grapevine water stress tolerance. Scientia Horticulturae. 2021;277: 109807–109807. doi:10.1016/j.scienta.2020.109807

13.	Santaniello A, Scartazza A, Gresta F, Loreti E, Biasone A, Di Tommaso D, et al. *Ascophyllum nodosum* seaweed extract alleviates drought stress in Arabidopsis by affecting photosynthetic performance and related gene expression. Frontiers in Plant Science. 2017;8. doi:10.3389/fpls.2017.01362

14.	Palliotti A, Tombesi S, Frioni T, Famiani F, Silvestroni O, Zamboni M, et al. Morpho-structural and physiological response of container-grown Sangiovese and Montepulciano cvv. (*Vitis vinifera*) to re-watering after a pre-veraison limiting water deficit. Functional Plant Biology. 2014;41: 634–634. doi:10.1071/FP13271

15.	Conradie WJ. Seasonal Uptake of Nutrients by Chenin Blanc in Sand Culture: II. Phosphorus, Potassium, Calcium and Magnesium. South African Journal of Enology & Viticulture. 1981;2. doi:10.21548/2-1-2403

## Statistics example: RC/CS Top 5 models

| **Mod num** | **(Int)** | **Block** | **maxT_1** | **Pseudo_EL** | **RH** | **Treat** | **VPD** | **VPD_av** | **Year** | **Pseudo_EL:Trt** | **Treat:Year** | **df** | **logLik** | **AICc** | **delta** | **weight** |
| --- | --- | --- | --- | --- | --- | --- | --- | --- | --- | --- | --- | --- | --- | --- | --- | --- |
| 122 | 4449 | + |  | + |  | + | -226.4 | -30.56 | + | + | + | 51 | -5382.6 | 10874.5 | 0.0 | 0.89 |
| 108 | 4413 | + | -1.143 | + |  | + | -217.6 |  | + | + | + | 51 | -5385.3 | 10879.9 | 5.4 | 0.06 |
| 110 | 4293 | + |  | + | 1.146 | + | -214.4 |  | + | + | + | 51 | -5385.8 | 10881.0 | 6.5 | 0.035 |
| 106 | 4388 | + |  | + |  | + | -221.4 |  | + | + | + | 50 | -5388.1 | 10883.3 | 8.8 | 0.011 |
| 116 | 4191 | + | -26.54 | + |  | + | 346.9 |  | + | + | + | 51 | -5387.9 | 10885.2 | 10.6 | 0.004 |

### **Model comparison (AIC_c_, GVIF_adj_, R^2^)**

| > moda <- get.models(model_set, 1)[[1]] | | | |  | > moda <- get.models(model_set, 2)[[1]] | | | |  | > moda <- get.models(model_set, 3)[[1]] | | | |  | > moda <- get.models(model_set, 4)[[1]] | | | |
| --- | --- | --- | --- | --- | --- | --- | --- | --- | --- | --- | --- | --- | --- | --- | --- | --- | --- | --- |
| > formula(moda) | | | |  | > formula(moda) | | | |  | > formula(moda) |  |  |  |  | > formula(moda) |  |  |  |
| RC_CS ~ Block + VPD + VPDavg + Pseudo_EL + Treat + Year + (1 \| Vine) + Pseudo_EL:Treat + Treat:Year | | | |  | RC_CS ~ Block + maxTemp_1 + VPD + Pseudo_EL + Treat + Year + (1 \| Vine) + Pseudo_EL:Treat + Treat:Year | | | |  | RC_CS ~ Block + RH + VPD + Pseudo_EL + Treat + Year + (1 \| Vine) + Pseudo_EL:Treat + Treat:Year | | | |  | RC_CS ~ Block + VPD + Pseudo_EL + Treat + Year + (1 \| Vine) + Pseudo_EL:Treat + Treat:Year | | | |
| > AICc(moda) |  |  |  |  | > AICc(moda) |  |  |  |  | > AICc(moda) |  |  |  |  | > AICc(moda) |  |  |  |
| **[1] 10874.53** |  |  |  |  | **[1] 10879.94** |  |  |  |  | **[1] 10881** |  |  |  |  | **[1] 10883.34** |  |  |  |
| > vif(moda) |  |  |  |  | > vif(moda) |  |  |  |  | > vif(moda) |  |  |  |  | > vif(moda) |  |  |  |
|  | **GVIF** | **Df** | **GVIF^(1/(2*Df))** |  |  | **GVIF** | **Df** | **GVIF^(1/(2*Df))** |  |  | **GVIF** | **Df** | **GVIF^(1/(2*Df))** |  |  | **GVIF** | **Df** | **GVIF^(1/(2*Df))** |
| Block | 1 | 3 | 1 |  | Block | 1 | 3 | 1 |  | Block | 1 | 3 | 1 |  | Block | 1 | 3 | 1 |
| VPD | 7.153 | 1 | 2.675 |  | maxTemp_1 | 6.823 | 1 | 2.612 |  | RH | 13.961 | 1 | 3.736 |  | VPD | 6.523 | 1 | 2.554 |
| VPDavg | 47.204 | 1 | 6.871 |  | VPD | 7.830 | 1 | 2.798 |  | VPD | 7.940 | 1 | 2.818 |  | Pseudo_EL | 6.863 | 9 | 1.113 |
| Pseudo_EL | 94.084 | 9 | 1.287 |  | Pseudo_EL | 35.919 | 9 | 1.220 |  | Pseudo_EL | 27.150 | 9 | 1.201 |  | Treat | 1.338 | 3 | 1.050 |
| Treat | 1.338 | 3 | 1.050 |  | Treat | 1.338 | 3 | 1.050 |  | Treat | 1.338 | 3 | 1.050 |  | Year | 2.558 | 1 | 1.599 |
| Year | 29.054 | 1 | 5.390 |  | Year | 2.559 | 1 | 1.600 |  | Year | 6.825 | 1 | 2.612 |  | Pseudo_EL:Treat | 2.176 | 27 | 1.015 |
| Pseudo_EL:Treat | 2.176 | 27 | 1.015 |  | Pseudo_EL:Treat | 2.176 | 27 | 1.015 |  | Pseudo_EL:Treat | 2.176 | 27 | 1.015 |  | Treat:Year | 2.075 | 3 | 1.129 |
| Treat:Year | 2.075 | 3 | 1.129 |  | Treat:Year | 2.075 | 3 | 1.129 |  | Treat:Year | 2.075 | 3 | 1.129 |  |  |  |  |  |
| > r.squared  GLMM(moda) | |  |  |  | > r.squared  GLMM(moda) |  |  |  |  | > r.squaredGLMM(moda) |  |  |  |  | >r.squaredGLMM(moda) |  |  |  |
|  | **R^2^m** | **R^2^c** |  |  |  | **R^2^m** | **R^2^c** |  |  |  | **R^2^m** | **R^2^c** |  |  |  | **R^2^m** | **R^2^c** |  |
|  | 0.6077 | 0.6564 |  |  |  | 0.6077 | 0.6564 |  |  |  | 0.6077 | 0.6564 |  |  |  | 0.6080 | 0.6567 |  |

### **Model 4 Diagnostics**

**Final model ANOVA output**

| **2021** | | | | | | | | | | | | | | | **2022** | | | | | | | | | | | | | | | | |
| --- | --- | --- | --- | --- | --- | --- | --- | --- | --- | --- | --- | --- | --- | --- | --- | --- | --- | --- | --- | --- | --- | --- | --- | --- | --- | --- | --- | --- | --- | --- | --- |
| > model21a <- lm(as.formula(paste(var, "~ Treat*Pseudo_EL")), data = df21) | | | | | | | | | | | | | | | > model22a <- lm(as.formula(paste(var, "~ Treat*Pseudo_EL ")), data = df22) | | | | | | | | | | | | | | | | |
| > model21b <- lmer(as.formula(paste(var, "~ Treat*Pseudo_EL+(1\|Vine) ")), data = df21, control = lmerControl(optimizer = "bobyqa"), REML = TRUE) | | | | | | | | | | | | | | | > model22b <- lmer(as.formula(paste(var, "~ Treat*Pseudo_EL + (1\|Vine) ")), data = df22, control = lmerControl(optimizer = "bobyqa"), REML = TRUE) | | | | | | | | | | | | | | | | |
| > model21c <- lme(RC_CS ~ Treat+Pseudo_EL, random = ~ 1 \|Vine, weights = varExp(form = ~ VPD), | | | | | | | | | | | | | | | > model22c <- lme(RC_CS ~ Treat*Pseudo_EL, random = ~1 \|Vine, weights = varIdent(form = ~ Stress2), | | | | | | | | | | | | | | | | |
| + data = df21, control = control_options, method = "REML") | | | | | | | | | | | | | | | + data = df22, control = control_options, method = "REML") | | | | | | | | | | | | | | | | |
| > AICc(model21a, model21b, model21c) | | | | | | | | | | | | | | | > AICc(model22a, model22b, model22c) | | | | | | | | | | | | | | | | |
|  | | | | df | | | | | | AICc | | | | |  | | | | df | | | | | AICc | | | | | | | |
| model21a | | | | 25 | | | | | | 4246.992 | | | | | model22a | | | | 41 | | | | | 7251.200 | | | | | | | |
| **model21b** | | | | **26** | | | | | | **4004.609** | | | | | **model22b** | | | | **42** | | | | | **6708.475** | | | | | | | |
| model21c | | | | 12 | | | | | | 4147.450 | | | | | model22c | | | | 42 | | | | | 6708.475 | | | | | | | |
|  | | | | | | | | | | | | | | |  | | | | | | | | | | | | | | | | |
| **R^2^m** | | | **R^2^c** | | | | | | | | | | | | **R^2^m** | | | **R^2^c** | | | | | | | | | | | | | |
| > r.squaredGLMM(model21a) | | | | | | | | | | | | | | | > r.squaredGLMM(model22a) | | | | | | | | | | | | | | | | |
| [1,] 0.4857567 | | | 0.4857567 | | | | | | | | | | | | [1,] 0.5124698 | | | 0.5124698 | | | | | | | | | | | | | |
| > r.squaredGLMM(model21b) | | | | | | | | | | | | | | | > r.squaredGLMM(model22b) | | | | | | | | | | | | | | | | |
| [1,] 0.4857567 | | | 0.5544824 | | | | | | | | | | | | [1,] 0.5124698 | | | 0.7049501 | | | | | | | | | | | | | |
| > r.squaredGLMM(model21c) | | | | | | | | | | | | | | | > r.squaredGLMM(model22c) | | | | | | | | | | | | | | | | |
| [1,] 0.4934333 | | | 0.5666542 | | | | | | | | | | | | [1,] 0.5124698 | | | 0.7049501 | | | | | | | | | | | | | |
| > model21 <- model21b | | | | | | | | | | | | | | | > model22 <- model22b | | | | | | | | | | | | | | | | |
| > formula(model21) | | | | | | | | | | | | | | | > formula(model22) | | | | | | | | | | | | | | | | |
| RC_CS ~ Treat * Pseudo_EL + (1 \| Vine) | | | | | | | | | | | | | | | RC_CS ~ Treat * Pseudo_EL + (1 \| Vine) | | | | | | | | | | | | | | | | |
| **> AICc(model21)** | | | | | | | | | | | | | | | **> AICc(model22)** | | | | | | | | | | | | | | | | |
| **[1] 4004.609** | | | | | | | | | | | | | | | **[1] 6708.475** | | | | | | | | | | | | | | | | |
| **> r.squaredGLMM(model21)** | | | | | | | | | | | | | | | **> r.squaredGLMM(model22)** | | | | | | | | | | | | | | | | |
| **R^2^m** | | | **R^2^c** | | | | | | | | | | | | **R^2^m** | | | **R^2^c** | | | | | | | | | | | | | |
| **[1,] 0.4857567** | | | **0.5544824** | | | | | | | | | | | | **[1,] 0.5124698** | | | **0.7049501** | | | | | | | | | | | | | |
| > vif(model21) | | | | | | | | | | | | | | | > vif(model22, type = "predictor") | | | | | | | | | | | | | | | | |
|  | | GVIF | | | | Df | | GVIF^(1/(2*Df)) | | | | | | |  | | GVIF | | | | Df | | GVIF^(1/(2*Df)) | | | | | | | | |
| Treat | | 1 | | | | 3 | | 1 | | | | | | | Treat | | 1 | | | | 3 | | 1 | | | | | | | | |
| Pseudo_EL | | 1 | | | | 5 | | 1 | | | | | | | Pseudo_EL | | 1 | | | | 9 | | 1 | | | | | | | | |
| Treat:Pseudo_EL | | 1 | | | | 15 | | 1 | | | | | | | Treat:Pseudo_EL | | 1 | | | | 27 | | 1 | | | | | | | | |
| > sim_res21 <- simulateResiduals(fittedModel = model21) | | | | | | | | | | | | | | | > sim_res22 <- simulateResiduals(fittedModel = model22) | | | | | | | | | | | | | | | | |
| > plot(sim_res21) | | | | | | | | | | | | | | | > plot(sim_res22) | | | | | | | | | | | | | | | | |
| > VarCorr(model21) | | | | | | | | | | | | | | | > VarCorr(model22) | | | | | | | | | | | | | | | | |
| **Groups .** | | **Name** | | | | | | | | **Std.Dev** | | | | | **Groups** | | **Name** | | | | | | | | | | **Std.Dev.** | | | | |
| Vine | | (Intercept) | | | | | | | | 133.77 | | | | | Vine | | (Intercept) | | | | | | | | | | 275.78 | | | | |
| Residual | |  | | | | | | | | 340.59 | | | | | Residual | |  | | | | | | | | | | 341.45 | | | | |
| > anova_table21 <- Anova(model21, type = "III", test = "F") | | | | | | | | | | | | | | | > anova_table22 <- Anova(model22, type = "III", test = "F") | | | | | | | | | | | | | | | | |
| > print(anova_table21) | | | | | | | | | | | | | | | > print(anova_table22) | | | | | | | | | | | | | | | | |
| **Analysis of Deviance Table (Type III Wald F tests with Kenward-Roger df)** | | | | | | | | | | | | | | | **Analysis of Deviance Table (Type III Wald F tests with Kenward-Roger df)** | | | | | | | | | | | | | | | | |
| **Response: RC_CS** | | | | | | | | | | | | | | | **Response: RC_CS** | | | | | | | | | | | | | | | | |
|  | | **F** | | | | **Df** | | **Df.res** | | **Pr(>F)** | |  | | |  | | **F** | | | | **Df** | | **Df.res** | | | **Pr(>F)** | | | |  | |
| (Intercept) | | 24574.6 | | | | 1 | | 44 | | < 2.2e-16 | | *** | | | (Intercept) | | 8600.09 | | | | 1 | | 44 | | | < 2e-16 | | | | *** | |
| **Treat** | | **5.7225** | | | | **3** | | **44** | | **0.002138** | | ****** | | | Treat | | 0.3414 | | | | 3 | | 44 | | | 0.79549 | | | |  | |
| **Pseudo_EL** | | **51.5002** | | | | **5** | | **220** | | **< 2.2e-16** | | ******* | | | **Pseudo_EL** | | **85.9411** | | | | **9** | | **396** | | | **< 2e-16** | | | | ******* | |
| Treat:Pseudo_EL | | 1.4909 | | | | 15 | | 220 | | 0.109943 | |  | | | **Treat:Pseudo_EL** | | **1.8813** | | | | **27** | | **396** | | | **0.00551** | | | | ****** | |
| --- | | | | | | | | | | | | | | | --- | | | | | | | | | | | | | | | | |
| Signif. codes: 0 ‘***’ 0.001 ‘**’ 0.01 ‘*’ 0.05 ‘.’ 0.1 ‘ ’ 1 | | | | | | | | | | | | | | | Signif. codes: 0 ‘***’ 0.001 ‘**’ 0.01 ‘*’ 0.05 ‘.’ 0.1 ‘ ’ 1 | | | | | | | | | | | | | | | | |
| **Results are averaged over the levels of: Pseudo_EL** | | | | | | | | | | | | | |  | | | | | | | | | | | | | | | | |  |
| Degrees-of-freedom method: satterthwaite | | | | | | | | | | | | | | Degrees-of-freedom method: satterthwaite | | | | | | | | | | | | | | | | |  |
| Confidence level used: 0.95 | | | | | | | | | | | | | | P value adjustment: tukey method for comparing a family of 4 estimates | | | | | | | | | | | | | | | | |  |
| > print(pairwise_emm1) | | | | | | | | | | | | | | > print(cld_emm2) | | | | | | | | | | | | | | | | |  |
| **contrast estimate SE df t.ratio p.value** | | | | | | | | | | | | | | **Treat** | | **emmean** | | | | **SE** | | **df** | **Lower.CL** | | **Upper.CL** | | | **group** | | |  |
| Control - A. nodosum -261.6 78.8 44 -3.322 0.0094 | | | | | | | | | | | | | | **Pseudo_EL =EL23:** | | | | | | | | | | | | | | | | |  |
| Control - E. maxima 28.5 78.8 44 0.361 0.9836 | | | | | | | | | | | | | | Control | | 4047 | | | | 127 | | 183 | 3728 | | 4365 | | | | a | |  |
| Control - (NPK-Ref) -130.9 78.8 44 -1.661 0.3560 | | | | | | | | | | | | | | E. maxima | | 4078 | | | | 127 | | 183 | 3760 | | 4397 | | | | a | |  |
| A. nodosum - E. maxima 290.1 78.8 44 3.683 0.0034 | | | | | | | | | | | | | | NPK-Ref | | 4129 | | | | 127 | | 183 | 3810 | | 4447 | | | | a | |  |
| A. nodosum - (NPK-Ref) 130.8 78.8 44 1.660 0.3566 | | | | | | | | | | | | | | A. nodosum | | 4144 | | | | 127 | | 183 | 3825 | | 4463 | | | | a | |  |
| E. maxima - (NPK-Ref) -159.3 78.8 44 -2.023 0.1953 | | | | | | | | | | | | | | **Pseudo_EL =EL27:** | | | | | | | | | | | | | | | | |  |
|  | | | | | | | | | | | | | | A. nodosum | | 4477 | | | | 127 | | 183 | 4158 | | 4795 | | | | a | |  |
| **Results are averaged over the levels of: Pseudo_EL** | | | | | | | | | | | | | | E. maxima | | 4725 | | | | 127 | | 183 | 4407 | | 5044 | | | | a | |  |
| Degrees-of-freedom method: satterthwaite | | | | | | | | | | | | | | NPK-Ref | | 4794 | | | | 127 | | 183 | 4475 | | 5113 | | | | a | |  |
| P value adjustment: tukey method for comparing a family of 4 estimates | | | | | | | | | | | | | | Control | | 4933 | | | | 127 | | 183 | 4615 | | 5252 | | | | a | |  |
| > print(cld_emm1) | | | | | | | | | | | | | | **Pseudo_EL =EL29:** | | | | | | | | | | | | | | | | |  |
| **Treat** | **emmean** | | | | **SE** | | **df** | | **lower.CL** | | **upper.CL** | | **.group** | Control | | 4014 | | | | 127 | | 183 | 3695 | | 4333 | | | | a | |  |
| *E. maxima* | 4246 | | | | 55.7 | | 44 | | 4102 | | 4391 | | b | NPK-Ref | | 4058 | | | | 127 | | 183 | 3739 | | 4377 | | | | a | |  |
| Control | 4275 | | | | 55.7 | | 44 | | 4130 | | 4419 | | b | E. maxima | | 4255 | | | | 127 | | 183 | 3936 | | 4574 | | | | a | |  |
| NPK-Ref | 4406 | | | | 55.7 | | 44 | | 4261 | | 4550 | | ab | A. nodosum | | 4259 | | | | 127 | | 183 | 3940 | | 4577 | | | | a | |  |
| *A. nodosum* | 4536 | | | | 55.7 | | 44 | | 4392 | | 4681 | | a | **Pseudo_EL =EL31:** | | | | | | | | | | | | | | | | |  |
|  | | | | | | | | | | | | | | Control | | 4023 | | | | 127 | | 183 | 3704 | | 4342 | | | | a | |  |
| Results are averaged over the levels of: Pseudo_EL | | | | | | | | | | | | | | NPK-Ref | | 4176 | | | | 127 | | 183 | 3858 | | 4495 | | | | a | |  |
| Degrees-of-freedom method: satterthwaite | | | | | | | | | | | | | | A. nodosum | | 4253 | | | | 127 | | 183 | 3935 | | 4572 | | | | a | |  |
| Confidence level used: 0.95 | | | | | | | | | | | | | | E. maxima | | 4275 | | | | 127 | | 183 | 3956 | | 4593 | | | | a | |  |
| Conf-level adjustment: sidak method for 4 estimates | | | | | | | | | | | | | | **Pseudo_EL =EL32:** | | | | | | | | | | | | | | | | |  |
| P value adjustment: tukey method for comparing a family of 4 estimates | | | | | | | | | | | | | | Control | | 3901 | | | | 127 | | 183 | 3582 | | 4220 | | | | a | |  |
| significance level used: alpha = 0.05 | | | | | | | | | | | | | | NPK-Ref | | 4023 | | | | 127 | | 183 | 3704 | | 4341 | | | | a | |  |
|  | | | | | | | | | | | | | | A. nodosum | | 4107 | | | | 127 | | 183 | 3789 | | 4426 | | | | a | |  |
|  | | | | | | | | | | | | | | E. maxima | | 4173 | | | | 127 | | 183 | 3854 | | 4491 | | | | a | |  |
|  | | | | | | | | | | | | | | **Pseudo_EL =EL32b:** | | | | | | | | | | | | | | | | |  |
|  | | | | | | | | | | | | | | Control | | 4085 | | | | 127 | | 183 | 3766 | | 4404 | | | | a | |  |
|  | | | | | | | | | | | | | | NPK-Ref | | 4248 | | | | 127 | | 183 | 3929 | | 4566 | | | | a | |  |
|  | | | | | | | | | | | | | | A. nodosum | | 4255 | | | | 127 | | 183 | 3936 | | 4574 | | | | a | |  |
|  | | | | | | | | | | | | | | E. maxima | | 4429 | | | | 127 | | 183 | 4110 | | 4747 | | | | a | |  |
|  | | | | | | | | | | | | | | **Pseudo_EL =EL33b:** | | | | | | | | | | | | | | | | |  |
|  | | | | | | | | | | | | | | Control | | 3830 | | | | 127 | | 183 | 3511 | | 4148 | | | | a | |  |
|  | | | | | | | | | | | | | | A. nodosum | | 3901 | | | | 127 | | 183 | 3582 | | 4219 | | | | a | |  |
|  | | | | | | | | | | | | | | NPK-Ref | | 3920 | | | | 127 | | 183 | 3602 | | 4239 | | | | a | |  |
|  | | | | | | | | | | | | | | E. maxima | | 4006 | | | | 127 | | 183 | 3687 | | 4324 | | | | a | |  |
|  | | | | | | | | | | | | | | **Pseudo_EL =EL34:** | | | | | | | | | | | | | | | | |  |
|  | | | | | | | | | | | | | | Control | | 3315 | | | | 127 | | 183 | 2997 | | 3634 | | | | a | |  |
|  | | | | | | | | | | | | | | A. nodosum | | 3481 | | | | 127 | | 183 | 3163 | | 3800 | | | | a | |  |
|  | | | | | | | | | | | | | | NPK-Ref | | 3508 | | | | 127 | | 183 | 3190 | | 3827 | | | | a | |  |
|  | | | | | | | | | | | | | | E. maxima | | 3550 | | | | 127 | | 183 | 3231 | | 3868 | | | | a | |  |
|  | | | | | | | | | | | | | | **Pseudo_EL =EL34b:** | | | | | | | | | | | | | | | | |  |
|  | | | | | | | | | | | | | | Control | | 3578 | | | | 127 | | 183 | 3260 | | 3897 | | | | a | |  |
|  | | | | | | | | | | | | | | E. maxima | | 3692 | | | | 127 | | 183 | 3374 | | 4011 | | | | a | |  |
|  | | | | | | | | | | | | | | NPK-Ref | | 3795 | | | | 127 | | 183 | 3476 | | 4113 | | | | a | |  |
|  | | | | | | | | | | | | | | A. nodosum | | 3828 | | | | 127 | | 183 | 3509 | | 4146 | | | | a | |  |
|  | | | | | | | | | | | | | | **Pseudo_EL =EL35:** | | | | | | | | | | | | | | | | |  |
|  | | | | | | | | | | | | | | A. nodosum | | 2795 | | | | 127 | | 183 | 2476 | | 3114 | | | | a | |  |
|  | | | | | | | | | | | | | | NPK-Ref | | 3009 | | | | 127 | | 183 | 2690 | | 3327 | | | | ab | |  |
|  | | | | | | | | | | | | | | E. maxima | | 3124 | | | | 127 | | 183 | 2805 | | 3443 | | | | ab | |  |
|  | | | | | | | | | | | | | | Control | | 3380 | | | | 127 | | 183 | 3061 | | 3699 | | | | b | |  |
|  | | | | | | | | | | | | | | Degrees-of-freedom method: satterthwaite | | | | | | | | | | | | | | | | |  |
|  | | | | | | | | | | | | | | Confidence level used: 0.95 | | | | | | | | | | | | | | | | |  |
|  | | | | | | | | | | | | | | Conf-level adjustment: sidak method for 4 estimates | | | | | | | | | | | | | | | | |  |
|  | | | | | | | | | | | | | | P value adjustment: tukey method for comparing a family of 4 estimates | | | | | | | | | | | | | | | | |  |
|  | | | | | | | | | | | | | | significance level used: alpha = 0.05 | | | | | | | | | | | | | | | | |  |
