# Supplementary material for: Assessing the potential of seaweed extracts to improve vegetative, physiological and berry quality parameters in Vitis vinifera cv. Chardonnay under cool climatic conditions
Source: PLoS One. 2025 Sep 2;20(9):e0331039. doi: 10.1371/journal.pone.0331039 (PMC12404493; doi:10.1371/journal.pone.0331039)
Supplement: Attachment — Submitted filename: Response to Reviewers_v2.docx [file pone.0331039.s017.docx]

Dear Editor,

Please find attached our revised manuscript ‘Assessing the potential of seaweed extracts to improve vegetative, physiological and berry quality parameters in *Vitis vinifera* cv. Chardonnay under cool climatic conditions’.

We thank the referees for their positive feedback on the updated results and discussion following the first round of revision, as well as for the additional comments on the latest version of the manuscript (PONE-D-24-45452R1). We have reviewed the comments and implemented them insofar was possible. Given their complementary nature, comments are grouped per reviewer and addressed individually as follows:

- Response to the *global comments* of the editor and reviewers p. 2
- Responses to *specific comments* per reviewer, followed by the authors’ response or corrective action p. 2-5.
  - Minor comments are specified as ‘implemented as suggested’.
  - Responses are also linked to lines in the updated **Revised manuscript with Tracked changes enabled**.
- *Additional changes* relating to clarity and flow p. 5.

In addition to a clean version of the revised manuscript, a version with changes marked through ‘Track Changes’, has been uploaded; this version details grammatical and typographic improvements.

We hope that you find this revised version suitable for publication in PLOS ONE and we look forward to a positive evaluation for publication.

Sincerely,

Prof. Dr. Sam Crauwels

CMPG Laboratory for Process Microbial Ecology and Bioinspirational Management (PME&BIM)

Department of Microbial and Molecular Systems (M2S), KU Leuven

Willem De Croylaan 46, B-3001 Leuven, Belgium

E-mail: [sam.crauwels@kuleuven.be](mailto:sam.crauwels@kuleuven.be)

# Global comments

**EDITOR:** Thank you for submitting your manuscript to PLOS ONE. After careful consideration, we believe that it has merit but still requires some minor amendments to meet the quality criteria of PLOS ONE. Both reviewers acknowledged the quality of your revision. Therefore, we invite you to submit a revised version of the manuscript that addresses the remaining points raised during the review process.

**REVIEWER 1:** The manuscript improved a lot, it’s almost a whole new paper. The authors have addressed the vast majority of the comments of both reviewers very diligently and competently, with a special emphasis on Discussion and Statistics, where they did an enormous and great job. I have no doubt that it’s adequate for publication in PLOS. I still have some specific comments on few aspects that I’m sure will be easy to handle.

**REVIEWER 2:** Thank you for revising your manuscript according to the reviewers’ suggestions. This version shows significant improvement compared to the previous one, particularly in the Results and Discussion sections. Please find below a few minor comments for further adjustment (line numbers relative to the MS file with track-changes ON).

***Authors:*** *We thank the editor and reviewers for their positive feedback regarding the improved results and discussion sections, as well as for their additional comments and suggestions, and appreciate the time and thoroughness with which they have evaluated the manuscript.*

# Specific comments

**REVIEWER 1**

Regarding the need for a better justification for focusing on cooler/moist regions and the eventual contribution of seaweed extracts on the mitigation of potential stresses, an important point raised by the editor and the reviewers, and addressed by the authors (lines 101-105), I still think that, although they indicated that “these regions are challenged by variable weather conditions as well as extreme weather events” (what about the hotter/drier regions?) and that grapevines may be “particularly vulnerable showing delayed ripening and increased disease incidence”, they didn’t explain and it’s not clear “in what way seaweed extracts may help to overcome” those problems in particular (already on my previous specific comments - lines 81- 83). The authors add that “seaweed may mitigate stress by regulating photosynthesis, enhancing WUE and nutrient uptake”, but: i) regulating Pn in what sense? Increasing?; ii) is there a problem with WUE in cooler/more humid regions? I don’t think so; iii) or with NU? And even if so, how to link an increase in WUE and NU with a less delayed ripening or less susceptibility to diseases? I call attention for the fact that ref. 47 does not address stress in cool/ humid regions, but mainly the benefits of A. nodosum extracts in plants under drought stress. Having said this, I think that the authors can accommodate these issues putting the tonic on that the addition of seaweeds extracts (namely A. nodosum) can “stimulate natural processes to enhance/benefit nutrient uptake, nutrient efficiency, tolerance to abiotic stresses (47)”, and that grapevines well fertilized an in better physiological status are also better prepared to respond to biotic as well as abiotic challenges. All in all, it is important to clearly refer to the stresses/challenges that grapevine varieties can endure in cool/humid regions, so it would be reasonable to accept that the extracts could, in fact, help alleviating from/avoiding injury caused by the stress factors.

- The authors agree the benefits of the seaweed extracts as mentioned were not clearly linked to specific outcomes related to cool-climate environmental stressors (sensitivity to increased heat/drought stress; delayed ripening in cooler climates) and acknowledge the difficulty in drawing these links. We therefore welcome the reviewer’s suggestion to first place emphasis on ‘seaweed extracts stimulating natural processes…’, then stating that well-fertilised vines in a good physiological condition are better prepared to respond to biotic/abiotic stressors. We also removed the general effect of ‘improved WUE’, as it’s less relevant in this cool-climate context.
- L98-102 in updated track changes MS

Line 101 – add examples of “extreme events”.

Line 103 – rephrase, I cannot understand exactly what do you want to say with this sentence – i) what “stress conditions”?; ii) and “similar” to what?; iii) “experienced by grapevine varieties that are more adapted to these conditions”, if by “these conditions” you mean cool/moist ones, how can varieties more adapted to those conditions experience stress?

- We agree with the reviewer that the phrasing of this section is somewhat confusing. Clarified that extreme events (such as heat stress and drought) occur with increasing frequency, and specified that cool-climate vines are less adapted to these events than their warm-climate counterparts. Furthermore, clearly mentioned challenges that are more prevalent in cool climate regions (delayed ripening & increased disease incidence), and in line with the first comment, refer to seaweed extract effects that may improve grapevine tolerance to cool-climate environmental stressors.
- L92-98 in updated track changes MS

Line 126 – don’t start a sentence with numerals or abbreviations – use Ecklonia in full.

- Done as suggested, similarly for *A. nodosum*– screened entire manuscript to ensure sentences starting with species names have the species names written out in full.

Lines 271 – reformulate adding what I’ve underligned - “xxx dark adapted for 30 min to open the RC and record minimal fluorescence (F0) and subject to xxx to obtain maximum fluorescence (Fm) and compute Fv/Fm. Fv/Fm =(Fm−F0)/Fm

- Reformulated to specify dark-adaptation was done to ensure all PSII RCs were open allowing for the estimation of the minimal fluorescence F_0_, also added small formula inline.
- L271-276 in updated track changes MS

Accordingly to the above comment, it is important to include the procedure to calculate FPSII (=(F′m−Fs)/F′m). This parameter is measured in light-adapted plants/leaves, so the AL intensity and duration of the light adaptation period before another saturation pulse (or flash) is given is important to include.

- Thank you for your careful attention to the fluorescence parameters. We acknowledge there was a mix-up in the original manuscript: we inadvertently referred to the effective quantum yield of PSII (ΦPSII or FPSII), when in fact all measurements were made on dark-adapted samples using the PocketPEA, and thus the parameter reported is the quantum yield for electron transport beyond QA⁻ (PSII electron transport efficiency, Φ_E0_), as derived from the OJIP fluorescence induction curve. This has been clarified in the methods and corrected throughout Figure 3, the relevant results section, and the discussion to accurately reflect the parameter measured and reported.
- L267-268 & L500-511 in updated track changes MS

Line 424 – “were no significant differences in shoot length between xxx”

- Implemented as suggested

Line 962 – don’t use “photosynthetic capacity” because it has a proper meaning and you didn’t measure/calculated this parameter - substitute by the parameters you’ve measured or say just “photochemical efficiency”, what can be a general expression that may englobe Fv/Fm and ФPSII

- Implemented as suggested

**REVIEWER 2**

L24 – Remove “-based”

- Updated to ‘derived from *Ascophyllum nodosum*…’

L27 – Use “levels” instead of “level”

- Implemented as suggested

L33 – Rephrase to: “improvement of PSII maximum efficiency...”

- Retained PSII maximum quantum yield (F_v_/F_m_) to ensure clarity and avoid confusion with PSII electron transport efficiency Φ_E0._

L35–36 – Please clarify: this increase was in response to which treatment? Ascophyllum nodosum?

- Clarified that increase was in response to *A. nodosum* treatment.
- L33-34 in updated track changes MS

L42–43 – Rephrase “cooler climatic conditions in a cool climate region”; it is repetitive.

- Implemented as suggested

L98 – Use “focused” rather than “not focused”

- Implemented as suggested

L163–165 – Just to confirm: was it not possible to balance P levels between the NPK fertilizer and the extracts?

- The authors recognise this as a minor shortcoming, however with the available commercial fertilisers it was not possible to balance all three components. Additionally, the NPK composition differed between *A. nodosum* and *E. maxima* so the authors opted to target the highest values between the two extracts, resulting in similar N and K levels as found in *A. nodosum* and a P-level twice that of the *E. maxima* extract.
- L162-165 in updated track changes MS

L173 – Remove “during 2021”

- Implemented as suggested

L177–180 – While the rationale for different application periods is clear, the change in application frequency should also be explained.

- The authors rephrased this section to more clearly illustrate that specific phenological stages were targeted, and as the rate of phenological progression is influenced by climate conditions, the warmer 2022 season resulted in more frequent applications (to ensure the same stages are targeted as in 2021). In the end, both seasons received 5 applications at similar phenological development stages.
- L175-180 in updated track changes MS

L802 – Please note that auxins are more commonly associated with root growth; I assume the sentence is the other way around.

- Implemented as suggested

L960 – Clarify that the P rate was not the same across treatments.

- Rephrased section to clearly state N and K matched *A. nodosum* (the richest species in these elements), while P in the synthetic NPK solution was 2x that measured in *E. maxima* and 20x that measured in *A. nodosum*.
- L766-770 in updated track changes MS

In addition, I noticed some typographical and grammatical errors throughout the manuscript. I recommend a careful proofreading and a thorough round of language editing to ensure clarity and correctness.

- The authors have conducted a thorough review of the manuscript to address these issues. Some systematic changes and corrections are detailed under ‘Additional Changes,’ while minor edits have been made throughout the text and are visible as tracked changes in the revised document.

# Additional changes

- **Consistent SI-compliant formatting** of all units (e.g., g L⁻¹, 20 °C), with a space between numbers and unit symbols per SI style.
- **Systematic addition of non-breaking spaces and hyphens** for all sensitive values (e.g., *A. nodosum*, *n* = 4, *P* = 0.05, mean ± SE, units, and between numbers and °C), to ensure clarity and prevent awkward line breaks in text, tables, and figures.
- **Harmonisation of scientific names and statistics:** All species names (*A. nodosum, E. maxima*) are now consistently italicised; sample sizes, *P*-values, and other abbreviations are formatted and spaced uniformly.
- **Spelling and language consistency:** Confirmed British English is used throughout (e.g., “analyze” → “analyse” and corrected isolated spelling or typographical errors (e.g., “phosphorous” → “phosphorus”).
- **Statistical terminology:** Removed possessives in adjustment/method names (e.g., “Satterthwaite degrees of freedom adjustment”), in line with current statistical usage.
- **Clarified and updated table footnotes** for statistical methods and tests, and ensured figure/table legends correctly describe whether data shown are means or model-based EMMs.
- **Ensured clear distinction between raw data and model-based inferences** in both tables and Results text; statistical inferences and *P*-values are based on EMMs, while raw means are provided for descriptive context.
- **Systematic update and harmonisation of supplementary tables:**
  - Included all relevant parameters (e.g., RC/CS, ΦE₀ in S4 Table).
  - Added “Average” rows of raw data where appropriate, to align supplementary data structure with model outputs and facilitate interpretation.
  - Updated formatting on all supplementary tables to ensure consistent layout.
- **Thorough review of reference list for formatting and missing information**
